# Supplementary material for: Understanding the biology, morbidity and social contexts of adolescent tuberculosis: a prospective observational cohort study protocol (Teen TB)
Source: BMJ Open. 2022 Dec 19;12(12):e062979. doi: 10.1136/bmjopen-2022-062979 (PMC9772637; doi:10.1136/bmjopen-2022-062979)
Supplement: Author's manuscript [file bmjopen-2022-062979.draft_revisions.pdf]

BMJ Open

BMJ Open is committed to open peer review. As part of this commitment we make the peer review history of every article we publish publicly available.

When an article is published we post the peer reviewers' comments and the authors' responses online. We also post the versions of the paper that were used during peer review. These are the versions that the peer review comments apply to.

The versions of the paper that follow are the versions that were submitted during the peer review process. They are not the versions of record or the final published versions. They should not be cited or distributed as the published version of this manuscript.

BMJ Open is an open access journal and the full, final, typeset and author-corrected version of record of the manuscript is available on our site with no access controls, subscription charges or pay-per-view fees (http://bmjopen.bmj.com).

If you have any questions on BMJ Open's open peer review process please email info.bmjopen@bmj.com

Understanding the biology, morbidity and social contexts of adolescent tuberculosis: a prospective observational cohort study protocol (Teen TB)

Journal:	*BMJ Open*
Manuscript ID	bmjopen-2022-062979
Article Type:	Protocol
Date Submitted by the Author:	16-Mar-2022

Complete List of Authors:	Swanepoel, Jeremi; Desmond Tutu TB Centre, Department of Paediatrics and Child Health, Faculty of Medicine and Health Sciences, Stellenbosch University; London School of Hygiene & Tropical Medicine<br>Zimri, Klassina; Desmond Tutu TB Centre, Department of Paediatrics and Child Health, Faculty of Medicine and Health Sciences, Stellenbosch University<br>van der Zalm, Marieke; Desmond Tutu TB Centre, Department of Paediatrics and Child Health, Faculty of Medicine and Health Sciences, Stellenbosch University<br>Hoddinott, Greame; Desmond Tutu TB Centre, Department of Paediatrics and Child Health, Faculty of Medicine and Health Sciences, Stellenbosch University<br>Palmer, Megan ; Desmond Tutu TB Centre, Department of Paediatrics and Child Health, Faculty of Medicine and Health Sciences, Stellenbosch University<br>De Beer, Gezila; Desmond Tutu TB Centre, Department of Paediatrics and Child Health, Faculty of Medicine and Health Sciences, Stellenbosch University<br>Kleynhans, Leanie; DSI-NRF Centre of Excellence, South African Medical Research Council Centre for Tuberculosis Research, Division of Molecular Biology and Human Genetics, Department of Biomedical Sciences, Faculty of Medicine and Health Sciences, Stellenbosch University<br>Johnson, Sarah; Imperial College London, Department of Infectious Diseases<br>Jongen, Vita; Public Health Service of Amsterdam, Department of Infectious Diseases<br>Wademan, Dillon; Desmond Tutu TB Centre, Department of Paediatrics and Child Health, Faculty of Medicine and Health Sciences, Stellenbosch University<br>Mcimeli, Khanyisa; Desmond Tutu TB Centre, Department of Paediatrics and Child Health, Faculty of Medicine and Health Sciences, Stellenbosch University<br>Jacobs, Stephanie; Desmond Tutu TB Centre, Department of Paediatrics and Child Health, Faculty of Medicine and Health Sciences, Stellenbosch University<br>Swanepoel, Ruan; Tygerberg Hospital, Division of Pulmonology, Department of Internal Medicine<br>U Van Zyl, Gert; Stellenbosch University Faculty of Medicine and Health Sciences, Division of Medical Virology, Department of Pathology

Allwood, Brian; Stellenbosch University Faculty of Medicine and Health Sciences, Division of Pulmonology, Department of Medicine
Malherbe, Stephanus; DSI-NRF Centre of Excellence, South African Medical Research Council Centre for Tuberculosis Research, Division of Molecular Biology and Human Genetics, Department of Biomedical Sciences, Faculty of Medicine and Health Sciences, Stellenbosch University
Griffith-Richards, Stephanie; Stellenbosch University Faculty of Medicine and Health Sciences, Division of Radiodiagnosis, Department of Medical Imaging and Clinical Oncology
Whittaker, Elizabeth ; Imperial College London, Department of Infectious Diseases
Moore, Dave; London School of Hygiene and Tropical Medicine Faculty of Infectious and Tropical Diseases
Schaaf, Simon; Desmond Tutu TB Centre, Department of Paediatrics and Child Health, Faculty of Medicine and Health Sciences, Stellenbosch University
Hesseling, A C; Desmond Tutu TB Centre, Department of Paediatrics and Child Health, Faculty of Medicine and Health Sciences, Stellenbosch University
Seddon, James; Desmond Tutu TB Centre, Department of Paediatrics and Child Health, Faculty of Medicine and Health Sciences, Stellenbosch University; Imperial College London, Department of Infectious Diseases

Keywords: Tuberculosis < INFECTIOUS DISEASES, Paediatric infectious disease & immunisation < PAEDIATRICS, Paediatric thoracic medicine < PAEDIATRICS

SCHOLARONE™
Manuscripts

TITLE PAGE

Manuscript Title

Understanding the biology, morbidity and social contexts of adolescent tuberculosis: a prospective observational cohort study protocol (Teen TB)

Authors

Jeremi Swanepoel[1,2]
Klassina Zimri[1]
Marieke M. van der Zalm[1]
Graeme Hoddinott[1]
Megan Palmer[1]
Alex Doruyter[3,4]
Gezila de Beer[1]
Léanie Kleynhans[5]
Sarah M. Johnson[6]
Vita Jongen[7]
Dillon Wademan[1]
Khanyisa Mcimeli[1]
Stephanie Jacobs[1]
Ruan Swanepoel[8]
Gert van Zyl[9]
Brian Allwood[10]
Stephanus Malherbe[5]
Charlotte Heuvelings[1]
Stephanie Griffith-Richards[11]
Elizabeth Whittaker[6]
David A. J. Moore[2]
H Simon Schaaf[1]
Anneke C. Hesseling[1]
James A. Seddon[1,6]

For peer review only

Affiliations

[1]Desmond Tutu TB Centre, Department of Paediatrics and Child Health, Faculty of Medicine and Health Sciences, Stellenbosch University, South Africa

[2]TB Centre, London School of Hygiene and Tropical Medicine, London, United Kingdom

[3]NuMeRI Node for Infection Imaging, Central Analytical Facilities, Stellenbosch University

[4]Division of Nuclear Medicine, Stellenbosch University

[5]DSI-NRF Centre of Excellence for Biomedical Tuberculosis Research, South African Medical Research Council Centre for Tuberculosis Research, Division of Molecular Biology and Human Genetics, Department of Biomedical Sciences, Faculty of Medicine and Health Sciences, Stellenbosch University, Cape Town, South Africa

[6]Department of Infectious Diseases, Imperial College London, London, United Kingdom

[7]Department of Infectious Diseases, Public Health Service Amsterdam, Amsterdam, The Netherlands

[8]Division of Pulmonology, Department of Internal Medicine, Tygerberg Academic Hospital

[9]Division of Medical Virology, Stellenbosch University and NHLS Tygerberg, Cape Town, South Africa

[10]Division of Pulmonology, Department of Medicine, Faculty of Medicine and Health Sciences, Stellenbosch University, South Africa

[11]Division of Radiodiagnosis, Department of Medical Imaging and Clinical Oncology, Faculty of Medicine and Health Sciences, Stellenbosch University

Keywords

Tuberculosis

Adolescents

Lung Function

Puberty

Stigma

Word Count

Abstract: 261

Main text: 3548

For peer review only

Corresponding Author

James Seddon

Desmond Tutu TB Centre

Department of Paediatrics and Child Health

Faculty of Medicine and Health Sciences

Stellenbosch University

South Africa

jseddon@sun.ac.za

For peer review only

ABSTRACT

Introduction:

A considerable burden of the tuberculosis (TB) epidemic is found in adolescents. The reasons for increased susceptibility to TB infection and higher incidence of TB disease in adolescence, compared to the 5 to 10-year-old age group, are incompletely understood. Despite the pressing clinical and public health need to better understand and address adolescent TB, research in this field remains limited.

Methods and analysis:

Teen TB is an ongoing prospective observational cohort study that aims to better understand the biology, morbidity and social context of adolescent TB. The study plans to recruit 50 adolescents (10-19-years-old) with newly diagnosed microbiologically confirmed pulmonary TB disease and 50 TB-exposed controls without evidence of TB disease in Cape Town, South Africa, which is highly endemic for TB. At baseline, cases and controls will undergo a detailed clinical evaluation, chest imaging, respiratory function assessments and blood collection for viral co-infections, inflammatory cytokines and pubertal hormone testing. At 2 weeks, 2 months and 12 months, TB disease cases will undergo further chest imaging and additional lung function testing to explore the patterns of respiratory abnormalities. At week 2, cases will complete a multi-component quantitative questionnaire about psychological and social impacts on their experiences and longitudinal, in-depth qualitative data will be collected from a nested sub-sample of 20 cases and their families.

Ethics and dissemination:

The study protocol has received ethical approval from the Stellenbosch University Health Research Ethics Committee (N19/10/148). The study findings will be disseminated through peer-reviewed publications, academic conferences and formal presentations to health professionals. Results will also be made available to participants and caregivers.

For peer review only

STRENGHTS AND LIMITATIONS OF THIS STUDY

- The Teen TB study will provide a unique platform to explore the patterns of structural and functional respiratory abnormalities in adolescent pulmonary TB.
- Results of this study may contribute to a better understanding of the impact of pubertal hormones and viral co-infections on the human immune response to *Mycobacterium tuberculosis.*
- The study will provide an improved understanding of the sociological and psychological impact of TB on adolescents and will also inform the design of more adolescent-friendly TB health services.
- The small sample size and susceptibility to loss to follow up are limitations of this study.

For peer review only

INTRODUCTION

There are an estimated 1.6 billion adolescents (aged 10 to 19 years) living in the world today, with 90% in low- and middle-income countries (LMICs).[1,2] Modelling studies suggest that 750,000 adolescents develop tuberculosis (TB) disease each year globally,[3] yet adolescent TB remains a neglected field of research. Adolescents have unique biological and social circumstances that differ from both younger children and adults. These include hormonal and immunological pubertal changes, increasing autonomy, extensive socialisation and risk-taking behaviour. These unique features have been poorly studied in resource-limited settings, particularly in relation to their impact on health.

For reasons incompletely understood, the risk of progression from TB infection to TB disease rises in adolescence.[4] This may be related to the impact of puberty on the immunological response to *Mycobacterium tuberculosis (Mtb)*,[4] or due to co-infections, nutrition and substance abuse (alcohol, smoking, illicit drugs) impacting on TB pathogenesis, as well as changes in social life impacting on exposure risks. Adolescents tend to develop infectious, cavitary forms of disease,[5–9] thus having a greater impact than younger children on TB transmission. Furthermore, there is extensive social mixing,[3,5] late presentation of TB disease,[10] poor adherence to treatment and follow up and high rates of loss to follow up.[11] These may all have a substantial impact on TB within the adolescent population and on propagation of the epidemic.[6] Outcomes for adolescents with TB are worse than most other population groups.[12] Treatment challenges are more complex when multidrug-resistant TB (MDR-TB; defined as disease caused by *Mtb* resistant to at least rifampicin and isoniazid) is present or when there is HIV co-infection.[13] Finally, the impact of TB on respiratory morbidity in adolescents is almost entirely unexplored. Emerging evidence in adults demonstrates that more than half of individuals develop clinically relevant respiratory morbidity post pulmonary TB,[14,15] and there is evolving appreciation that respiratory insults in childhood and adolescence have lifelong consequences.[16,17] Any functional impairment may have an impact on their quality of life, their future educational and employment potential, as well as their long-term health.[18,19] This illustrates the importance of exploring both pulmonary function and psychosocial aspects of adolescent TB in research.

TB has been identified as a key research priority for adolescent health in LMICs,[20] and adolescent TB is a crucial component of the revised World Health Organization (WHO) Roadmap for ending TB.[21] We therefore set out to conduct a study to examine the biology, morbidity and psychosocial contexts of adolescent TB, named Teen TB. This study will provide a vehicle to explore how these different elements of adolescent TB interact. Teen TB will also allow an unparalleled opportunity to explore the patterns of abnormalities found on chest radiography, ultrasound imaging and ^{18}F-fluorodeoxyglucose (FDG) Positron Emission Tomography (PET) combined with chest radiography and chest computed tomography (CT) in adolescents with TB. In addition, it will enable these findings to be correlated with

For peer review only

the extent of respiratory deficit, social contexts and biological markers. Understanding this will inform our understanding of adolescent TB treatment, adherence and transmission and will have implications for future interventional studies.

Study objectives

The Teen TB study has three main objectives. The first is to evaluate the relationship between baseline lung imaging (advanced and point-of-care) and respiratory function in adolescents with TB disease. We aim to test the hypothesis that imaging at the time of diagnosis can predict future lung function. The second objective is to explore the psychosocial experience of adolescents affected by drug-susceptible (DS) and MDR-TB and evaluate if the diagnosis of TB is associated with experiences of stigma. The third objective is to explore how pubertal hormones and viral co-infections (notably cytomegalovirus [CMV]) influence the immune response to *Mtb.* We hypothesise that as adolescents pass through puberty, they generate an increasingly pronounced myeloid inflammatory response to *Mtb*.

METHODS AND ANALYSIS

Study design and setting

Teen TB is a prospective observational cohort study which plans to recruit 50 adolescents (10 to 19 years) with newly diagnosed microbiologically confirmed TB disease (including both MDR-TB and DS-TB) in Cape Town, Western Cape, South Africa and follow them over a 12-month period. We will also recruit 50 TB-exposed controls (exposed to both DS-TB and MDR-TB). South Africa's Western Cape province is highly endemic for TB and previous studies have shown a substantial burden of TB among adolescents in this setting.[12] The overall TB incidence rate in the Western Cape was 681/100,000 in 2015 and the antenatal HIV prevalence in the public sector antenatal clinics was 18.5% for the province in the same year.[22,23] This high burden setting is an ideal place to carry out adolescent TB studies to meet research gaps.

Study participants

Any adolescent with newly diagnosed pulmonary TB bacteriologically confirmed on sputum (Xpert- or culture-positive), with or without HIV co-infection and who are within the first 14 days of treatment, are eligible to participate in this study. Any adolescent exposed in their household to a case of infectious pulmonary TB and who has no symptoms of TB, will be considered for the TB-exposed control group. Those with TB disease will be excluded from the study if they have extrapulmonary TB without evidence of pulmonary TB. TB-exposed controls will be excluded if they have previously recovered from TB disease. Both participants with TB disease and TB exposure will be excluded if they have a severe illness or any condition causing the adolescent to be clinically unstable or requiring intensive care

For peer review only

treatment, if pregnant or breastfeeding, if known to have diabetes mellitus and if HIV testing is declined with no recent (<12 months) HIV test result available (**Table 1**).

Table 1. Inclusion and exclusion criteria for Teen TB

	**Inclusion**	**Exclusion**
**Tuberculosis disease**	Any adolescent (10 to 19 years) who:<br>• Has a primary diagnosis of newly diagnosed pulmonary TB bacteriologically confirmed on sputum (Xpert- or culture-positive), with or without HIV coinfection<br>• Is within the first 14 days since diagnosis and thus 14 days of TB treatment	• Extrapulmonary TB without evidence of pulmonary TB<br>• Severe illness or any condition causing the adolescent to be clinically unstable or require intensive care treatment<br>• Pregnancy or breastfeeding (due to exposure to ionizing radiation)<br>• Diabetes mellitus (due to the potential confounding effects of hyperglycaemia and insulin levels on ^{18}F-FDG uptake on PET/CT)<br>• Participants declining HIV testing for whom a recent (<12 month) HIV test result is not available
**Tuberculosis exposure**	Any adolescent (10 to 19 years) who:<br>• Has been exposed in their household in the last 6 months to a case of infectious pulmonary TB<br>• Has no symptoms of TB disease	• Previous TB disease<br>• Severe illness or any condition causing the adolescent to be clinically unstable or require intensive care treatment<br>• Pregnancy or breastfeeding (to avoid any confounding in comparison with cases)<br>• Diabetes mellitus (to avoid any confounding in comparison with cases)<br>• Participants declining HIV testing for whom a recent (<12 month) HIV test result is not available

TB: tuberculosis; ^{18}F-FDG: 18F-fluorodeoxyglucose; PET/CT: positron emission tomography and computed tomography; HIV: Human Immunodeficiency Virus

For peer review only

Recruitment Process

DS-TB recruitment will be carried out in collaboration with clinical staff in health facilities in the Tygerberg, Mitchell's Plain and Khayelitsha sub-districts of the City of Cape Town Metropole. As adolescents present to health facilities in these sub-districts with TB symptoms, we will approach them and sensitise them to the study. If they are diagnosed with DS-TB and meet the eligibility criteria, we will approach them prior to starting TB treatment to see if they would be willing to participate. If willing to participate, informed written consent will be obtained from parents/legal guardians for those under 18 years with additional assent from the adolescent, whereas 18- and 19-year-olds can provide consent for themselves. Initial consultations will take place at their homes, local clinics or at Desmond Tutu TB Centre (DTTC) field sites, as determined to be most convenient to the participant and their family. Given the necessity for all participants to undertake lung function testing at Tygerberg Hospital and to undergo ^{18}F-FDG PET-CT imaging for those with TB disease, the follow-up visit will be at the iKamva Unit on the Tygerberg Campus of Stellenbosch University (SU).

MDR-TB recruitment will be carried out using the established TB-CHAMP platform to identify adolescents aged 10-19 years in the Metropolitan area who are newly diagnosed with MDR-TB disease. TB-CHAMP is an ongoing MDR-TB prevention trial that randomises children who have been exposed to MDR-TB in their household to levofloxacin or placebo, with three South African sites, and in collaboration with the MRC Clinical Trials Unit at University College London. The TB-CHAMP trial team identifies every MDR-TB patient in the Cape Town Metropolitan area through laboratory and clinical sources. This is an established platform by which adolescent MDR-TB cases can be identified. We will then, using our established links with routine services, and in collaboration with their healthcare providers, contact these adolescents, following sensitisation to the study by local clinics, ideally prior to starting MDR-TB treatment, and invite them to join the study.

TB-exposed controls are recruited from the same clinics where individuals with TB disease are recruited, and the contacts of the enrolled cases will also be eligible for inclusion. TB-exposed controls must have been exposed to an infectious DS-TB case within the last 6 months. We will again use the TB-CHAMP platform to aid in identifying adolescents who have been exposed to an infectious pulmonary MDR-TB case in the household within the last 6 months.

Study Procedures

The study visit schedule for participants is outlined in Table 2 and provides a description of the study procedures and investigations planned for each visit.

For peer review only

Table 2. Description of study visits and investigations in Teen TB

Time point	Activity	Tuberculosis disease	Tuberculosis exposure
		n=50	n=50
Baseline	Consent and recruitment	●	●
	Complete CRFs	●	●
	Portable spirometry	●	●
	Oscillometry	●	●
	Venepuncture	●	●
	Chest x-ray	●	●
	6-minute walk test	●	●
	St. George's Respiratory Questionnaire	●	●
	Full laboratory lung function		●
2 weeks	Complete CRFs	●	
	Ultrasound	●	
	Portable spirometry	●	
	Oscillometry	●	
	^{18}F-FDG PET-CT	●	
	Psychosocial questionnaire	●	
2 months	Complete CRFs	●	
	Venepuncture	●	
	Chest x-ray	●	
	Ultrasound	●	
	Full laboratory lung function	●	
	Portable spirometry	●	
	Oscillometry	●	
	6-minute walk test	●	
	St. George's Respiratory Questionnaire	●	
12 months	Complete CRFs	●	
	Oscillometry	●	
	Full laboratory lung function	●	
	Chest x-ray	●	
	6-minute walk test	●	
	St. George's Respiratory Questionnaire	●	

CRF: case report form; ^{18}F-FDG PET-CT: fluorine-18 fluorodeoxyglucose positron emission tomography combined with (x-ray) computed tomography.

only

Clinical data collection

At each study visit we will collect a full clinical TB history, comprehensive clinical data and socioeconomic data from all participants. A clinical examination, including a cardiorespiratory and abdominal exam, will take place at each visit with Tanner staging done at recruitment. A baseline weight and, in those with TB disease, a follow up weight at 2 weeks, 2 months and 12 months will be recorded. Programmatic and laboratory data (including microbiology) will be collected from the treating clinic and from the National Health Laboratory Service (NHLS).

Blood sample collection

At baseline, all adolescents will be offered an HIV test. If they have not had a test in the last 12 months, blood will be drawn to perform an HIV ELISA. If their HIV status is available from a recent result (from the past 12 months) then this result will be used. Any participant with a new HIV infection diagnosed during this study will be counselled appropriately by a trained member of the research team and referred to routine services for evaluation and initiation of anti-retroviral treatment. Participants with TB disease and TB-exposed participants will also have blood collected at baseline for CMV quantitative IgG, RNA sequencing, hormone profiling (including dehydroepiandrosterone sulfate, leptin, testosterone, oestradiol and progesterone levels) and for a panel of inflammatory cytokines relevant to TB biology, selected from previous work by the study team and from the literature. In TB-exposed individuals, blood will be taken for an interferon-gamma release assay (IGRA). Participants with TB disease will undergo an additional blood draw at two months and samples will be stored for later analysis.

Imaging

All adolescents with TB disease will undergo chest radiography (postero-anterior and lateral) as part of the standard routine care at baseline and will also undergo further chest x-rays (CXRs) at 2 months and 12 months. Those exposed to TB will have a baseline CXR only. Individuals with TB disease will undergo ultrasound at two weeks and two months, performed by trained medical personnel and using standard operating procedures. Ultrasound examination will include an evaluation of the chest, the abdomen and the mediastinum (through the suprasternal notch) and will be performed using two portable ultrasound machines – the Butterfly IQ and the Mindray DP 10. Two weeks into treatment, ^{18}F-FDG PET-CT imaging will be performed for all adolescents with TB disease at the NuMeRI Node for Infection Imaging of Stellenbosch University. This will be carried out using a Vereos Digital PET 64-channel CT scanner (Philips Medical Systems) with a dose-sparing acquisition protocol. Participants will be fasted for 4-6 hours prior to the administration of FDG, which will be administered via an intravenous cannula, using universal precautions and aseptic techniques.

For peer review only

Respiratory function assessment

Forced Expiratory Volume in 1 second (FEV1), Forced Vital Capacity (FVC) and the FEV1/FVC ratio will be carried out using a hand-held NDD EasyOne PC (Switzerland) spirometer at baseline in all adolescents and again at 2 weeks and 2 months in those with TB disease. A minimum of 3 trials up to a maximum of 8 attempts will be carried out per participant and at least 3 acceptable, reproducible best quality curves will be selected. The best value of 3 will be reported. Four hundred micrograms of salbutamol will be administered via spacer and testing will be repeated after 15 minutes to assess reversibility. Criteria for acceptability and repeatability of the spirometry curves will be based on European Respiratory Society (ERS)/American Thoracic Society (ATS). Global Lung Initiative (GLI) reference ranges will be used using the category “other” to reflect the South African population.[24,25]

The portable THORASYS® tremoFlo® C-100 Airwave Oscillometry System™ (AOS) will be used for additional assessment of pulmonary function. The TremoFlo is a tidal breathing technique that measures airway resistance and reactance and allows for detailed insights into small airway disease. All participants will have oscillometry at baseline, while those with TB disease will have this repeated at 2 weeks, 2 months and 12 months. Oscillometry will be done pre- and post-bronchodilation as described for spirometry.

In collaboration with the Tygerberg Lung Function Laboratory, all individuals with TB exposure but without disease will undergo a full lung function assessment two to four weeks after recruitment. For those with TB disease, the evaluation will take place at 2 months and 12 months post recruitment. This will include spirometry with pre- and post-bronchodilator measurement, diffusion capacity and plethysmography. These tests will be conducted according to ERS/ ATS guidelines and local hospital protocols.

All participants will undergo a six-minute walk test (6MWT). A trained technician will carry out the standard protocol as per the ATS guidelines.[26] The results will be compared with population-based standards.[27] Those with TB disease will undergo this again at 2 months and 12 months. Participants will also complete the St. George’s Respiratory Questionnaire (SGRQ) which assesses self-reported symptoms of breathlessness, and impact of this on activities and psychosocial function. Those with TB disease will have this repeated at 2 months and 12 months.

Psychosocial context data

All individuals with TB disease will complete a multi-component quantitative questionnaire about psychological and social impacts on their experiences (including stigma, psychological well-being, social support and similar) at week 2. These data will be analysed using common descriptive statistics.

For peer review only

We will also collect longitudinal in-depth qualitative data (3-4 interactions over the course of their participation in the project) with a nested, sub-sample of 20 adolescents, purposively sampled for diversity and richness, stratified 1:1 among adolescents with DS-TB and MDR-TB. The life course experiences of this sub-group will be characterised, including familial/peer social contexts, experiences of TB disease including disability, sexual experiences and time of sexual debut, HIV, smoking, substance abuse, violence, processes of peer support and socialisation, impact on education, episodic and chronic threats to mental well-being, accessing health services, TB treatment adherence and quality of life. We will use ATLAS.ti to conduct thematic and case descriptive analyses.

Statistical Analysis

Power and sample size

While multiple different analyses are planned for the different components of the study (clinical, immunology, radiology, social science, virology) we calculated the sample size based on ability to detect differences in lung function. Even within this discipline, several lung function parameters will be measured during this study, but sample size calculations were performed based on assumptions related to spirometry. A reduced FVC, compared to age and height reference standards, indicates restrictive lung disease, whereas a reduced FEV1 and FEV1/FVC ratio, compared to reference standards, indicate obstructive lung disease. Both patterns of lung disease have been seen in adults following pulmonary TB. To demonstrate a difference between healthy individuals in our context (assumed to have a FEV1/FVC ratio of 100%; Standard Deviation: 15%), and adolescents with pulmonary TB disease (assumed to have a ratio of 90%), with 90% power and an alpha of 0.05 we would need 47 adolescents with pulmonary TB and 47 healthy controls. Therefore, we plan to recruit 50 cases and 50 controls given the uncertainty in these estimates and to allow for any lost data or incomplete evaluations.

Assessment of study endpoints/ primary objectives

Descriptive analysis will be used to characterise the study populations, to compare the TB case and TB-exposed groups and aid in identifying differences between groups with respect to potential confounders. Categorical variables will be expressed as percentages and continuous variables will be expressed as mean ± one standard deviation (SD) or median with interquartile range (IQR), depending on the distribution of the data.

In those with TB disease, we will categorise disease on CXR as severe and non-severe, using established criteria[28] and will compare mean z-score of FVC, FEV1 and FEV1/FVC ratio at 2 months and 12 months in those with severe and non-severe disease using a paired sample T-test. We will also evaluate the relationship between severe and non-severe disease on CXR with other measures of functional tests (oscillometry, diffusion capacity, plethysmography, 6MWT and SGRQ). We will perform similar analyses

For peer review only

evaluating the ability of ultrasound, CT and ^{18}F-FDG PET-CT to predict different measures of lung function at 2 months and 12 months. We will compare the lung function of adolescents with TB disease and those with TB exposure to determine the impact of TB on lung health. From a radiology perspective, we will evaluate the ability of CXR and lung ultrasound to identify findings as seen on CT chest (considered as the imaging 'gold standard' for this analysis).

Further analyses will be performed to explore the impact of pubertal hormones on inflammatory cytokines and how the inflammatory cytokines of adolescents with TB disease and TB exposure differ. Immune responses to viral co-infections in participants with TB disease and those with TB exposure will also be compared. Analyses will be performed using STATA (version 17 STATA Corp., College Station, TX, USA).

Data Management and Confidentiality

At recruitment, each individual will be allocated a unique study number. Personal identifier details will be recorded (e.g., name, address and telephone number) but these will thereafter be kept securely and will remain separate from any clinical/epidemiological data, laboratory samples or data resulting from laboratory analysis. These research data will only be linked to the study number. All qualitative data will be audio recorded and transcripts of these recordings anonymised prior to presentation for any audiences outside of the study team. All data (clinical, epidemiological psychosocial and qualitative) will be stored on a secure database at DTTC.

Patient and Public Involvement

The development of the protocol, the ethics submission and the strategy for implementation of the study were undertaken in close collaboration with the Desmond Tutu TB Centre Community Advisory Board (CAB). The CAB assists with building and fostering partnerships between researchers and local study communities impacted by TB. The CAB will provide feedback to the research team regarding local issues at clinical research sites and will inform the team of any community concerns that can affect the conduct and implementation of study procedures.

During participant follow-up visits, feedback from adolescents and their caregivers will be used to assess the perceived burden of study interventions and to identify any aspects of their involvement that require improvement or adaptation.

ETHICS AND DISSEMINATION

The study protocol was approved by the Stellenbosch University Health Research Ethics Committee on 25 February 2020 (N19/10/148). The study will be conducted according to South African and

For peer review only

internationally accepted ethical standards, including the Declaration of Helsinki and the South African Good Clinical Practice (GCP) guidelines. For recruitment, the parents/legal guardians/participants will be required to provide written informed consent in their first language. Adolescents and families will be informed that they are under no obligation to enter the study and that they can withdraw at any time during the study, without having to give a reason.

The study findings will be disseminated through peer-reviewed publications, academic conferences and formal presentations to health professionals and practitioners. Findings will also be communicated to study participants and their caregivers.

PROJECT STATUS

Recruitment to the study started in November 2020. Follow-up of participants is still ongoing, and the final visit of the last participant with TB disease is planned to be in July 2022. The current protocol is version 1.0

DISCUSSION

Teen TB will provide much needed preliminary data in a research field in which data are almost entirely lacking. It will permit the design of a larger study to evaluate the relationship between baseline imaging and lung function with residual respiratory morbidity at the end of treatment and follow up, and potentially the evaluation of immunomodulators to reduce inflammatory damage caused by excessive host response.

In addition, increased understanding of the psychosocial impact of DS- and MDR-TB on adolescents would allow us to better design adolescent TB services that are more receptive to the needs to young people and more adolescent-friendly. This could lead to the development of interventional studies that evaluate how different models of adolescent TB care impact on the experiences and outcomes of young people with TB.

Finally, an improved understanding of TB biology in adolescents, and in particular, the impact of pubertal hormones and co-infections on the human immune response to *Mtb* will permit the evaluation of interventions that may either reduce the risk of TB disease progression following infection or improved clearance of *Mtb* once diagnosed with TB disease. It may also give insight into the design of novel vaccine strategies that are tailored to the immune milieu of adolescents of different genders and at different stages through puberty.

For peer review only

AUTHORS' CONTRIBUTIONS

The study concept and design were conceived by JAS. JAS is also the principal investigator for the Teen TB study. MvdZ, GH, MP, AD, SMJ, GvZ, BA, EW, DM, HSS and ACH provided additional methodological and/or clinical advice. KZ, GdB, KM, SJ, RS, CH and SG-R will conduct screening and data collection. DW assisted with psychosocial questionnaire development. Analyses will be performed by VJ, LK, CH and SM. JS prepared the first draft of the manuscript and will assist with data analysis. All authors provided edits and critiqued the manuscript.

FUNDING STATEMENT

This work was supported by The Global Challenges Research Fund. JAS is supported by a Clinician Scientist Fellowship jointly funded by the UK Medical Research Council (MRC) and the UK Department for International Development (DFID) under the MRC/DFID Concordat agreement (MR/R007942/1).

COMPETING INTERESTS

None declared

REFERENCES

1. Patton GC, Sawyer SM, Santelli JS, Ross DA, Afifi R, Allen NB, et al. Our future: a Lancet commission on adolescent health and wellbeing. Lancet. 2016 Jun;387(10036):2423–78.
2. Azzopardi PS, Hearps SJC, Francis KL, Kennedy EC, Mokdad AH, Kassebaum NJ, et al. Progress in adolescent health and wellbeing: tracking 12 headline indicators for 195 countries and territories, 1990–2016. Lancet. 2019 Mar;393(10176):1101–18.
3. Snow KJ, Sismanidis C, Denholm J, Sawyer SM, Graham SM. The incidence of tuberculosis among adolescents and young adults: a global estimate. Eur Respir J. 2018 Feb 21;51(2):1702352.
4. Seddon JA, Chiang SS, Esmail H, Coussens AK. The Wonder Years: What Can Primary School Children Teach Us About Immunity to Mycobacterium tuberculosis? Front Immunol. 2018 Dec 13;9.
5. Patterson B, Morrow CD, Kohls D, Deignan C, Ginsburg S, Wood R. Mapping sites of high TB transmission risk: Integrating the shared air and social behaviour of TB cases and adolescents in a South African township. Sci Total Environ. 2017 Apr;583:97–103.
6. Margarit A, Simó S, Rozas L, Deyà-Martínez À, Barrabeig I, Gené A, et al. [Adolescent tuberculosis; a challenge and opportunity to prevent community transmission]. An Pediatr (Barc). 2017 Mar;86(3):110–4.
7. Cruz AT, Hwang KM, Birnbaum GD, Starke JR. Adolescents With Tuberculosis. Pediatr Infect Dis J. 2013 Sep;32(9):937–41.
8. de Pontual L, Balu L, Ovetchkine P, Maury-Tisseron B, Lachassinne E, Cruaud P, et al.

For peer review only

Tuberculosis in Adolescents: A French retrospective study of 52 cases. Pediatr Infect Dis J. 2006 Oct;25(10):930–2.

9. Ssengooba W, Kateete DP, Wajja A, Bugumirwa E, Mboowa G, Namaganda C, et al. An Early Morning Sputum Sample Is Necessary for the Diagnosis of Pulmonary Tuberculosis, Even with More Sensitive Techniques: A Prospective Cohort Study among Adolescent TB-Suspects in Uganda. Tuberc Res Treat. 2012;2012:1–6.

10. Stevens H, Ximenes RA, Dantas OM, Rodrigues LC. Risk factors for tuberculosis in older children and adolescents: a matched case–control study in Recife, Brazil. Emerg Themes Epidemiol [Internet]. 2014 Dec 30;11(1):20. Available from: https://ete-online.biomedcentral.com/articles/10.1186/s12982-014-0020-5

11. Enane LA, Lowenthal ED, Arscott-Mills T, Matlhare M, Smallcomb LS, Kgwaadira B, et al. Loss to follow-up among adolescents with tuberculosis in Gaborone, Botswana. Int J Tuberc Lung Dis. 2016 Oct 1;20(10):1320–5.

12. Snow K, Hesseling AC, Naidoo P, Graham SM, Denholm J, du Preez K. Tuberculosis in adolescents and young adults: epidemiology and treatment outcomes in the Western Cape. Int J Tuberc Lung Dis. 2017 Jun 1;21(6):651–7.

13. Isaakidis P, Paryani R, Khan S, Mansoor H, Manglani M, Valiyakath A, et al. Poor Outcomes in a Cohort of HIV-Infected Adolescents Undergoing Treatment for Multidrug-Resistant Tuberculosis in Mumbai, India. Wilkinson RJ, editor. PLoS One. 2013 Jul 19;8(7):e68869.

14. Ravimohan S, Kornfeld H, Weissman D, Bisson GP. Tuberculosis and lung damage: from epidemiology to pathophysiology. Eur Respir Rev. 2018 Mar 31;27(147):170077.

15. Allwood BW, Byrne A, Meghji J, Rachow A, van der Zalm MM, Schoch OD. Post-Tuberculosis Lung Disease: Clinical Review of an Under-Recognised Global Challenge. Respiration [Internet]. 2021;100(8):751–63. Available from: https://www.karger.com/Article/FullText/512531

16. Stocks J, Hislop A, Sonnappa S. Early lung development: lifelong effect on respiratory health and disease. Lancet Respir Med. 2013 Nov;1(9):728–42.

17. Meghji J, Mortimer K, Agusti A, Allwood BW, Asher I, Bateman ED, et al. Improving lung health in low-income and middle-income countries: from challenges to solutions. Lancet (London, England) [Internet]. 2021;397(10277):928–40. Available from: http://www.ncbi.nlm.nih.gov/pubmed/33631128

18. Pasipanodya JG, McNabb SJ, Hilsenrath P, Bae S, Lykens K, Vecino E, et al. Pulmonary impairment after tuberculosis and its contribution to TB burden. BMC Public Health. 2010 Dec 19;10(1):259.

19. Chung K-P, Chen J-Y, Lee C-H, Wu H-D, Wang J-Y, Lee L-N, et al. Trends and predictors of changes in pulmonary function after treatment for pulmonary tuberculosis. Clinics. 2011;66(4):549–56.

20. Nagata JM, Hathi S, Ferguson BJ, Hindin MJ, Yoshida S, Ross DA. Research priorities for adolescent health in low- and middle-income countries: A mixed-methods synthesis of two separate

For peer review only

exercises. J Glob Health. 2018 Jun;8(1).

21. World Health Organization. Roadmap towards ending TB in children and adolescents. [Internet]. Geneva; 2018. Available from: http://apps.who.int/iris/bitstream/handle/10665/274374/9789241514668-eng.pdf?ua=1
22. Kanabus A. TB Statistics South Africa - Incidence, prevalence [Internet]. 2015. Available from: https://tbfacts.org/tb-statistics-south-africa/
23. National Department of Health. The 2015 National Antenatal Sentinel HIV & Syphilis Survey, South Africa. [Internet]. 2015. Available from: https://www.auruminstitute.org/chaps/chs/category/12-chs-country-statistics?download=187:2015-national-antenatal-hiv-prevalence-survey-final-23oct17
24. Quanjer PH, Stanojevic S, Cole TJ, Baur X, Hall GL, Culver BH, et al. Multi-ethnic reference values for spirometry for the 3–95-yr age range: the global lung function 2012 equations. Eur Respir J [Internet]. 2012 Dec;40(6):1324–43. Available from: http://erj.ersjournals.com/lookup/doi/10.1183/09031936.00080312
25. Smith S-J, Gray DM, MacGinty RP, Hall GL, Stanojevic S, Mphahlele R, et al. Choosing the Better Global Lung Initiative 2012 Equation in South African Population Groups. Am J Respir Crit Care Med [Internet]. 2020;202(12):1724–7. Available from: http://www.ncbi.nlm.nih.gov/pubmed/32757942
26. ATS Statement. Am J Respir Crit Care Med. 2002 Jul;166(1):111–7.
27. Mylius CF, Paap D, Takken T. Reference value for the 6-minute walk test in children and adolescents: a systematic review. Expert Rev Respir Med. 2016 Dec 21;10(12):1335–52.
28. Wiseman CA, Gie RP, Starke JR, Schaaf HS, Donald PR, Cotton MF, et al. A Proposed Comprehensive Classification of Tuberculosis Disease Severity in Children. Pediatr Infect Dis J [Internet]. 2012 Apr;31(4):347–52. Available from: https://journals.lww.com/00006454-201204000-00006

For peer review only

For peer review only

BMJ Open

Understanding the biology, morbidity and social contexts of adolescent tuberculosis: a prospective observational cohort study protocol (Teen TB)

Journal:	*BMJ Open*
Manuscript ID	bmjopen-2022-062979.R1
Article Type:	Protocol
Date Submitted by the Author:	28-Sep-2022

Complete List of Authors:	Swanepoel, Jeremi; Desmond Tutu TB Centre, Department of Paediatrics and Child Health, Faculty of Medicine and Health Sciences, Stellenbosch University; London School of Hygiene & Tropical Medicine<br>Zimri, Klassina; Desmond Tutu TB Centre, Department of Paediatrics and Child Health, Faculty of Medicine and Health Sciences, Stellenbosch University<br>van der Zalm, Marieke; Desmond Tutu TB Centre, Department of Paediatrics and Child Health, Faculty of Medicine and Health Sciences, Stellenbosch University<br>Hoddinott, Graeme; Desmond Tutu TB Centre, Department of Paediatrics and Child Health, Faculty of Medicine and Health Sciences, Stellenbosch University<br>Palmer, Megan ; Desmond Tutu TB Centre, Department of Paediatrics and Child Health, Faculty of Medicine and Health Sciences, Stellenbosch University<br>Doruyter, Alex; NuMeRI Node for Infection Imaging, Central Analytical Facilities, Stellenbosch University; Division of Nuclear Medicine, Department of Medical Imaging and Clinical Oncology, Stellenbosch University<br>De Beer, Gezila; Desmond Tutu TB Centre, Department of Paediatrics and Child Health, Faculty of Medicine and Health Sciences, Stellenbosch University<br>Kleynhans, Leanie; DSI-NRF Centre of Excellence, South African Medical Research Council Centre for Tuberculosis Research, Division of Molecular Biology and Human Genetics, Department of Biomedical Sciences, Faculty of Medicine and Health Sciences, Stellenbosch University<br>Johnson, Sarah; Imperial College London, Department of Infectious Diseases<br>Jongen, Vita; Public Health Service of Amsterdam, Department of Infectious Diseases<br>Wademan, Dillon; Desmond Tutu TB Centre, Department of Paediatrics and Child Health, Faculty of Medicine and Health Sciences, Stellenbosch University<br>Mcimeli, Khanyisa; Desmond Tutu TB Centre, Department of Paediatrics and Child Health, Faculty of Medicine and Health Sciences, Stellenbosch University<br>Jacobs, Stephanie; Desmond Tutu TB Centre, Department of Paediatrics and Child Health, Faculty of Medicine and Health Sciences, Stellenbosch University

	Swanepoel, Ruan; Tygerberg Hospital, Division of Pulmonology, Department of Internal Medicine<br>U Van Zyl, Gert; Stellenbosch University Faculty of Medicine and Health Sciences, Division of Medical Virology, Department of Pathology<br>Allwood, Brian; Stellenbosch University Faculty of Medicine and Health Sciences, Division of Pulmonology, Department of Medicine<br>Malherbe, Stephanus; DSI-NRF Centre of Excellence, South African Medical Research Council Centre for Tuberculosis Research, Division of Molecular Biology and Human Genetics, Department of Biomedical Sciences, Faculty of Medicine and Health Sciences, Stellenbosch University<br>Heuvelings, Charlotte; Desmond Tutu TB Centre, Department of Paediatrics and Child Health, Faculty of Medicine and Health Sciences, Stellenbosch University<br>Griffith-Richards, Stephanie; Division of Radiodiagnosis, Department of Medical Imaging and Clinical Oncology, Faculty of Medicine and Health Sciences, Stellenbosch University<br>Whittaker, Elizabeth ; Imperial College London, Department of Infectious Diseases<br>Moore, Dave; London School of Hygiene and Tropical Medicine Faculty of Infectious and Tropical Diseases<br>Schaaf, Simon; Desmond Tutu TB Centre, Department of Paediatrics and Child Health, Faculty of Medicine and Health Sciences, Stellenbosch University<br>Hesseling, A C; Desmond Tutu TB Centre, Department of Paediatrics and Child Health, Faculty of Medicine and Health Sciences, Stellenbosch University<br>Seddon, James; Desmond Tutu TB Centre, Department of Paediatrics and Child Health, Faculty of Medicine and Health Sciences, Stellenbosch University; Imperial College London, Department of Infectious Diseases
**Primary Subject Heading**:	Paediatrics
Secondary Subject Heading:	Infectious diseases, Respiratory medicine
Keywords:	Tuberculosis < INFECTIOUS DISEASES, Paediatric infectious disease & immunisation < PAEDIATRICS, Paediatric thoracic medicine < PAEDIATRICS

SCHOLARONE™
Manuscripts

TITLE PAGE

Manuscript Title

Understanding the biology, morbidity and social contexts of adolescent tuberculosis: a prospective observational cohort study protocol (Teen TB)

Authors

Jeremi Swanepoel[1,2]
Klassina Zimri[1]
Marieke M. van der Zalm[1]
Graeme Hoddinott[1]
Megan Palmer[1]
Alex Doruyter[3,4]
Gezila de Beer[1]
Léanie Kleynhans[5]
Sarah M. Johnson[6]
Vita Jongen[7]
Dillon Wademan[1]
Khanyisa Mcimeli[1]
Stephanie Jacobs[1]
Ruan Swanepoel[8]
Gert van Zyl[9]
Brian Allwood[10]
Stephanus Malherbe[5]
Charlotte Heuvelings[1]
Stephanie Griffith-Richards[11]
Elizabeth Whittaker[6]
David A. J. Moore[2]
H Simon Schaaf[1]
Anneke C. Hesseling[1]
James A. Seddon[1,6]

For peer review only

Affiliations

[1]Desmond Tutu TB Centre, Department of Paediatrics and Child Health, Faculty of Medicine and Health Sciences, Stellenbosch University, South Africa

[2]TB Centre, London School of Hygiene and Tropical Medicine, London, United Kingdom

[3]NuMeRI Node for Infection Imaging, Central Analytical Facilities, Stellenbosch University

[4]Division of Nuclear Medicine, Stellenbosch University

[5]DSI-NRF Centre of Excellence for Biomedical Tuberculosis Research, South African Medical Research Council Centre for Tuberculosis Research, Division of Molecular Biology and Human Genetics, Department of Biomedical Sciences, Faculty of Medicine and Health Sciences, Stellenbosch University, Cape Town, South Africa

[6]Department of Infectious Diseases, Imperial College London, London, United Kingdom

[7]Department of Infectious Diseases, Public Health Service Amsterdam, Amsterdam, The Netherlands

[8]Division of Pulmonology, Department of Internal Medicine, Tygerberg Academic Hospital

[9]Division of Medical Virology, Stellenbosch University and NHLS Tygerberg, Cape Town, South Africa

[10]Division of Pulmonology, Department of Medicine, Faculty of Medicine and Health Sciences, Stellenbosch University, South Africa

[11]Division of Radiodiagnosis, Department of Medical Imaging and Clinical Oncology, Faculty of Medicine and Health Sciences, Stellenbosch University

Keywords

Tuberculosis

Adolescents

Lung Function

Puberty

Stigma

Word Count

Abstract: 261

Main text: 3409

For peer review only

Corresponding Author

James Seddon

Desmond Tutu TB Centre

Department of Paediatrics and Child Health

Faculty of Medicine and Health Sciences

Stellenbosch University

South Africa

jseddon@sun.ac.za

For peer review only

ABSTRACT

Introduction:

A considerable burden of the tuberculosis (TB) epidemic is found in adolescents. The reasons for increased susceptibility to TB infection and higher incidence of TB disease in adolescence, compared to the 5 to 10-year-old age group, are incompletely understood. Despite the pressing clinical and public health need to better understand and address adolescent TB, research in this field remains limited.

Methods and analysis:

Teen TB is an ongoing prospective observational cohort study that aims to better understand the biology, morbidity and social context of adolescent TB. The study plans to recruit 50 adolescents (10-19-years-old) with newly diagnosed microbiologically confirmed pulmonary TB disease and 50 TB-exposed controls without evidence of TB disease in Cape Town, South Africa, which is highly endemic for TB. At baseline, cases and controls will undergo a detailed clinical evaluation, chest imaging, respiratory function assessments and blood collection for viral co-infections, inflammatory cytokines and pubertal hormone testing. At 2 weeks, 2 months and 12 months, TB disease cases will undergo further chest imaging and additional lung function testing to explore the patterns of respiratory abnormalities. At week 2, cases will complete a multi-component quantitative questionnaire about psychological and social impacts on their experiences and longitudinal, in-depth qualitative data will be collected from a nested sub-sample of 20 cases and their families.

Ethics and dissemination:

The study protocol has received ethical approval from the Stellenbosch University Health Research Ethics Committee (N19/10/148). The study findings will be disseminated through peer-reviewed publications, academic conferences and formal presentations to health professionals. Results will also be made available to participants and caregivers.

For peer review only

STRENGHTS AND LIMITATIONS OF THIS STUDY

- A key strength of this study is the use of multiple imaging methods and respiratory function testing modalities to delineate the patterns of structural and functional respiratory abnormalities in adolescent pulmonary TB.
- Our longitudinal study design will permit us to characterise the sociological and psychological impact of both drug-susceptible and multidrug-resistant TB on adolescents over time.
- The comprehensive measurement of pubertal hormones, viral co-infections and inflammatory cytokines relevant to TB biology will contribute to identifying host factors that impact the human immune response to *Mycobacterium tuberculosis.*
- The small sample size and susceptibility to loss to follow up are limitations of this study.

For peer review only

INTRODUCTION

There are an estimated 1.6 billion adolescents (aged 10 to 19 years) living in the world today, with 90% in low- and middle-income countries (LMICs).[1,2] Modelling studies suggest that 750,000 adolescents develop tuberculosis (TB) disease each year globally,[3] yet adolescent TB remains a neglected field of research. Adolescents have unique biological and social circumstances that differ from both younger children and adults. These include hormonal and immunological pubertal changes, increasing autonomy, extensive socialisation and risk-taking behaviour. These unique features have been poorly studied in resource-limited settings, particularly in relation to their impact on health.

For reasons incompletely understood, the risk of progression from TB infection to TB disease rises in adolescence.[4] This may be related to the impact of puberty on the immunological response to *Mycobacterium tuberculosis (Mtb)*,[4] or due to co-infections, nutrition and substance abuse (alcohol, smoking, illicit drugs) impacting on TB pathogenesis, as well as changes in social life impacting on exposure risks. Adolescents tend to develop infectious, cavitary forms of disease,[5–9] thus having a greater impact than younger children on TB transmission. Furthermore, there is extensive social mixing,[3,5] late presentation of TB disease,[10] poor adherence to treatment and follow up and high rates of loss to follow up.[11] These may all have a substantial impact on TB within the adolescent population and on propagation of the epidemic.[6] Outcomes for adolescents with TB are worse than most other population groups.[12] Treatment challenges are more complex when multidrug-resistant TB (MDR-TB; defined as disease caused by *Mtb* resistant to at least rifampicin and isoniazid) is present or when there is HIV co-infection.[13] Finally, the impact of TB on respiratory morbidity in adolescents is almost entirely unexplored. Emerging evidence in adults demonstrates that more than half of individuals develop clinically relevant respiratory morbidity post pulmonary TB,[14,15] and there is evolving appreciation that respiratory insults in childhood and adolescence have lifelong consequences.[16,17] Any functional impairment may have an impact on their quality of life, their future educational and employment potential, as well as their long-term health.[18,19] This illustrates the importance of exploring both pulmonary function and psychosocial aspects of adolescent TB in research.

TB has been identified as a key research priority for adolescent health in LMICs,[20] and adolescent TB is a crucial component of the revised World Health Organization (WHO) Roadmap for ending TB.[21] We therefore set out to conduct a study to examine the biology, morbidity and psychosocial contexts of adolescent TB, named Teen TB. This study will provide a vehicle to explore how these different elements of adolescent TB interact. Teen TB will also allow an unparalleled opportunity to explore the patterns of abnormalities found on chest radiography, ultrasound imaging and ^{18}F-fluorodeoxyglucose (FDG) Positron Emission Tomography (PET) combined with chest radiography and chest computed

tomography (CT) in adolescents with TB. In addition, it will enable these findings to be correlated with the extent of respiratory deficit, social contexts and biological markers. Understanding this will inform our understanding of adolescent TB treatment, adherence and transmission and will have implications for future interventional studies.

Study objectives

The Teen TB study has three main objectives. The first is to evaluate the relationship between baseline lung imaging (advanced and point-of-care) and respiratory function in adolescents with TB disease. We aim to test the hypothesis that imaging at the time of diagnosis can predict future lung function. The second objective is to explore the psychosocial experience of adolescents affected by drug-susceptible (DS) and MDR-TB and evaluate if the diagnosis of TB is associated with experiences of stigma. The third objective is to explore how pubertal hormones and viral co-infections (notably cytomegalovirus [CMV]) influence the immune response to *Mtb*. We hypothesise that as adolescents pass through puberty, they generate an increasingly pronounced myeloid inflammatory response to *Mtb*.

METHODS AND ANALYSIS

Study design and setting

Teen TB is a prospective observational cohort study which plans to recruit 50 adolescents (10 to 19 years) with newly diagnosed microbiologically confirmed TB disease (including both MDR-TB and DS-TB) in Cape Town, Western Cape, South Africa and follow them over a 12-month period. We will also recruit 50 TB-exposed controls (exposed to both DS-TB and MDR-TB). South Africa's Western Cape province is highly endemic for TB and previous studies have shown a substantial burden of TB among adolescents in this setting.[12] The overall TB incidence rate in the Western Cape was 681/100,000 in 2015 and, in a study from the same province, the incidence of TB in adolescents was found to be 450/100,000.[22,23] In 2020-21, the estimated HIV incidence rates for adults aged 15-49 years in the Western Cape was 0.51%.[24] This high burden setting is an ideal place to carry out adolescent TB studies to meet research gaps.

Study participants

Any adolescent with newly diagnosed pulmonary TB bacteriologically confirmed on sputum (Xpert- or culture-positive), with or without HIV co-infection and who are within the first 14 days of treatment, are eligible to participate in this study. Any adolescent exposed in their household to a case of infectious pulmonary TB and who has no symptoms of TB, will be considered for the TB-exposed control group. Those with TB disease will be excluded from the study if they have extrapulmonary TB without evidence of pulmonary TB. TB-exposed controls will be excluded if they have previously recovered from TB disease or if asymptomatic TB disease is suspected and/or diagnosed by baseline study

For peer review only

investigations. Those suspected of having asymptomatic TB disease will be considered for the TB disease group if they meet the inclusion criteria. Both participants with TB disease and TB exposure will be excluded if they have a severe illness or any condition causing the adolescent to be clinically unstable or requiring intensive care treatment, if pregnant or breastfeeding, if known to have diabetes mellitus and if HIV testing is declined with no recent (<12 months) HIV test result available (**Table 1**).

Table 1. Inclusion and exclusion criteria for Teen TB

	Inclusion	Exclusion
**Tuberculosis disease**	Any adolescent (10 to 19 years) who:<br>• Has a primary diagnosis of newly diagnosed pulmonary TB bacteriologically confirmed on sputum (Xpert- or culture-positive), with or without HIV coinfection<br>• Is within the first 14 days since diagnosis and thus 14 days of TB treatment	• Extrapulmonary TB without evidence of pulmonary TB<br>• Severe illness or any condition causing the adolescent to be clinically unstable or require intensive care treatment<br>• Pregnancy or breastfeeding (due to exposure to ionizing radiation)<br>• Diabetes mellitus (due to the potential confounding effects of hyperglycaemia and insulin levels on ^{18}F-FDG uptake on PET/CT)<br>• Participants declining HIV testing for whom a recent (<12 month) HIV test result is not available
**Tuberculosis exposure**	Any adolescent (10 to 19 years) who:<br>• Has been exposed in their household in the last 6 months to a case of infectious pulmonary TB<br>• Has no symptoms of TB disease	• Previous TB disease<br>• Asymptomatic TB disease<br>• Severe illness or any condition causing the adolescent to be clinically unstable or require intensive care treatment<br>• Pregnancy or breastfeeding (to avoid any confounding in comparison with cases)<br>• Diabetes mellitus (to avoid any confounding in comparison with cases)<br>• Participants declining HIV testing for whom a recent (<12 month) HIV test result is not available

TB: tuberculosis; ^{18}F-FDG: 18F-fluorodeoxyglucose; PET/CT: positron emission tomography and computed tomography; HIV: Human Immunodeficiency Virus

Recruitment Process

DS-TB recruitment will be carried out in collaboration with clinical staff in health facilities in the Tygerberg, Mitchell's Plain and Khayelitsha sub-districts of the City of Cape Town Metropole. As adolescents present to health facilities in these sub-districts with TB symptoms, we will approach them and sensitise them to the study. If they are diagnosed with DS-TB and meet the eligibility criteria, we will approach them prior to starting TB treatment to see if they would be willing to participate. If willing to participate, informed written consent will be obtained from parents/legal guardians for those under 18 years with additional assent from the adolescent, whereas 18- and 19-year-olds can provide consent for themselves. Initial consultations will take place at their homes, local clinics or at Desmond Tutu TB Centre (DTTC) field sites, as determined to be most convenient to the participant and their family. Given the necessity for all participants to undertake lung function testing at Tygerberg Hospital and to undergo ^{18}F-FDG PET-CT imaging for those with TB disease, the follow-up visit will be at the iKamva Unit on the Tygerberg Campus of Stellenbosch University (SU).

MDR-TB recruitment will be carried out using the established TB-CHAMP platform to identify adolescents aged 10-19 years in the Metropolitan area who are newly diagnosed with MDR-TB disease. TB-CHAMP is an ongoing MDR-TB prevention trial that randomises children who have been exposed to MDR-TB in their household to levofloxacin or placebo, with three South African sites, and in collaboration with the MRC Clinical Trials Unit at University College London. The TB-CHAMP trial team identifies every MDR-TB patient in the Cape Town Metropolitan area through laboratory and clinical sources. This is an established platform by which adolescent MDR-TB cases can be identified. We will then, using our established links with routine services, and in collaboration with their healthcare providers, contact these adolescents, following sensitisation to the study by local clinics, ideally prior to starting MDR-TB treatment, and invite them to join the study.

TB-exposed controls are recruited from the same clinics where individuals with TB disease are recruited, and the contacts of the enrolled cases will also be eligible for inclusion. TB-exposed controls must have been exposed to an infectious DS-TB case within the last 6 months. We will again use the TB-CHAMP platform to aid in identifying adolescents who have been exposed to an infectious pulmonary MDR-TB case in the household within the last 6 months.

Study Procedures

The study visit schedule for participants is outlined in Table 2 and provides a description of the study procedures and investigations planned for each visit.

For peer review only

Table 2. Description of study visits and investigations in Teen TB

Time point	Activity	Tuberculosis disease	Tuberculosis exposure
		n=50	n=50
Baseline	Consent and recruitment	●	●
	Complete CRFs	●	●
	Portable spirometry	●	●
	Oscillometry	●	●
	Venepuncture	●	●
	Chest x-ray	●	●
	6-minute walk test	●	●
	St. George's Respiratory Questionnaire	●	●
	Full laboratory lung function		●
2 weeks	Complete CRFs	●	
	Ultrasound	●	
	Portable spirometry	●	
	Oscillometry	●	
	^{18}F-FDG PET-CT	●	
	Psychosocial questionnaire	●	
2 months	Complete CRFs	●	
	Venepuncture	●	
	Chest x-ray	●	
	Ultrasound	●	
	Full laboratory lung function	●	
	Portable spirometry	●	
	Oscillometry	●	
	6-minute walk test	●	
	St. George's Respiratory Questionnaire	●	
12 months	Complete CRFs	●	
	Oscillometry	●	
	Full laboratory lung function	●	
	Chest x-ray	●	
	6-minute walk test	●	
	St. George's Respiratory Questionnaire	●	

CRF: case report form; ^{18}F-FDG PET-CT: fluorine-18 fluorodeoxyglucose positron emission tomography combined with (x-ray) computed tomography.

only

Clinical data collection

At each study visit we will collect a full clinical TB history, comprehensive clinical data and socioeconomic data from all participants. A clinical examination, including a cardiorespiratory and abdominal exam, will take place at each visit with Tanner staging done at recruitment. A baseline weight and, in those with TB disease, a follow up weight at 2 weeks, 2 months and 12 months will be recorded. Programmatic and laboratory data (including microbiology) will be collected from the treating clinic and from the National Health Laboratory Service (NHLS).

Blood sample collection

At baseline, all adolescents will be offered an HIV test. If they have not had a test in the last 12 months, blood will be drawn to perform an HIV ELISA. If their HIV status is available from a recent result (from the past 12 months) then this result will be used. Any participant with a new HIV infection diagnosed during this study will be counselled appropriately by a trained member of the research team and referred to routine services for evaluation and initiation of anti-retroviral treatment. Participants with TB disease and TB-exposed participants will also have blood collected at baseline for CMV quantitative IgG, RNA sequencing, hormone profiling (including dehydroepiandrosterone sulfate, leptin, testosterone, oestradiol and progesterone levels) and for a panel of inflammatory cytokines relevant to TB biology, selected from previous work by the study team and from the literature. In TB-exposed individuals, blood will be taken for an interferon-gamma release assay (IGRA). Participants with TB disease will undergo an additional blood draw at two months and samples will be stored for later analysis.

Imaging

All adolescents with TB disease will undergo chest radiography (postero-anterior and lateral) as part of the standard routine care at baseline and will also undergo further chest x-rays (CXRs) at 2 months and 12 months. Those exposed to TB will have a baseline CXR only. Individuals with TB disease will undergo ultrasound at two weeks and two months, performed by trained medical personnel and using standard operating procedures. Ultrasound examination will include an evaluation of the chest, the abdomen and the mediastinum (through the suprasternal notch) and will be performed using two portable ultrasound machines – the Butterfly IQ and the Mindray DP 10. Two weeks into treatment, ^{18}F-FDG PET-CT imaging will be performed for all adolescents with TB disease at the NuMeRI Node for Infection Imaging of Stellenbosch University. This will be carried out using a Vereos Digital PET 64-channel CT scanner (Philips Medical Systems) with a dose-sparing acquisition protocol. Participants will be fasted for 4-6 hours prior to the administration of FDG, which will be administered via an intravenous cannula, using universal precautions and aseptic techniques.

For peer review only

Respiratory function assessment

Forced Expiratory Volume in 1 second (FEV1), Forced Vital Capacity (FVC) and the FEV1/FVC ratio will be carried out using a hand-held NDD EasyOne PC (Switzerland) spirometer at baseline in all adolescents and again at 2 weeks and 2 months in those with TB disease. A minimum of 3 trials up to a maximum of 8 attempts will be carried out per participant and at least 3 acceptable, reproducible best quality curves will be selected. The best value of 3 will be reported. Four hundred micrograms of salbutamol will be administered via spacer and testing will be repeated after 15 minutes to assess reversibility. Criteria for acceptability and repeatability of the spirometry curves will be based on European Respiratory Society (ERS)/American Thoracic Society (ATS). Global Lung Initiative (GLI) reference ranges will be used using the category "other" to reflect the South African population.[25,26]

The portable THORASYS® tremoFlo® C-100 Airwave Oscillometry System™ (AOS) will be used for additional assessment of pulmonary function. The TremoFlo is a tidal breathing technique that measures airway resistance and reactance and allows for detailed insights into small airway disease. All participants will have oscillometry at baseline, while those with TB disease will have this repeated at 2 weeks, 2 months and 12 months. Oscillometry will be done pre- and post-bronchodilation as described for spirometry.

In collaboration with the Tygerberg Lung Function Laboratory, all individuals with TB exposure but without disease will undergo a full lung function assessment as soon after recruitment as possible. For those with TB disease, the evaluation will take place at 2 months and 12 months post recruitment. This will include spirometry with pre- and post-bronchodilator measurement, diffusion capacity and plethysmography. These tests will be conducted according to ERS/ ATS guidelines and local hospital protocols.

All participants will undergo a six-minute walk test (6MWT). A trained technician will carry out the standard protocol as per the ATS guidelines.[27] The results will be compared with population-based standards.[28] Those with TB disease will undergo this again at 2 months and 12 months. Participants will also complete the St. George's Respiratory Questionnaire (SGRQ) which assesses self-reported symptoms of breathlessness, and impact of this on activities and psychosocial function. Those with TB disease will have this repeated at 2 months and 12 months.

Psychosocial context data

All individuals with TB disease will complete a multi-component quantitative questionnaire (See supplementary file 1) about psychological and social impacts on their experiences (including stigma, psychological well-being, social support and similar) at week 2. These data will be analysed using

For peer review only

common descriptive statistics. We will also collect longitudinal in-depth qualitative data (3-4 interactions over the course of their participation in the project) with a nested, sub-sample of 20 adolescents, purposively sampled for diversity and richness, stratified 1:1 among adolescents with DS-TB and MDR-TB. The life course experiences of this sub-group will be characterised, including familial/peer social contexts, experiences of TB disease including disability, sexual experiences and time of sexual debut, HIV, smoking, substance abuse, violence, processes of peer support and socialisation, impact on education, episodic and chronic threats to mental well-being, accessing health services, TB treatment adherence and quality of life. We will use ATLAS.ti to conduct thematic and case descriptive analyses.

Statistical Analysis

Power and sample size

While multiple different analyses are planned for the different components of the study (clinical, immunology, radiology, social science, virology) we calculated the sample size based on ability to detect differences in lung function. Even within this discipline, several lung function parameters will be measured during this study, but sample size calculations were performed based on assumptions related to spirometry. A reduced FVC, compared to age and height reference standards, indicates restrictive lung disease, whereas a reduced FEV1 and FEV1/FVC ratio, compared to reference standards, indicate obstructive lung disease. Both patterns of lung disease have been seen in adults following pulmonary TB. To demonstrate a difference between healthy individuals in our context (assumed to have a FEV1/FVC ratio of 100%; Standard Deviation: 15%), and adolescents with pulmonary TB disease (assumed to have a ratio of 90%), with 90% power and an alpha of 0.05 we would need 47 adolescents with pulmonary TB and 47 healthy controls. Therefore, we plan to recruit 50 cases and 50 controls given the uncertainty in these estimates and to allow for any lost data or incomplete evaluations.

Assessment of study endpoints/ primary objectives

Descriptive analysis will be used to characterise the study populations, to compare the TB case and TB-exposed groups and aid in identifying differences between groups with respect to potential confounders. Categorical variables will be expressed as percentages and continuous variables will be expressed as mean ± one standard deviation (SD) or median with interquartile range (IQR), depending on the distribution of the data.

In those with TB disease, we will categorise disease on CXR as severe and non-severe, using established criteria[29] and will compare mean z-score of FVC, FEV1 and FEV1/FVC ratio at 2 months and 12 months in those with severe and non-severe disease using a paired sample T-test. We will also evaluate the relationship between severe and non-severe disease on CXR with other measures of functional tests

For peer review only

(oscillometry, diffusion capacity, plethysmography, 6MWT and SGRQ). We will perform similar analyses evaluating the ability of ultrasound, CT and ^{18}F-FDG PET-CT to predict different measures of lung function at 2 months and 12 months. We will compare the lung function of adolescents with TB disease and those with TB exposure using multivariable logistic regression to adjust for potential confounders and to determine the impact of TB on lung health. From a radiology perspective, we will evaluate the ability of CXR and lung ultrasound to identify findings as seen on CT chest (considered as the imaging 'gold standard' for this analysis) using either contingency tests or ROC (Receiver Operating Characteristic) analysis, where appropriate.

Further multivariable analyses will be performed to explore the impact of pubertal hormones on inflammatory cytokines and how the inflammatory cytokines of adolescents with TB disease and TB exposure differ. Immune responses to viral co-infections in participants with TB disease and those with TB exposure will also be compared using regression analysis. Analyses will be performed using STATA (version 17 STATA Corp., College Station, TX, USA).

Data Management and Confidentiality

At recruitment, each individual will be allocated a unique study number. Personal identifier details will be recorded (e.g., name, address and telephone number) but these will thereafter be kept securely and will remain separate from any clinical/epidemiological data, laboratory samples or data resulting from laboratory analysis. These research data will only be linked to the study number. All qualitative data will be audio recorded and transcripts of these recordings anonymised prior to presentation for any audiences outside of the study team. All data (clinical, epidemiological psychosocial and qualitative) will be stored on a secure database at DTTC.

Patient and Public Involvement

The development of the protocol, the ethics submission and the strategy for implementation of the study were undertaken in close collaboration with the Desmond Tutu TB Centre Community Advisory Board (CAB). The CAB assists with building and fostering partnerships between researchers and local study communities impacted by TB. The CAB will provide feedback to the research team regarding local issues at clinical research sites and will inform the team of any community concerns that can affect the conduct and implementation of study procedures.

During participant follow-up visits, feedback from adolescents and their caregivers will be used to assess the perceived burden of study interventions and to identify any aspects of their involvement that require improvement or adaptation.

For peer review only

ETHICS AND DISSEMINATION

The study protocol was approved by the Stellenbosch University Health Research Ethics Committee on 25 February 2020 (N19/10/148). The study will be conducted according to South African and internationally accepted ethical standards, including the Declaration of Helsinki and the South African Good Clinical Practice (GCP) guidelines. For recruitment, the parents/legal guardians/participants will be required to provide written informed consent in their first language. Adolescents and families will be informed that they are under no obligation to enter the study and that they can withdraw at any time during the study, without having to give a reason.

The study findings will be disseminated through peer-reviewed publications, academic conferences and formal presentations to health professionals and practitioners. Findings will also be communicated to study participants and their caregivers.

PROJECT STATUS

Recruitment to the study started in November 2020. Follow-up of participants is still ongoing, and the final visit of the last participant with TB disease is planned to be in July 2022. The current protocol is version 1.0

AUTHORS' CONTRIBUTIONS

The study concept and design were conceived by J Seddon. J Seddon is also the principal investigator for the Teen TB study. MvdZ, GH, MP, AD, S Johnson, GvZ, BA, EW, DM, SS and AH provided additional methodological and/or clinical advice. KZ, GdB, KM, S Jacobs, RS, CH and SG-R will conduct screening and data collection. DW assisted with psychosocial questionnaire development. Analyses will be performed by VJ, LK, CH and SM. J Swanepoel prepared the first draft of the manuscript and will assist with data analysis. All authors provided edits and critiqued the manuscript.

FUNDING STATEMENT

J Seddon is supported by a Clinician Scientist Fellowship jointly funded by the UK Medical Research Council (MRC) and the UK Department for International Development (DFID) under the MRC/DFID Concordat agreement (MR/R007942/1).

COMPETING INTERESTS

None declared

For peer review only

REFERENCES

1. Patton GC, Sawyer SM, Santelli JS, Ross DA, Afifi R, Allen NB, et al. Our future: a Lancet commission on adolescent health and wellbeing. Lancet. 2016;387:2423–78.

2. Azzopardi PS, Hearps SJC, Francis KL, Kennedy EC, Mokdad AH, Kassebaum NJ, et al. Progress in adolescent health and wellbeing: tracking 12 headline indicators for 195 countries and territories, 1990–2016. Lancet. 2019;393:1101–18.

3. Snow KJ, Sismanidis C, Denholm J, Sawyer SM, Graham SM. The incidence of tuberculosis among adolescents and young adults: a global estimate. Eur Respir J. 2018;51:1702352.

4. Seddon JA, Chiang SS, Esmail H, Coussens AK. The Wonder Years: What Can Primary School Children Teach Us About Immunity to Mycobacterium tuberculosis? Front Immunol. 2018;9.

5. Patterson B, Morrow CD, Kohls D, Deignan C, Ginsburg S, Wood R. Mapping sites of high TB transmission risk: Integrating the shared air and social behaviour of TB cases and adolescents in a South African township. Sci Total Environ. 2017;583:97–103.

6. Margarit A, Simó S, Rozas L, Deyà-Martínez À, Barrabeig I, Gené A, et al. [Adolescent tuberculosis; a challenge and opportunity to prevent community transmission]. An Pediatr (Barc). 2017;86:110–4.

7. Cruz AT, Hwang KM, Birnbaum GD, Starke JR. Adolescents With Tuberculosis. Pediatr Infect Dis J. 2013;32:937–41.

8. de Pontual L, Balu L, Ovetchkine P, Maury-Tisseron B, Lachassinne E, Cruaud P, et al. Tuberculosis in Adolescents: A French retrospective study of 52 cases. Pediatr Infect Dis J. 2006;25:930–2.

9. Ssengooba W, Kateete DP, Wajja A, Bugumirwa E, Mboowa G, Namaganda C, et al. An Early Morning Sputum Sample Is Necessary for the Diagnosis of Pulmonary Tuberculosis, Even with More Sensitive Techniques: A Prospective Cohort Study among Adolescent TB-Suspects in Uganda. Tuberc Res Treat. 2012;2012:1–6.

10. Stevens H, Ximenes RA, Dantas OM, Rodrigues LC. Risk factors for tuberculosis in older children and adolescents: a matched case–control study in Recife, Brazil. Emerg Themes Epidemiol. 2014;11:20.

11. Enane LA, Lowenthal ED, Arscott-Mills T, Matlhare M, Smallcomb LS, Kgwaadira B, et al. Loss to follow-up among adolescents with tuberculosis in Gaborone, Botswana. Int J Tuberc Lung Dis. 2016;20:1320–5.

12. Snow K, Hesseling AC, Naidoo P, Graham SM, Denholm J, du Preez K. Tuberculosis in adolescents and young adults: epidemiology and treatment outcomes in the Western Cape. Int J Tuberc Lung Dis. 2017;21:651–7.

13. Isaakidis P, Paryani R, Khan S, Mansoor H, Manglani M, Valiyakath A, et al. Poor Outcomes in a Cohort of HIV-Infected Adolescents Undergoing Treatment for Multidrug-Resistant Tuberculosis in Mumbai, India. Wilkinson RJ, editor. PLoS One. 2013;8:e68869.

14. Ravimohan S, Kornfeld H, Weissman D, Bisson GP. Tuberculosis and lung damage: from epidemiology to pathophysiology. Eur Respir Rev. 2018;27:170077.

For peer review only

15. Allwood BW, Byrne A, Meghji J, Rachow A, van der Zalm MM, Schoch OD. Post-Tuberculosis Lung Disease: Clinical Review of an Under-Recognised Global Challenge. Respiration. 2021;100:751–63.

16. Stocks J, Hislop A, Sonnappa S. Early lung development: lifelong effect on respiratory health and disease. Lancet Respir Med. 2013;1:728–42.

17. Meghji J, Mortimer K, Agusti A, Allwood BW, Asher I, Bateman ED, et al. Improving lung health in low-income and middle-income countries: from challenges to solutions. Lancet (London, England). 2021;397:928–40.

18. Pasipanodya JG, McNabb SJ, Hilsenrath P, Bae S, Lykens K, Vecino E, et al. Pulmonary impairment after tuberculosis and its contribution to TB burden. BMC Public Health. 2010;10:259.

19. Chung K-P, Chen J-Y, Lee C-H, Wu H-D, Wang J-Y, Lee L-N, et al. Trends and predictors of changes in pulmonary function after treatment for pulmonary tuberculosis. Clinics. 2011;66:549–56.

20. Nagata JM, Hathi S, Ferguson BJ, Hindin MJ, Yoshida S, Ross DA. Research priorities for adolescent health in low- and middle-income countries: A mixed-methods synthesis of two separate exercises. J Glob Health. 2018;8.

21. World Health Organization. Roadmap towards ending TB in children and adolescents. [Internet]. Geneva; 2018. Available from: http://apps.who.int/iris/bitstream/handle/10665/274374/9789241514668-eng.pdf?ua=1

22. Kanabus A. TB Statistics South Africa - Incidence, prevalence [Internet]. 2015. Available from: https://tbfacts.org/tb-statistics-south-africa/

23. Mahomed H, Ehrlich R, Hawkridge T, Hatherill M, Geiter L, Kafaar F, et al. TB Incidence in an Adolescent Cohort in South Africa. Ruhwald M, editor. PLoS One. 2013;8:e59652.

24. Johnson L, Dorrington R. Thembisa Version 4.5: A Model for Evaluating the Impact of HIV/AIDS in South Africa. [Internet]. 2022 [cited 2022 Sep 7]. Available from: https://www.thembisa.org/content/downloadPage/Provinces2022

25. Quanjer PH, Stanojevic S, Cole TJ, Baur X, Hall GL, Culver BH, et al. Multi-ethnic reference values for spirometry for the 3–95-yr age range: the global lung function 2012 equations. Eur Respir J. 2012;40:1324–43.

26. Smith S-J, Gray DM, MacGinty RP, Hall GL, Stanojevic S, Mphahlele R, et al. Choosing the Better Global Lung Initiative 2012 Equation in South African Population Groups. Am J Respir Crit Care Med. 2020;202:1724–7.

27. ATS Statement. Am J Respir Crit Care Med. 2002;166:111–7.

28. Mylius CF, Paap D, Takken T. Reference value for the 6-minute walk test in children and adolescents: a systematic review. Expert Rev Respir Med. 2016;10:1335–52.

29. Wiseman CA, Gie RP, Starke JR, Schaaf HS, Donald PR, Cotton MF, et al. A Proposed Comprehensive Classification of Tuberculosis Disease Severity in Children. Pediatr Infect Dis J. 2012;31:347–52.

For peer review only

For peer review only

Social Science Questionnaire

Participant Identifier (PID)

____

(TT _________)

Sociodemographic / Household Information

Kolu luhlu kukho imibuzo abantu abaninzi bazocinga zingqalene nabo ngqo. Ndifuna ukukukukhumbuza ukuthatha kwakho inxaxheba kuxhomekeke kuwe. Kwaye zonke inkcukacha/lonke ulwazi othe wasabela ngalo koluluhlu lwemibuzo luzokunonophelwa kwimeko esemfihlakalweni (ngaphandle kokuba uchaza ukuba uzakuzilimaza okanye uzakulimaza abanye). Lento ithetha ukuba impendulo zakho asizikuzabela abanakekeli bempilo yakho okanye usapho lwakho. Zonke ezinkcukacha zizohlala emfihlakalweni/ekungaziweni kwaye zizokobiwa kumalungu woluphando.

In this survey there are questions that many people will think of as 'personal'. I want to remind you that your participation in this survey is completely voluntary. Additionally, all the information that you share with us on this survey will be monitored in a strictly confidential manner (unless you state that you are going to hurt yourself or others). This means that we will not share your answers with your medical providers or your family. All of the information will remain anonymous and be shared only with members of our study team.

In hierdie opname is daar vrae wat baie mense as 'persoonlik' sal beskou. Ek wil jou daaraan herinner dat jou deelname aan hierdie opname heeltemal vrywillig is. Daarbenewens sal al die inligting wat jy met ons deel in hierdie opname op 'n streng vertroulike wyse gemonitor word (tensy jy verklaar dat jy jouself of ander gaan seermaak). Dit beteken dat ons nie jou antwoorde met jou mediese verskaffers of jou familie sal deel nie. Al die inligting sal anoniem bly en slegs met lede van ons studiespan gedeel word.

◯ Next
(Read to participant)

Sithini isini sakho?

What is your sex?

Wat is jou geslag?

◯ Ibhinqa # Female # Vroulik
◯ Indoda # Male # Manlik
(Read to the participant; do not read the response options aloud)

For peer review only

Isifo sephepha ngelinye ixesha singancamatheliswa nezinye izifo, ezifana ne HIV. Singathanda ukubuza ukuba wena uphila ne HIV?<br>Ungaphendula ewe, hayi okanye ukuba ukhetha ukungaphenduli?<br><br>TB can sometimes be associated with other illnesses, such as HIV. We would like to ask if you are living with HIV?<br>You can answer yes, no or that you prefer not to answer?<br><br>TB kan soms geassosieer word met ander siektes, soos MIV. Ons wil vra of jy met MIV leef?<br>Jy kan ja of nee antwoord of dat jy verkies om nie te antwoord nie?	◯ Ndiphila ne HIV # I am living with HIV # Ek leef met MIV<br>◯ Andiphili ne HIV # I am not living with HIV # Ek leef nie met MIV nie<br>◯ Ndikhetha ungaphenduli # I prefer not to answer # Ek verkies om nie te antwoord nie<br>(Ask the participant; and read the response options aloud)
Uhlala nabani?<br><br>Who do you live with?<br><br>By wie woon jy?	☐ Mama # Mother # Ma<br>☐ Tata # Father # Pa<br>☐ Umama wesibin # Stepmother # Stiefma<br>☐ Utata wesibini # Stepfather # Stiefpa<br>☐ Oobhuti # Brother(s) # Broer(s)<br>☐ Usisi # Sister(s) # Suster(s)<br>☐ Oomakhulu noo Tamkhulu # Grandparent(s) # Grootouers<br>☐ OoMakazi # Aunt(s) # Tannie(s)<br>☐ OoMalume # Uncle(s) # Oom (s)<br>☐ Omnye # Other # Ander<br>(Ask the participant; and read the response options aloud)
Uhlala nabani?<br><br>Who do you live with?<br><br>By wie woon jy?	____________________<br>(If Other, specify)
Inani lo bhuti kunye noo sisi abangaphantsi kweminyaka eyi 18 (ngaphandle kwakho)<br><br>Number of brothers/sisters younger than 18 years old (not including yourself)<br><br>Aantal broers/susters jonger as 18 jaar (nie jouself ingesluit nie)	____________________<br>(Ask the participant)
Inani lo bhuti kunye noo sisi abangaphantsi kweminyaka eyi 18 (ngaphandle kwakho)<br><br>Number of brothers/sisters 18 years or older (not including yourself)<br><br>Aantal broers/susters 18 jaar of ouer (nie jouself ingesluit nie)	____________________<br>(Ask the participant)

For peer review only

Phambi kokuba ubenesifo sephepha, ngubani obesebenza kusapho lwakho ukwenzela niphile? (khetha konke okufanelekileyo)<br><br>Before you had TB, who was working in your family to make a living? (select all that apply)<br><br>Wie het in jou gesin gewerk voordat jy TB gehad het? (kies alles wat van toepassing is)	☐ Mama # Mother # Ma<br>☐ Tata # Father # Pa<br>☐ Umama wesibin # Stepmother # Stiefma<br>☐ Utata wesibini # Stepfather # Stiefpa<br>☐ Oobhuti # Brother(s) # Broer(s)<br>☐ Usisi # Sister(s) # Suster(s)<br>☐ Oomakhulu noo Tamkhulu # Grandparent(s) # Grootouers<br>☐ OoMakazi # Aunt(s) # Tannie(s)<br>☐ OoMalume # Uncle(s) # Oom (s)<br>☐ Omnye # Other # Ander<br>(Read to the participant; and read the response options)
Phambi kokuba ubenesifo sephepha, ngubani obesebenza kusapho lwakho ukwenzela niphile? Ukuba omnye, uyacelwa cacisa<br><br>Before you had TB, who was working in your family to make a living? If Other, please specify<br><br>Wie het in jou gesin gewerk voordat jy TB gehad het? Indien ander, spesifiseer asseblief.	________________<br>(If Other, specify)
Emva kokuba uqale uthatha unyango lwesifo sephepha, ngubani kusapho lwakho ebesebenzela ukuzisa umvuzo kwikhaya lakho?<br><br>After you began TB treatment, who in your family was working to earn an income for your home?<br><br>Nadat jy met TB behandeling begin het, wie in jou gesin het gewerk om 'n inkomste vir jou huis te verdien?	☐ Mama # Mother # Ma<br>☐ Tata # Father # Pa<br>☐ Umama wesibin # Stepmother # Stiefma<br>☐ Utata wesibini # Stepfather # Stiefpa<br>☐ Oobhuti # Brother(s) # Broer(s)<br>☐ Usisi # Sister(s) # Suster(s)<br>☐ Oomakhulu noo Tamkhulu # Grandparent(s) # Grootouers<br>☐ OoMakazi # Aunt(s) # Tannie(s)<br>☐ OoMalume # Uncle(s) # Oom (s)<br>☐ Omnye # Other # Ander<br>(Ask the participant; and read the response options aloud)
Emva kokuba uqale uthatha unyango lwesifo sephepha, ngubani kusapho lwakho ebesebenzela ukuzisa umvuzo kwikhaya lakho? ukuba omnye cacisa.<br><br>After you began TB treatment, who in your family was working to earn an income for your home? If Other, please specify<br><br>Nadat jy met TB behandeling begin het, wie in jou gesin het gewerk om 'n inkomste vir jou huis te verdien? Indien ander, spesifiseer asseblief.	________________<br>(If Other, specify)
Ingaba ngoku uyafunda eskolweni/ kwindawo zemfundo enomsila/ dyunivesithi? (khetha ibenye)<br><br>Are you currently attending a school / institution / university? (Select ONE)<br><br>Woon jy tans 'n skool / instelling / universiteit by? (Kies EEN)	○ Ewe kodwa hayi ngenxa yesigulo sam # Yes but not now because of my illness # Ja maar nie nou nie weens my siekte<br>○ Ewe kwaye ndiyaqhubeka nokungena izifundo # Yes and I am currently taking classes # Ja en ek neem tans klasse<br>○ Hayi # No # Nee<br>(Ask the participant; and read the response options aloud)

For peer review only

Lithini izinga eliphezulu lwemfundo utatakho aphele kulo? (okanye utata wakho ongakuzaliyo ukuba umthatha njengomama wakho)<br><br>What is the highest level of education your father completed? (or your stepfather if you consider him as your father)<br><br>Wat is die hoogste vlak van opleiding wat jou pa voltooi het? (of jou stiefpa as jy hom as jou pa beskou)	◯ Kwesinye isikolo sebanga eliphezulu # Some high school # hoërskool<br>◯ Indawo yemfundo ephezulu okanye Idyunivesithi # Institute or University # Instituut of Universiteit<br>◯ Akho nanye # None # Geen<br>◯ Ayaziwa # Unknown # Onbekend<br>(Ask the participant; and read the response options aloud)
Lithini izinga eliphezulu lwemfundo umamakho aphele kulo? (okanye umama wakho ongakuzaliyo ukuba umthatha njengomama wakho)<br><br>What is the highest level of education your mother obtained? (or your stepmother if you consider her as your mother)<br><br>Wat is die hoogste vlak van opleiding wat jou ma verwerf het? (of jou stiefma as jy haar as jou moeder beskou)	◯ Kwesinye isikolo sebanga eliphezulu # Some high school # hoërskool<br>◯ Indawo yemfundo ephezulu okanye Idyunivesithi # Institute or University # Instituut of Universiteit<br>◯ Akho nanye # None # Geen<br>◯ Ayaziwa # Unknown # Onbekend<br>(Ask the participant; and read the response options aloud)
Kumbuzo olandelayo, sisicelo ukuba uphendule ngenye yezimpendulo zilandelayo: Ngamaxesha onke, Ngamaxeshe amaninzi, Ngamanye amaxesha, Manqaphanqapha okanye Soze.<br><br>For the next question, please respond with ONE of the following options: Always, Often, Sometimes, Rarely, or Never.<br><br>Beantwoord die volgende vraag met EEN van die volgende opsies: Altyd, Gereeld, Soms, Selde/Min of Nooit.	◯ Next<br>(Read to the participant)
Ukwazi ukuba kubalulekile ukutya kathathu ngemini ukwenzele ubesempilweni, kukangaphi apho uthe watya ngaphantsi kunoku ngesuku ngenxa yokungabikho kwemali eyaneleyo endlini yokuthenga ukutya?<br><br>Knowing that it is necessary to eat 3 times per day for a healthy diet, how often have you had to eat less on per day because there was not enough money in the house to buy food?<br><br>Met die wete dat dit nodig is om drie keer per dag te eet vir 'n gesonde dieet, hoe gereeld moes jy minder per dag eet omdat daar nie genoeg geld in die huis was om kos te koop nie?	◯ Maxesha wonkeMaxesha wonke # Always # Altyd<br>◯ Maxesha amaninzi # Often # Gereeld<br>◯ Ngamanye amaxesha # Sometimes # Soms<br>◯ Manqaphanqapha # Rarely # Selde<br>◯ Zange/soze # Never # Nooit nie<br>(Read to the participant; do not read the response options aloud)

For peer review only

TB Symptoms

Ngoku sizokuthanda ukubuza imibuzo embalwa malunga nempawo obukhe waphantsi kwazo phambi kokuba / ngelaxesha/ emva kokuba uhlaselwe sisifo sephepha

Now we'd like to ask a few questions about the symptoms you experienced before / during / after your TB episode.

Nou wil ons 'n paar vrae vra oor die simptome wat jy voor / tydens / na jou TB-episode ervaar het.

◯ Next
(Read to the participant)

Zeziphi iimpawo uthe wanazo phambi kokufumana unyango lwesifo sephepha (khetha konke okufanelekileyo)

What symptoms did you have before receiving treatment for TB? (select ALL that apply)

Watter simptome het jy gehad voordat jy TB behandeling ontvang het? (kies ALLES wat van toepassing is)

- ☐ Umkhuhlane # Fever # Koors
- ☐ Ingqele (ukugodola okulandelwa kukungcangcazela komzimba) # Chills (intense cold sensation followed by slight tremors of the body) # Kouekoors (intense koue sensasie gevolg deur ligte bewings in die liggaam)
- ☐ Ukudinwa # Fatigue # Moegheid
- ☐ Ukungacaceli ukutya # Loss of appetite # Eetlus verloor
- ☐ Ukubila ebusuku # Sweating at night # Sweet in die nag
- ☐ Ukugabha # Vomiting # Braking
- ☐ Ukwehla emzimbeni # Weight loss # Gewigsverlies
- ☐ Ukuhlelwa ligazi # Hemoptysis (losing blood) # Hemoptise (bloed verloor)
- ☐ Khange ndibenazimpawo # I did not have symptoms # Ek het nie simptome gehad nie
- ☐ Omnye # Other # Ander

(Ask the participant; and read the response options aloud)

Zeziphi iimpawo uthe wanazo phambi kokufumana unyango lwesifo sephepha? ukuba omnye cacisa

What symptoms did you have before receiving treatment for TB? Specify

Watter simptome het jy gehad voordat jy TB behandeling ontvang het? Spesifiseer

____
(If Other, specify)

Ibelixesha elingakanani unezimpawo phambi kokuba ugqirha akuxelele ukuba unesifo sephepha?

For how long did you have these symptoms before the doctor told you that you have TB?

Hoe lank het jy hierdie simptome gehad voordat die dokter vir jou vertel het dat jy TB het?

- ◯ Phakathi kwentsuku eziyi 15 ukuya kwinyanga ey 1 # Between 15 days - 1 month # Tussen 15 dae - 1 maand
- ◯ inyanga eyi 1 ukuya kweziyi 2 # 1 - 2 months # 1 - 2 maande
- ◯ iinyanga eziyi 2 ukuya kweziyi 3 # 2 - 3 months # 2 - 3 maande
- ◯ ngapha kweenyanga eziyi 4 # More than 4 months # Meer as 4 maande

(Ask the participant; and read the response options aloud)

For peer review only

Zeziphi iimpawo onazo ngoku? (khetha okufanelekileyo)<br><br>What symptoms do you currently have? (select ALL that apply)<br><br>Watter simptome het jy tans? (kies alles wat van toepassing is)	☐ Umkhuhlane # Fever # Koors<br>☐ Ingqele (ukugodola okulandelwa kukungcangcazela komzimba) # Chills (intense cold sensation followed by slight tremors of the body) # Kouekoors (intense koue sensasie gevolg deur ligte bewings in die liggaam)<br>☐ Ukudinwa # Fatigue # Moegheid<br>☐ Ukungacaceli ukutya # Loss of appetite # Eetlus verloor<br>☐ Ukubila ebusuku # Sweating at night # Sweet in die nag<br>☐ Ukugabha # Vomiting # Braking<br>☐ Ukwehla emzimbeni # Weight loss # Gewigsverlies<br>☐ Ukuhlelwa ligazi # Hemoptysis (losing blood) # Hemoptise (bloed verloor)<br>☐ Khange ndibenazimpawo # I did not have symptoms # Ek het nie simptome gehad nie<br>☐ Omnye # Other # Ander<br>(Ask the participant; and read the response options aloud)
Zeziphi iimpawo onazo ngoku? Cacisa<br><br>What symptoms do you currently have? Specify<br><br>Watter simptome het jy tans? Spesifiseer	______________________________<br>(If Other, specify)
Mangaphi amayeza owathathayo ukwenzela isifo sephepha?<br><br>How many medications do you take for your TB?<br><br>Hoeveel medikasies neem jy vir jou TB?	________________________<br>(Ask the participant)
Ngokuqhelekiyo, uyanjani kwiziko lwezonyango (kliniki) ukuyothatha amayeza wakho (khetha konke okufanekileyo)<br><br>Typically, how do you get to the health facility (clinic) to take your medications? (select ALL that apply)<br><br>Hoe kom jy gewoonlik by die gesondheidsfasiliteit (kliniek) om medisyne in te neem? (kies alles wat van toepassing is)	☐ ngeenyawo # On-foot # Op voet<br>☐ Imoto - itaxi # Motor-taxi # Motortaxi<br>☐ Bhasi # Bus # Bus<br>☐ I-taxi # Taxi # Taxi<br>☐ Imoto encinci # Private car # Privaat motor<br>☐ Enye # Other # Ander<br>(Ask the participant; and read the response options aloud)
Ngokuqhelekiyo, uyanjani kwiziko lwezonyango (kliniki) ukuyothatha amayeza wakho? Cacisa<br><br>Typically, how do you get to the health facility (clinic) to take your medications? Specify<br><br>Hoe kom jy gewoonlik by die gesondheidsfasiliteit (kliniek) om jou medikasie te neem? Spesifiseer	______________________________<br>(If Other, specify)

For peer review only

Ngokuqhelekileyo, uthatha ixesha elingakanani ukuyofika kwiziko lonyango (kliniki)? (minutes)<br><br>Typically, how long does it take you to arrive to health facility (clinic)? (minutes)<br><br>Hoe lank neem dit gewoonlik om by die gesondheidsfasiliteit (kliniek) aan te kom? (minute)	______________________<br>(Ask the participant)
Ngokuqhelekileyo, ngubani obhatalela isithuthi ukuze ufike kwiziko lonyango (kliniki) (khetha konke okufanelekileyo)<br><br>Typically, who pays for your transportation to arrive to the health facility (clinic)? (select ALL that apply)<br><br>Wie betaal gewoonlik vir jou vervoer om na die gesondheidsfasiliteit (kliniek) te kom? (kies alles wat van toepassing is)	☐ Mna # Myself # Myself<br>☐ Mama # Mother # Ma<br>☐ Tatam # Father # Pa<br>☐ Umama wesibin # Stepmother # Stiefma<br>☐ Utata wesibini # Stepfather # Stiefpa<br>☐ Oobhuti # Brother # Broer<br>☐ uSisi # Sister # Suster<br>☐ Oomakhulu noo Tamkhulu # Grandparent(s) # Grootouers<br>☐ OoMakazi # Aunt(s) # Tannie(s)<br>☐ OoMalume # Uncle(s) # Oom(s)<br>☐ Omnye # Other # Ander<br>(Ask the participant; and read the response options aloud)
Ngokuqhelekileyo, ngubani obhatalela isithuthi ukuze ufike kwiziko lonyango (kliniki)? Cacisa<br><br>Typically, who pays for your transportation to arrive to the health facility (clinic)? Specify<br><br>Wie betaal gewoonlik vir jou vervoer om na die gesondheidsfasiliteit (kliniek) te kom? Spesifiseer	______________________<br>(If Other, specify)
Yeyiphi eminye imiphumela okanye ukungakhululeki onako okanye othe wanako ngenxa yamayeza? (khetha konke okufanelekileyo)<br><br>What side effects or discomfort do you have, or have you had because of the medications? (select ALL that apply)<br><br>Watter newe-effekte of ongemak ervaar jy of het jy ervaar as gevolg van die medikasie? (kies ALLES wat van toepassing is)	☐ Rhashalala # Rash # Uitslag<br>☐ Ukugabha # Vomiting # Braking<br>☐ Intloko ebuhlungu # Headache # Hoofpyn<br>☐ Isizaphu zaphu # Nausea # Naarheid<br>☐ Iintlungu kumazantsi esisu # Abdominal pain # Maagpyn<br>☐ Ukudinwa # Fatigue # Moegheid<br>☐ Akho kuhlaliseka kakubi # No discomfort # Geen ongemak nie<br>☐ Okunye # Other # Ander<br>(Ask the participant; and read the response options aloud)
Yeyiphi eminye imiphumela okanye ukungakhululeki onako okanye othe wanako ngenxa yamayeza? Cacisa<br><br>What side effects or discomfort do you have, or have you had because of the medications? Specify<br><br>Watter newe-effekte of ongemak ervaar jy of het jy ervaar as gevolg van die medikasie? Spesifiseer	______________________<br>(If Other, specify)

For peer review only

Kukangaphi usiva ukungakhululeki ngenxa yothatha amayeza wakho wesifo sephepha?<br><br>How often do you experience discomfort as a result of taking your TB medications?<br><br>Hoe gereeld ervaar jy ongemak as gevolg van jou medikasie teen TB?	◯ Maxesha wonkeMaxesha wonke # Always # Altyd<br>◯ Maxesha amaninzi # Often # Gereeld<br>◯ Ngamanye amaxesha # Sometimes # Soms<br>◯ Manqaphanqapha # Rarely # Selde<br>◯ Zange/soze # Never # Nooit nie<br>(Ask the participant; and read the response options aloud)
Zingaphi intsuku zonyango othe waziphosa kwiveki edluleyo?<br><br>How many days of treatment have you missed in the past week?<br><br>Hoeveel dae se behandeling het jy die afgelope week misgeloop?	____________________<br>(Ask the participant)
Zingaphi intsuku zonyango othe waziphosa kwiveki ezimbini edluleyo?<br><br>How many days of treatment have you missed in the past 2 weeks?<br><br>Hoeveel dae se behandeling het jy die afgelope twee weke misgeloop?	____________________<br>(Ask the participant)
Zingaphi iintsuku zinyango othe waziphosa kwiiveki ezintathu ezidlulileyo?<br><br>How many days of treatment have you missed in the past 3 weeks?<br><br>Hoeveel dae se behandeling het jy die afgelope drie weke misgeloop?	____________________<br>(Ask the participant)

Family Relationships

Ngoku sisozothanda ukubuza imibuzo embalwa malunga ngobudlelwane bosapho lwakho.<br><br>Now we'd like to ask a few questions about your family relationships.<br><br>Nou wil ons 'n paar vrae vra oor jou gesinsverhoudinge.	◯ Next<br>(Read to participant)

For peer review only

Cinga ngomntu ongumntu ozakukwenzela izigqibo ezisemthethweni (umzekelo akusayinele impepha ngcwadi). Ukuba useminyakeni efanelekileyo (18 okanye ngaphezulu) cinga ngoku owakwenzela oku ngelixa wawungaphantsi kwe 18. (Khetha ibenye)<br><br>Think of someone that is the person who would have to make legal decisions for you (for example, sign a document). If you are of age (18 or older), think of someone who did this when you were less than 18.<br><br>Dink aan 'n persoon wat regsbesluite vir jou moet neem (byvoorbeeld, teken van 'n dokument). As jy ouer is (18 of ouer), dink aan iemand wat dit gedoen het toe jy jonger as 18 was. (Kies een)	◯ Mama # Mother # Ma<br>◯ Tata # Father # Pa<br>◯ Mntakwe/ dade # Sibling # Broers en Susters<br>◯ Makhulu/Tamkhulu # Grandparent # Grootouer<br>◯ Makazi # Aunt # Tannie<br>◯ Malume # Uncle # Oom<br>(Ask the participant; and read the response options aloud)
Phendula kwimibuzo elandelayo, nalomntu uthe wamkhetha kulombuzo udlulileyo. Wonke amaxesha simane sisithi "mama, tata, umgcini mntu," cinga ngalomntu, kwaye ngalomntu kuphela. Kwimibuzo elandelayo, uyacelwa ukuba uphendule ngokukhetha enye yezimpendulo zilandelayo: Ngamaxesha onke, Ngamaxesha amaninzi, Ngamaxesha athile, Manqaphanqapha okanye Soze.<br><br>Respond to the following questions, with the person you chose in the previous question in mind. Every time we say "mother / father / guardian," think of this person, and only this person. In the following questions, please respond by selecting ONE of the following options: Always, Often, Sometimes, Rarely, Never.<br><br>Beantwoord die volgende vrae met die persoon wat jy in die vorige vraag gekies het in gedagte. Dink aan hierdie persoon en net aan hierdie persoon elke keer as ons 'moeder / vader / voog' sê. Beantwoord die volgende vrae deur EEN van die volgende opsies te kies: Altyd, Gereeld, Soms, Selde/Min, Nooit.	◯ Next<br>(Read to the participant)
Umamam/ tata/ umgcini wam undiphatha ngenkuthalo/ ngobubele.<br><br>My mother / father / guardian treats me with kindness.<br><br>My ma / pa / voog behandel my met vriendelikheid.	◯ Maxesha wonke # Always # Altyd<br>◯ Maxesha amaninzi # Often # Gereeld<br>◯ Ngamanye amaxesha # Sometimes # Soms<br>◯ Manqaphanqapha # Rarely # Selde<br>◯ Zange/soze # Never # Nooit nie<br>(Ask the participant; do not read the response options aloud)
Ndiyavana nomama / tata/umgcini wam.<br><br>I get along well with my mother / father / guardian.<br><br>Ek kom goed oor die weg met my ma / pa / voog.	◯ Maxesha wonke # Always # Altyd<br>◯ Maxesha amaninzi # Often # Gereeld<br>◯ Ngamanye amaxesha # Sometimes # Soms<br>◯ Manqaphanqapha # Rarely # Selde<br>◯ Zange/soze # Never # Nooit nie<br>(Ask the participant; do not read the response options aloud)

For peer review only

Ndiyavana namanye amalungu osapho.<br><br>I get along well with other family members.<br><br>Ek kom goed oor die weg met ander gesinslede/familielede.	◯ Maxesha wonke # Always # Altyd<br>◯ Maxesha amaninzi # Often # Gereeld<br>◯ Ngamanye amaxesha # Sometimes # Soms<br>◯ Manqaphanqapha # Rarely # Selde<br>◯ Zange/soze # Never # Nooit nie<br>(Ask the participant; do not read the response options aloud)
Ndiyathembela kusapho lwam<br><br>I confide in my family<br><br>Ek vertrou my gesin/familie om persoonlike of sensitiewe inligting met hulle te deel.	◯ Maxesha wonke # Always # Altyd<br>◯ Maxesha amaninzi # Often # Gereeld<br>◯ Ngamanye amaxesha # Sometimes # Soms<br>◯ Manqaphanqapha # Rarely # Selde<br>◯ Zange/soze # Never # Nooit nie<br>(Ask the participant; do not read the response options aloud)
Ndineemfihlo endingazithethi nomama /tata/ umgcini.<br><br>I have secrets that I do not share with my mother / father / guardian.<br><br>Ek het geheime wat ek nie deel met my ma / pa / voog nie.	◯ Maxesha wonke # Always # Altyd<br>◯ Maxesha amaninzi # Often # Gereeld<br>◯ Ngamanye amaxesha # Sometimes # Soms<br>◯ Manqaphanqapha # Rarely # Selde<br>◯ Zange/soze # Never # Nooit nie<br>(Ask the participant; do not read the response options aloud)
Umama/ tata/ umgcini wam uyandixhasa/uyandikhuthaza ngokophefumlo malunga nonyango lwam lwesifo sephepha.<br><br>My mother / father / guardian supports me emotionally with my TB treatment.<br><br>My ma / pa / voog ondersteun my emosioneel met my TB behandeling.	◯ Maxesha wonke # Always # Altyd<br>◯ Maxesha amaninzi # Often # Gereeld<br>◯ Ngamanye amaxesha # Sometimes # Soms<br>◯ Manqaphanqapha # Rarely # Selde<br>◯ Zange/soze # Never # Nooit nie<br>(Ask the participant; do not read the response options aloud)
Amanye amalungu osapho ayandixhasa ngokomphefulo malunga nonyango lwam lwesifo sephepha.<br><br>Other family members support me emotionally with my TB treatment.<br><br>Ander gesinslede/familielede ondersteun my emosioneel met my TB behandeling.	◯ Maxesha wonke # Always # Altyd<br>◯ Maxesha amaninzi # Often # Gereeld<br>◯ Ngamanye amaxesha # Sometimes # Soms<br>◯ Manqaphanqapha # Rarely # Selde<br>◯ Zange/soze # Never # Nooit nie<br>(Ask the participant; do not read the response options aloud)
Abahlobo bam bandiyandixhasa ngokusemphefumlweni malunga nonyango lwam lwesifo sephepha.<br><br>My friends support me emotionally with my TB treatment.<br><br>My vriende ondersteun my emosioneel met my TB behandeling.	◯ Maxesha wonke # Always # Altyd<br>◯ Maxesha amaninzi # Often # Gereeld<br>◯ Ngamanye amaxesha # Sometimes # Soms<br>◯ Manqaphanqapha # Rarely # Selde<br>◯ Zange/soze # Never # Nooit nie<br>(Ask the participant; do not read the response options aloud)

For peer review only

Nje kengoku, umama / tata / umgcini wam nam siyayifumana inkxaso ngokomphefumlo kwamanye amalungu osapho.<br><br>Generally, my mother / father / guardian and I have always had emotional support from other family members.<br><br>Oor die algemeen het ek en my ma / pa / voog altyd emosionele ondersteuning van ander familielede gehad.	◯ Maxesha wonke # Always # Altyd<br>◯ Maxesha amaninzi # Often # Gereeld<br>◯ Ngamanye amaxesha # Sometimes # Soms<br>◯ Manqaphanqapha # Rarely # Selde<br>◯ Zange/soze # Never # Nooit nie<br>(Ask the participant; do not read the response options aloud)
Umntu wasekhaya uyandikhathapha xandisiya kuthatha amayeza wam kwinyango lwezempilo.<br><br>Someone from home accompanies me when I go to get my medications at the health centre.<br><br>Iemand van die huis vergesel my as ek my medikasie by die gesondheidsentrum gaan haal.	◯ Maxesha wonke # Always # Altyd<br>◯ Maxesha amaninzi # Often # Gereeld<br>◯ Ngamanye amaxesha # Sometimes # Soms<br>◯ Manqaphanqapha # Rarely # Selde<br>◯ Zange/soze # Never # Nooit nie<br>(Ask the participant; do not read the response options aloud)
Amanye amalungu wosapho (ngaphandle kwabo ndihlala nabo/ndizalwa nabo, ngamanye amagama ngaphandle kwabazali or izalamane/abaninawa) bayayazi ukuba ndinesifo sephepha.<br><br>My other family members (outside of my immediate family, in other words people other than parents or siblings) know that I have TB.<br><br>My ander familielede (buite my onmiddellike gesin, met ander woorde mense anders as my ouers of broers en susters) weet dat ek TB het.	◯ Maxesha wonke # Always # Altyd<br>◯ Maxesha amaninzi # Often # Gereeld<br>◯ Ngamanye amaxesha # Sometimes # Soms<br>◯ Manqaphanqapha # Rarely # Selde<br>◯ Zange/soze # Never # Nooit nie<br>(Ask the participant; do not read the response options aloud)
Umama / tata / umgcini wam akabathandi abahlobo bam.<br><br>My mother / father / guardian does not like my friends.<br><br>My ma / pa / voog hou nie van my vriende nie.	◯ Maxesha wonke # Always # Altyd<br>◯ Maxesha amaninzi # Often # Gereeld<br>◯ Ngamanye amaxesha # Sometimes # Soms<br>◯ Manqaphanqapha # Rarely # Selde<br>◯ Zange/soze # Never # Nooit nie<br>(Ask the participant; do not read the response options aloud)
Ndiyalwa nomama / tata / umgcini wam.<br><br>I fight with my mother / father / guardian.<br><br>Ek baklei met my ma / pa / voog	◯ Maxesha wonke # Always # Altyd<br>◯ Maxesha amaninzi # Often # Gereeld<br>◯ Ngamanye amaxesha # Sometimes # Soms<br>◯ Manqaphanqapha # Rarely # Selde<br>◯ Zange/soze # Never # Nooit nie<br>(Ask the participant; do not read the response options aloud)
Isigulo sam sesifo sephepha ibangela abazali bam kunye namalungu osapho kubekho ingxoxo/ingxabano.<br><br>My TB illness cases my parents and family members to argue.<br><br>My TB-siekte veroorsaak dat my ouers en familielede stry.	◯ Maxesha wonke # Always # Altyd<br>◯ Maxesha amaninzi # Often # Gereeld<br>◯ Ngamanye amaxesha # Sometimes # Soms<br>◯ Manqaphanqapha # Rarely # Selde<br>◯ Zange/soze # Never # Nooit nie<br>(Ask the participant; do not read the response options aloud)

For peer review only

Nje kengoku, Ndonwabile ngobudlelwane bam nomama / tata / umgcini wam.<br><br>Generally, I am happy with my relationship with my mother / father / guardian.<br><br>Oor die algemeen is ek gelukkig met my verhouding met my ma / pa / voog.	◯ Maxesha wonke # Always # Altyd<br>◯ Maxesha amaninzi # Often # Gereeld<br>◯ Ngamanye amaxesha # Sometimes # Soms<br>◯ Manqaphanqapha # Rarely # Selde<br>◯ Zange/soze # Never # Nooit nie<br>(Ask the participant; do not read the response options aloud)
Xa ndiphuma nabahlobo, umama / tata / umgcini wam uyandiyeka ndikhetha lobuya kwam endlini.<br><br>When I go out with friends, my mother / father / guardian lets me decide the time I return home.<br><br>As ek saam met vriende uitgaan, laat my ma / pa / voog my toe om te besluit watter tyd ek terugkeer huis toe.	◯ Maxesha wonke # Always # Altyd<br>◯ Maxesha amaninzi # Often # Gereeld<br>◯ Ngamanye amaxesha # Sometimes # Soms<br>◯ Manqaphanqapha # Rarely # Selde<br>◯ Zange/soze # Never # Nooit nie<br>(Ask the participant; do not read the response options aloud)
Umama / tata / umgcini wam uyandiyeka ndizikhethele abahlobo endiphuma nabo.<br><br>My mother / father / guardian lets me decide which friends I go out with.<br><br>My ma / pa / voog laat my toe om te besluit met watter vriende ek uitgaan.	◯ Maxesha wonke # Always # Altyd<br>◯ Maxesha amaninzi # Often # Gereeld<br>◯ Ngamanye amaxesha # Sometimes # Soms<br>◯ Manqaphanqapha # Rarely # Selde<br>◯ Zange/soze # Never # Nooit nie<br>(Ask the participant; do not read the response options aloud)
Umama / tata / umgcini wam uyandiyeka ndizikhethele ixesha lokuba ndilala.<br><br>My mother / father / guardian lets me decide what time I go to bed.<br><br>My ma / pa / voog laat my toe om te besluit watter tyd ek bed toe gaan.	◯ Maxesha wonke # Always # Altyd<br>◯ Maxesha amaninzi # Often # Gereeld<br>◯ Ngamanye amaxesha # Sometimes # Soms<br>◯ Manqaphanqapha # Rarely # Selde<br>◯ Zange/soze # Never # Nooit nie<br>(Ask the participant; do not read the response options aloud)
Umama / tata / umgcini wam uyandiyeka ndikhethe ukuba ndilichitha njani ixesha endinalo.<br><br>My mother / father / guardian lets me decide how I spend my free time.<br><br>My ma / pa / voog laat my besluit hoe ek my vrye tyd spandeer.	◯ Maxesha wonke # Always # Altyd<br>◯ Maxesha amaninzi # Often # Gereeld<br>◯ Ngamanye amaxesha # Sometimes # Soms<br>◯ Manqaphanqapha # Rarely # Selde<br>◯ Zange/soze # Never # Nooit nie<br>(Ask the participant; do not read the response options aloud)
Umama / tata / umgcini wam uyandiyeka ndikhetha ukutya endikwazi ukutya.<br><br>My mother / father / guardian lets me decide what foods I can eat.<br><br>My ma / pa / voog laat my besluit watter kos ek kan eet	◯ Maxesha wonke # Always # Altyd<br>◯ Maxesha amaninzi # Often # Gereeld<br>◯ Ngamanye amaxesha # Sometimes # Soms<br>◯ Manqaphanqapha # Rarely # Selde<br>◯ Zange/soze # Never # Nooit nie<br>(Ask the participant; do not read the response options aloud)

For peer review only

My Medical Care

Ngoku sizokubuza imibuzo malunga namava wakho malunga nofikelelo kwindawo zezempilo kunye nempatho othe wayifumana kulamaziko wezempilo. Njengakuqala, uyacelwa uchaze ukuba "Ndiyavumelana ngamandla", "Ndiyavumelana", "Phakathi, "Andivumelani", or "Andivumelani ngamandlla" ngazinye ezingxelo zilandelayo.

We are now going to ask you questions about your experience of access to health facilities and the care you received from these health facilities. In the following questions, except where explicitly asked otherwise, respond with ONE of the following options: strongly agree, agree, neutral, disagree or strongly disagree.

Ons gaan jou nou vrae vra oor jou ervaring met toegang tot gesondheidsfasiliteite en die versorging wat jy van hierdie gesondheidsorgfasiliteite ontvang het. Beantwoord die volgende vrae met EEN van die volgende opsies: stem heeltemal saam, stem saam, neutral, stem nie saam nie or stem heeltemal nie saam nie.

◯ Next
(Read to the participant)

Kuye kufuneka ndilinde ixesha elide kwiziko lonyango (kliniki) phambi kokuba Ndifumane amayeza wam we TB.

I have to wait a long time at the health facility (clinic) before I receive my TB medications.

Ek moet lank in die gesondheidsentrum wag voordat ek my TB-medikasie ontvang

◯ Uyavumelana ngamandla # Strongly agree # Stem heeltemal saam
◯ Uyavumelana # Agree # Stem saam
◯ Phakathi # Neutral # Neutraal
◯ Awuvumelani # Disagree # Stem nie saam nie
◯ Awuvumelani ngamandla # Strongly disagree # Stem geheel en al nie saam nie
(Read to the participant; do not read the response options aloud)

Mingaphi imizuzu oqhele ukuyilinda phambi kokuba unikwe amayeza wakho?

How many minutes do you normally have to wait before you are given your medications?

Hoeveel minute moet jy normaalweg wag voordat jy jou medikasie ontvang?

◯ < kwemizuzu eyi 5 # < 5 minutes # < 5 minute
◯ 5 - 15 imizuzu # 5 - 15 minutes # 5 - 15 minute
◯ 15-30 imizuzu # 15 - 30 minutes # 15 - 30 minute
◯ 30 imizuzu - kwi yure eyi 1 # 30 minutes - 1 hour # 30 minute - 1 uur
◯ > iyure eziyi 2 # >2 hours # >2 ure
(Ask the participant; and read the response options aloud)

Umsebenzi wezempilo kwinqubo yeze Sifo sephepha usoloko endiphatha ngembeko/ntlonipho.

The health worker at the TB program always treats me with respect.

Die gesondheidswerker by die TB-program behandel my altyd met respek.

◯ Uyavumelana ngamandla # Strongly agree # Stem heeltemal saam
◯ Uyavumelana # Agree # Stem saam
◯ Phakathi # Neutral # Neutraal
◯ Awuvumelani # Disagree # Stem nie saam nie
◯ Awuvumelani ngamandla # Strongly disagree # Stem geheel en al nie saam nie
(Ask the participant; and read the response options aloud)

For peer review only

Ngokwesiqhelo, Ndiyathanda indlela endiphathwa ngayo kwinkqubo yesifo sephepha kwiziko lwezonyango (kliniki).<br><br>Normally, I like how I am treated at the TB program at the health facility (clinic).<br><br>Normaalweg hou ek van hoe ek behandel word tydens die TB-program by die gesondheidsfasiliteit (kliniek).	◯ Uyavumelana ngamandla # Strongly agree # Stem heeltemal saam<br>◯ Uyavumelana # Agree # Stem saam<br>◯ Phakathi # Neutral # Neutraal<br>◯ Awuvumelani # Disagree # Stem nie saam nie<br>◯ Awuvumelani ngamandla # Strongly disagree # Stem geheel en al nie saam nie<br>(Read to the participant; do not read the response options aloud)
Ababoneleli kwiziko lwezempilo baye bandicacisela kakuhle ukuba yintoni isifo sephepha kwaye lunjani unyango lwayo.<br><br>The providers at the health center have clearly explained to me what TB is and how to use the treatment.<br><br>Die diensverskaffers by die gesondheidsentrum het my mooi verduidelik wat TB is en hoe dit is om die behandeling te gebruik.	◯ Uyavumelana ngamandla # Strongly agree # Stem heeltemal saam<br>◯ Uyavumelana # Agree # Stem saam<br>◯ Phakathi # Neutral # Neutraal<br>◯ Awuvumelani # Disagree # Stem nie saam nie<br>◯ Awuvumelani ngamandla # Strongly disagree # Stem geheel en al nie saam nie<br>(Read to the participant; do not read the response options aloud)
Indawo ebonakalayo eyenkqubo yeSifo sephepha kwiziko lwezempilo ifanelekile ukunceda abantu abafikisayo.<br><br>The physical space of the TB program at the health center is comfortable for adolescents.<br><br>Die fisiese ruimte van die TB-program by die gesondheidsentrum is gemaklik vir adolessente.	◯ Uyavumelana ngamandla # Strongly agree # Stem heeltemal saam<br>◯ Uyavumelana # Agree # Stem saam<br>◯ Phakathi # Neutral # Neutraal<br>◯ Awuvumelani # Disagree # Stem nie saam nie<br>◯ Awuvumelani ngamandla # Strongly disagree # Stem geheel en al nie saam nie<br>(Read to the participant; do not read the response options aloud)
Ababoneleli kwinkqubo yeSifo sephepha bakukhathalele ukunyangeka kwam/ ukuphila kwam.<br><br>The providers at the TB program care about my recovery.<br><br>Die diensverskaffers van die TB-program gee om vir my herstel.	◯ Uyavumelana ngamandla # Strongly agree # Stem heeltemal saam<br>◯ Uyavumelana # Agree # Stem saam<br>◯ Phakathi # Neutral # Neutraal<br>◯ Awuvumelani # Disagree # Stem nie saam nie<br>◯ Awuvumelani ngamandla # Strongly disagree # Stem geheel en al nie saam nie<br>(Read to the participant; do not read the response options aloud)
Ababoneleli bempilo kwiziko lwezempilo bayandinika into yokusela kunye namayeza wam (amanzi, ijusi, nokunye...)<br><br>The health providers at the health facility (clinic) give me something to drink with my medications (water, juice, etc.)<br><br>Die gesondheidsaanbieders by die gesondheidsinstansie (kliniek) gee my iets om te drink saam met my medikasie (water, sap, ens.)	◯ Uyavumelana ngamandla # Strongly agree # Stem heeltemal saam<br>◯ Uyavumelana # Agree # Stem saam<br>◯ Phakathi # Neutral # Neutraal<br>◯ Awuvumelani # Disagree # Stem nie saam nie<br>◯ Awuvumelani ngamandla # Strongly disagree # Stem geheel en al nie saam nie<br>(Read to the participant; do not read the response options aloud)

For peer review only

Ababoneleli kwiziko lwempilo bayandijonga xandithatha amayeza wam.<br><br>The providers at the health facility (clinic) observe me when I take my medications.<br><br>Die verskaffers van die gesondheidsfasiliteit (kliniek) neem my waar as ek my medisyne gebruik.	◯ Uyavumelana ngamandla # Strongly agree # Stem heeltemal saam<br>◯ Uyavumelana # Agree # Stem saam<br>◯ Phakathi # Neutral # Neutraal<br>◯ Awuvumelani # Disagree # Stem nie saam nie<br>◯ Awuvumelani ngamandla # Strongly disagree # Stem geheel en al nie saam nie<br>(Read to the participant; do not read the response options aloud)
Ndonwabile nge shedyuli kwaye nomgangatho wenkathalo kwinkqubo yeSifo sephepha.<br><br>I am happy with the schedule and quality of care at the TB program.<br><br>Ek is tevrede met die skedule en kwaliteit van die sorg by die TB-program.	◯ Uyavumelana ngamandla # Strongly agree # Stem heeltemal saam<br>◯ Uyavumelana # Agree # Stem saam<br>◯ Phakathi # Neutral # Neutraal<br>◯ Awuvumelani # Disagree # Stem nie saam nie<br>◯ Awuvumelani ngamandla # Strongly disagree # Stem geheel en al nie saam nie<br>(Read to the participant; do not read the response options aloud)

Motivation

Ngoku sizokubuza imibuzo ngokuba ukhuthazeke kangakanani/njani malunga nokuthatha kwaye ukugqiba amayeza onyango. Njengakuqala, uyacelwa uchaze ukuba "Ndiyavumelana ngamandla", "Ndiyavumelana", "Phakathi, "Andivumelani", or "Andivumelani ngamandlla" ngazinye ezingxelo zilandelayo.<br><br>We are now going to ask you questions about how motivated you feel about taking and completing your treatment. In the following questions, except where explicitly asked otherwise, respond with ONE of the following options: strongly agree, agree, neutral, disagree or strongly disagree.<br><br>Ons gaan jou nou vrae vra oor hoe gemotiveerd jy voel om jou behandeling te neem en te voltooi. Beantwoord die volgende vrae met EEN van die volgende opsies: stem heeltemal saam, stem saam, neutral, stem nie saam nie or stem heeltemal nie saam nie.	◯ Next<br>(Read to the participant)
Ndifuna ukugqiba unyango lwam ngokukhawulezileyo ukuzw ndingosuleli amalungu wosapho lwam.<br><br>I want to finish my treatment as soon as possible so as not to infect my family members.<br><br>Ek wil my behandeling so gou as moontlik voltooi, sodat ek nie my familielede aansteek nie.	◯ Uyavumelana ngamandla # Strongly agree # Stem heeltemal saam<br>◯ Uyavumelana # Agree # Stem saam<br>◯ Phakathi # Neutral # Neutraal<br>◯ Awuvumelani # Disagree # Stem nie saam nie<br>◯ Awuvumelani ngamandla # Strongly disagree # Stem geheel en al nie saam nie<br>(Read to the participant; do not read the response options aloud)
Ndifuna ukugqiba unyango lwam ngokukhawulezileyo ukuze usapho lwam lungaphinde luxhalabe ngam.<br><br>I want to finish my treatment as soon as possible so my family no longer has to worry about me.<br><br>Ek wil my behandeling so gou as moontlik voltooi, sodat my gesin nie meer oor my hoef te bekommer nie.	◯ Uyavumelana ngamandla # Strongly agree # Stem heeltemal saam<br>◯ Uyavumelana # Agree # Stem saam<br>◯ Phakathi # Neutral # Neutraal<br>◯ Awuvumelani # Disagree # Stem nie saam nie<br>◯ Awuvumelani ngamandla # Strongly disagree # Stem geheel en al nie saam nie<br>(Read to the participant; do not read the response options aloud)

For peer review only

Ndifuna ukugqiba unyango lwam ngokukhawulezileyo ukuze ndikwazi ukuqhubeka nezifundo zam okanye umsebenzi.<br><br>I want to finish my treatment as soon as possible so I can continue my studies or work.<br><br>Ek wil my behandeling so gou as moontlik voltooi, sodat ek kan voortgaan met my studies of werk.	◯ Uyavumelana ngamandla # Strongly agree # Stem heeltemal saam<br>◯ Uyavumelana # Agree # Stem saam<br>◯ Phakathi # Neutral # Neutraal<br>◯ Awuvumelani # Disagree # Stem nie saam nie<br>◯ Awuvumelani ngamandla # Strongly disagree # Stem geheel en al nie saam nie<br>(Read to the participant; do not read the response options aloud)
Ndifuna ukugqiba unyango lwam ngokukhawulezileyo ukuze ndibuyele kwisiqhelo sam, kwizinto zam ebendizenza (ukuphuma nabahlobo, ukudlala ibhola, ukudanisa, ukuyotyhibiliza, njalo njalo)<br><br>I want to finish my treatment as soon as possible so I can return to my normal, personal activities (go out with friends, play soccer, dance, go skating, etc.)<br><br>Ek wil my behandeling so gou as moontlik voltooi, sodat ek weer na my normale persoonlike aktiwiteite kan terugkeer (saam met vriende uitgaan, sokker speel, dans, skaats, ens.)	◯ Uyavumelana ngamandla # Strongly agree # Stem heeltemal saam<br>◯ Uyavumelana # Agree # Stem saam<br>◯ Phakathi # Neutral # Neutraal<br>◯ Awuvumelani # Disagree # Stem nie saam nie<br>◯ Awuvumelani ngamandla # Strongly disagree # Stem geheel en al nie saam nie<br>(Read to the participant; do not read the response options aloud)
Ndinexhala ukuba kuzonyanzeleka ndiphinde unyaka eskolweni okanye ezinye zezifundo zam ngenxa yesigulo sam.<br><br>I am worried that I will have to repeat a school year or some of my studies because of my illness.<br><br>Ek is bekommerd dat ek 'n skooljaar of 'n deel van my studies sal moet herhaal as gevolg van my siekte.	◯ Uyavumelana ngamandla # Strongly agree # Stem heeltemal saam<br>◯ Uyavumelana # Agree # Stem saam<br>◯ Phakathi # Neutral # Neutraal<br>◯ Awuvumelani # Disagree # Stem nie saam nie<br>◯ Awuvumelani ngamandla # Strongly disagree # Stem geheel en al nie saam nie<br>(Read to the participant; do not read the response options aloud)

Knowledge about TB

Ngoku sizokubuza imibuzo malunga nokuqonda kwakho isifo sephepha.<br><br>We are now going to ask you questions about your understanding of TB.<br><br>Ons gaan jou nou vrae vra oor jou begrip/kennis van TB.	◯ Next<br>(Read to the participant)
Isifo sephepha singanyangeka kuphele.<br><br>TB can be completely cured.<br><br>TB kan volkome genees word.	◯ Uyavumelana ngamandla # Strongly agree # Stem heeltemal saam<br>◯ Uyavumelana # Agree # Stem saam<br>◯ Phakathi # Neutral # Neutraal<br>◯ Awuvumelani # Disagree # Stem nie saam nie<br>◯ Awuvumelani ngamandla # Strongly disagree # Stem geheel en al nie saam nie<br>(Read to the participant; do not read the response options aloud)

For peer review only

Sigqithiswa/Sisulela njani isifo sephepha (Khetha konke okufanelekileyo)<br><br>How is TB transmitted?<br><br>Hoe word TB oorgedra? (kies ALLES wat van toepassing is)	☐ Ngokobelana ngesondo # Through sexual relations # Deur seksuele verhoudings<br>☐ Ngokokusebenzisa izitya namacaphe amanye # Through plates and cutlery # Deur borde en eetgerei<br>☐ Ngokwempahla nangetawuli # Through clothing sheets and towels # Deur kleredoeke en handdoeke<br>☐ Ngokuncamisana # Through kissing # Deur soen<br>☐ Ngoku wonga # Through hugging # Deur drukkies<br>☐ Andazi # I don't know # Ek weet nie<br>(Ask the participant; and read the response options aloud)
Ukuba ndithe ndaphosa ezinye iintsuku zonyango lwam lwesifo sephepha, Isifo sam sephepha singabuya "ngamandla"<br><br>If I miss some days of my TB treatment, my TB could come back "worse."<br><br>As ek 'n paar dae van my TB-behandeling mis, kan my TB "erger" word.	◯ Uyavumelana ngamandla # Strongly agree # Stem heeltemal saam<br>◯ Uyavumelana # Agree # Stem saam<br>◯ Phakathi # Neutral # Neutraal<br>◯ Awuvumelani # Disagree # Stem nie saam nie<br>◯ Awuvumelani ngamandla # Strongly disagree # Stem geheel en al nie saam nie<br>(Ask the participant; and read the response options aloud)
Ukuba ndiyeke kwaphela ukuthatha unyango lwam lwesifo sephepha, Isifo sephepha sam sobuya "ngamandla".<br><br>If I completely stop taking my TB treatment, my TB could come back "worse."<br><br>As ek my TB-behandeling heeltemal ophou, kan my TB 'erger' wees.	◯ Uyavumelana ngamandla # Strongly agree # Stem heeltemal saam<br>◯ Uyavumelana # Agree # Stem saam<br>◯ Phakathi # Neutral # Neutraal<br>◯ Awuvumelani # Disagree # Stem nie saam nie<br>◯ Awuvumelani ngamandla # Strongly disagree # Stem geheel en al nie saam nie<br>(Read to the participant; do not read the response options aloud)
Ndiyakholwa ukuba ndifumana ulwazi/inkcukacha ezaneleyo malunga nesifo sephepha kwiziko lwezempilo.<br><br>I believe that I have received enough information about TB from the health centre.<br><br>Ek glo dat ek genoeg inligting oor TB van die gesondheidsentrum ontvang het.	◯ Uyavumelana ngamandla # Strongly agree # Stem heeltemal saam<br>◯ Uyavumelana # Agree # Stem saam<br>◯ Phakathi # Neutral # Neutraal<br>◯ Awuvumelani # Disagree # Stem nie saam nie<br>◯ Awuvumelani ngamandla # Strongly disagree # Stem geheel en al nie saam nie<br>(Read to the participant; do not read the response options aloud)
Ndineemibuzo okanye iintandabuzo ezongezekileyo malunga nesifo sephepha endingathanda zicaciswe kum.<br><br>I have more questions or doubts about TB that I would like to be explained to me.<br><br>Ek het meer vrae of onsekerhede oor TB wat ek aan my wil verduidelik hê.	◯ Uyavumelana ngamandla # Strongly agree # Stem heeltemal saam<br>◯ Uyavumelana # Agree # Stem saam<br>◯ Phakathi # Neutral # Neutraal<br>◯ Awuvumelani # Disagree # Stem nie saam nie<br>◯ Awuvumelani ngamandla # Strongly disagree # Stem geheel en al nie saam nie<br>(Read to the participant; do not read the response options aloud)

For peer review only

Ndingakhetha iinkcukacha/ulwazi malunga nesifo sephepha lusuke ku/kwi (khetha Konke okufanelekileyo)<br><br>I would prefer information about TB to come from (select ALL that apply)<br><br>Ek sal verkies dat ek inligting kry oor TB van (kies ALLES wat van toepassing is).	☐ Abazali bam # My parents # My ouers<br>☐ Iintetha esikolweni/ dyunivesithi # Talks at school/university # Gesprekke op skool / universiteit<br>☐ Abanye abantu abafikisayo nolutsha olwakhe lwane TB # Other adolescents and youth who have had TB # Ander adolessente en jeugdiges wat TB gehad het<br>☐ "iminxeba yengcebiso" efanele abantu abafikisayo abane TB # TB "advice lines" exclusively for adolescents # "TB-advieslyne" uitsluitlik vir adolessente<br>☐ Intanethi # Internet # Internet<br>☐ Omnye # Other # Ander<br>(Read to the participant; do not read the response options aloud)
Ndingakhetha iinkcukacha/ulwazi malunga nesifo sephepha lusuke ku/kwi. Uyacelwa ucacise.<br><br>I would prefer information about TB to come from. Please specify.<br><br>Ek sou verkies dat inligting oor TB vandaan kom. Spesifiseer asseblief.	____________________<br>(If Other, specify)
Kwimibuzo elandelayo, uyacelwa uphendule ngenye yezimpendulo zilandelayo: ngamaxesha onke, ngamaxesha amaninzi, ngamaxesha athile, manqaphanqapha okanye zange/soze.<br><br>In the following questions, please respond with ONE of the following options: strongly agree, agree, neutral, disagree or strongly disagree.<br><br>Beantwoord die volgende vrae met EEN van die volgende opsies: stem heeltemal saam, stem saam, neutral, stem nie saam nie or stem heeltemal nie saam nie.	◯ Next<br>(Read to the participant)
Ndakhe ndava ngesifo sephepha phambi kokuba ndigule.<br><br>I heard about TB before I got sick with TB.<br><br>Ek het van TB gehoor voordat ek siek geword het met TB.	◯ Uyavumelana ngamandla # Strongly agree # Stem heeltemal saam<br>◯ Uyavumelana # Agree # Stem saam<br>◯ Phakathi # Neutral # Neutraal<br>◯ Awuvumelani # Disagree # Stem nie saam nie<br>◯ Awuvumelani ngamandla # Strongly disagree # Stem geheel en al nie saam nie<br>(Read to the participant; do not read the response options aloud)
Bendikhe ndakhangela ulwazi malunga nesifo sephepha kwi Intanethi.<br><br>I have searched for information about TB on the internet.<br><br>Ek het op die internet na inligting oor TB gesoek.	◯ Uyavumelana ngamandla # Strongly agree # Stem heeltemal saam<br>◯ Uyavumelana # Agree # Stem saam<br>◯ Phakathi # Neutral # Neutraal<br>◯ Awuvumelani # Disagree # Stem nie saam nie<br>◯ Awuvumelani ngamandla # Strongly disagree # Stem geheel en al nie saam nie<br>(Read to the participant; do not read the response options aloud)

For peer review only

How do I feel about having TB	
Ngoku sizokubuza imibuzo malunga nolwazi lwakho ngesifo sephepha.<br><br>We are now going to ask you questions about your understanding of TB.<br><br>Ons gaan jou nou vrae vra oor jou begrip/kennis van TB.	◯ Next<br>(Read to the participant)
Ndiyoyika ukuxelela usapho lwam ukuba ndinesifo sephepha.<br><br>I am afraid to tell my family members that I have TB.<br><br>Ek is bang om my familielede te vertel dat ek TB het.	◯ Maxesha wonke # Always # Altyd<br>◯ Maxesha amaninzi # Often # Gereeld<br>◯ Ngamanye amaxesha # Sometimes # Soms<br>◯ Manqaphanqapha # Rarely # Selde<br>◯ Zange/soze # Never # Nooit nie<br>(Ask the participant; and read the response options aloud)
Ndiyoyika ukuya kwiziko lwezempilo ukuyothatha iipilisi zam ngenxa ndisoyika ukuba abanye bazondibona.<br><br>I am afraid to go to the health center to get my pills because I am afraid others will see me.<br><br>Ek is bang om na die gesondheidsentrum te gaan om my pille te kry, want ek is bang ander mense sal my sien.	◯ Maxesha wonke # Always # Altyd<br>◯ Maxesha amaninzi # Often # Gereeld<br>◯ Ngamanye amaxesha # Sometimes # Soms<br>◯ Manqaphanqapha # Rarely # Selde<br>◯ Zange/soze # Never # Nooit nie<br>(Read to the participant; do not read the response options aloud)
Ndiyoyika ukuxelela abantu ukuba ndineSifo sephepha ngoba bangacinga ukuba ndinesifo sika Gawulayo.<br><br>I am afraid to tell people that I have TB because they might think that I also have AIDS.<br><br>Ek is bang om vir mense te sê dat ek TB het, omdat hulle sal dink dat ek ook VIGS het.	◯ Maxesha wonke # Always # Altyd<br>◯ Maxesha amaninzi # Often # Gereeld<br>◯ Ngamanye amaxesha # Sometimes # Soms<br>◯ Manqaphanqapha # Rarely # Selde<br>◯ Zange/soze # Never # Nooit nie<br>(Read to the participant; do not read the response options aloud)
Ndiziva ndinexhala ngoba ndicinga ndagula Sisifo sephepha ngenxa yokungalandeli indlela esempilweni yokutya.<br><br>I feel guilty because I think I got sick with TB because I did not follow a healthy diet.<br><br>Ek voel skuldig omdat ek dink dat ek TB gekry het omdat ek nie 'n gesonde dieet gevolg het nie.	◯ Maxesha wonke # Always # Altyd<br>◯ Maxesha amaninzi # Often # Gereeld<br>◯ Ngamanye amaxesha # Sometimes # Soms<br>◯ Manqaphanqapha # Rarely # Selde<br>◯ Zange/soze # Never # Nooit nie<br>(Read to the participant; do not read the response options aloud)
Ndiziva ndinesazela ngenxa yokuba ndicinga ndagula yi TB ngenxa yokutshaya, ukusela utywala, okanye ukusebenzisa ezinye iziyobisi.<br><br>I feel guilty because I think I got sick with TB because of smoking, drinking alcohol, or using other drugs.<br><br>Ek voel skuldig omdat ek dink dat ek siek geword het van TB as gevolg van rook, alkohol drink of ander dwelms gebruik het.	◯ Maxesha wonke # Always # Altyd<br>◯ Maxesha amaninzi # Often # Gereeld<br>◯ Ngamanye amaxesha # Sometimes # Soms<br>◯ Manqaphanqapha # Rarely # Selde<br>◯ Zange/soze # Never # Nooit nie<br>(Read to the participant; do not read the response options aloud)

For peer review only

Ndiziva ndinesazela ngokufumana i TB ngoba ndingumthwalo kusapho lwam.<br><br>I feel guilty about getting TB because I am a burden for my family.<br><br>Ek voel skuldig omdat ek TB het, want dit is 'n las vir my gesin.	◯ Maxesha wonke # Always # Altyd<br>◯ Maxesha amaninzi # Often # Gereeld<br>◯ Ngamanye amaxesha # Sometimes # Soms<br>◯ Manqaphanqapha # Rarely # Selde<br>◯ Zange/soze # Never # Nooit nie<br>(Read to the participant; do not read the response options aloud)
Ndilumnkile xa ndikhetha umntu endithetha naye malunga nesigulo same se TB.<br><br>I am careful when choosing whom to speak to about my TB.<br><br>Ek is versigtig wanneer ek moet kies met wie om te praat oor my TB.	◯ Maxesha wonke # Always # Altyd<br>◯ Maxesha amaninzi # Often # Gereeld<br>◯ Ngamanye amaxesha # Sometimes # Soms<br>◯ Manqaphanqapha # Rarely # Selde<br>◯ Zange/soze # Never # Nooit nie<br>(Read to the participant; do not read the response options aloud)
Ndingaphulukana nabahlobo bam ukuba ndiyabachazela ndine TB.<br><br>I might lose my friends if I tell them I have TB.<br><br>Ek kan my vriende verloor as ek vir hulle sê dat ek TB het.	◯ Maxesha wonke # Always # Altyd<br>◯ Maxesha amaninzi # Often # Gereeld<br>◯ Ngamanye amaxesha # Sometimes # Soms<br>◯ Manqaphanqapha # Rarely # Selde<br>◯ Zange/soze # Never # Nooit nie<br>(Read to the participant; do not read the response options aloud)
Ndiyoyika ukuxelela abantu abangomalungu osapho ukuba ndine TB.<br><br>I am afraid to tell people who are not family members that I have TB.<br><br>Ek is bang om mense wat nie familielede is nie, te vertel dat ek TB het.	◯ Maxesha wonke # Always # Altyd<br>◯ Maxesha amaninzi # Often # Gereeld<br>◯ Ngamanye amaxesha # Sometimes # Soms<br>◯ Manqaphanqapha # Rarely # Selde<br>◯ Zange/soze # Never # Nooit nie<br>(Read to the participant; do not read the response options aloud)
Ndiziva ndindedwa ngengxa yesisigulo sam se TB.<br><br>I feel alone because of my TB.<br><br>Ek voel alleen as gevolg van my TB.	◯ Maxesha wonke # Always # Altyd<br>◯ Maxesha amaninzi # Often # Gereeld<br>◯ Ngamanye amaxesha # Sometimes # Soms<br>◯ Manqaphanqapha # Rarely # Selde<br>◯ Zange/soze # Never # Nooit nie<br>(Read to the participant; do not read the response options aloud)
Ndiziva ndinomsindo ngendlela abantu abothuka ngayo xabefumanisa ukuba ndine TB.<br><br>I feel upset by the way people react when they find out that I have TB.<br><br>Ek voel ontsteld oor die manier waarop mense reageer as hulle uitvind dat ek TB het.	◯ Maxesha wonke # Always # Altyd<br>◯ Maxesha amaninzi # Often # Gereeld<br>◯ Ngamanye amaxesha # Sometimes # Soms<br>◯ Manqaphanqapha # Rarely # Selde<br>◯ Zange/soze # Never # Nooit nie<br>(Read to the participant; do not read the response options aloud)
Ndiye ndigcine umgama kwabanye abantu ukuze ndinqande ukuba ndigqithise i TB yam kubo.<br><br>I keep my distance from others to avoid passing on my TB to them.<br><br>Ek hou my afstand van ander sodat ek nie my TB aan hulle oordra nie.	◯ Maxesha wonke # Always # Altyd<br>◯ Maxesha amaninzi # Often # Gereeld<br>◯ Ngamanye amaxesha # Sometimes # Soms<br>◯ Manqaphanqapha # Rarely # Selde<br>◯ Zange/soze # Never # Nooit nie<br>(Read to the participant; do not read the response options aloud)

For peer review only

Mood and Emotions

Imibuzo elandelayo imalunga nemvakalelo zakho. Kukangakanani/ kukangaphi apho wathi wava enye yezimpawu zilandelayo kweziveki zimbini zigqithileyo/zidlulileyo? Ngampawo ngaye, khetha eyona ndlela ecacisa ngokupheleleyo ukuba uye waziva njani.<br><br>The following questions are about your feelings and emotions. How often have you felt each of the following symptoms during the PAST TWO WEEKS? For every symptom, select the option that best describes how you have felt.<br><br>Die volgende vrae handel oor jou gevoelens en emosies. Hoe gereeld het jy elkeen van die volgende simptome ervaar gedurende die AFGELOPE TWEE WEKE? Kies vir elke simptoom die opsie wat die beste beskryf hoe jy gevoel het.	◯ Next<br>(Read to the participant)
Ubukhe waziva unomoya ophantsi, ukhathazekile, udinekile, okanye uphelelwe lithemba?<br><br>Have you ever felt down, depressed, irritable, or hopeless?<br><br>Het jy al ooit af, depressief, geïrriteerd of hopeloos gevoel?	◯ Zange/ soze # Never # Nooit nie<br>◯ Ngezinye iintsuku # Some days # Sommige dae<br>◯ Ngapha kwe hafu yeentsuku # More than half the days # Meer as die helfte van die dae<br>◯ Kangangemihla yonke # Almost every day # Byna elke dag<br>(Ask the participant; and read the response options aloud)
Ukhe waziva unomdla omncinci okanye ungonwabanga ekwenzeni izinto?<br><br>Have you felt little interest or pleasure in doing things?<br><br>Het jy min belangstelling of plesier gehad om dinge te doen?	◯ Zange/ soze # Never # Nooit nie<br>◯ Ngezinye iintsuku # Some days # Sommige dae<br>◯ Ngapha kwe hafu yeentsuku # More than half the days # Meer as die helfte van die dae<br>◯ Kangangemihla yonke # Almost every day # Byna elke dag<br>(Read to the participant; do not read the response options aloud)
Ubukhe wanengxaki yokunzinyelwa kulala okanye ungakwazi ulala ithuba elide?<br><br>Have you had problems falling asleep or staying asleep?<br><br>Het jy probleme gehad om aan die slaap te raak of aan die slaap te bly?	◯ Zange/ soze # Never # Nooit nie<br>◯ Ngezinye iintsuku # Some days # Sommige dae<br>◯ Ngapha kwe hafu yeentsuku # More than half the days # Meer as die helfte van die dae<br>◯ Kangangemihla yonke # Almost every day # Byna elke dag<br>(Read to the participant; do not read the response options aloud)
Okanye ngokuchaseneyo, ubukhe waziva ulala ngokugqithisileyo?<br><br>Or by contrast, have you been sleeping too much?<br><br>Of inteendeel, het jy te veel geslaap?	◯ Zange/ soze # Never # Nooit nie<br>◯ Ngezinye iintsuku # Some days # Sommige dae<br>◯ Ngapha kwe hafu yeentsuku # More than half the days # Meer as die helfte van die dae<br>◯ Kangangemihla yonke # Almost every day # Byna elke dag<br>(Read to the participant; do not read the response options aloud)

For peer review only

Ubukhe waziva udiniwe kancinci okanye unamandla amancinci?<br><br>Have you ever felt tired or with little energy?<br><br>Het jy al ooit moeg gevoel of met min energie?	◯ Zange/ soze # Never # Nooit nie<br>◯ Ngezinye iintsuku # Some days # Sommige dae<br>◯ Ngapha kwe hafu yeentsuku # More than half the days # Meer as die helfte van die dae<br>◯ Kangangemihla yonke # Almost every day # Byna elke dag<br>(Read to the participant; do not read the response options aloud)
Ubukhe wahlelwa kukucacela ukutya okanye wahla emzimbeni?<br><br>Have you had a lowered appetite, or have you lost weight?<br><br>Het jy 'n swakker eetlus gehad of het jy gewig verloor?	◯ Zange/ soze # Never # Nooit nie<br>◯ Ngezinye iintsuku # Some days # Sommige dae<br>◯ Ngapha kwe hafu yeentsuku # More than half the days # Meer as die helfte van die dae<br>◯ Kangangemihla yonke # Almost every day # Byna elke dag<br>(Read to the participant; do not read the response options aloud)
Okanye ngokuchaseneyo, ukhe wazibona usitya ngokugqithisileyo?<br><br>Or, by contrast, have you eaten excessively?<br><br>Of inteendeel, het jy oormatig/te veel geëet?	◯ Maxesha wonke # Always # Altyd<br>◯ Maxesha amaninzi # Often # Dikwels<br>◯ Ngamanye amaxesha # Sometimes # Soms<br>◯ Manqapha nqapha # Rarely # Selde<br>(Ask the participant; and read the response options aloud)
Ubukhe waziva ukhathazekile ngesiqu sakho okanye wanengcinga zokuzibona njengomntu owohlulekileyo, okanye njengamntu othe waziphoxa okanye usapho lwakho?<br><br>Have you felt bad about yourself or have you thought of yourself as a failure, or someone who has disappointed yourself or your family?<br><br>Het jy sleg gevoel oor jouself of het jy aan jouself gedink as 'n mislukking, of soos iemand wat jouself of jou gesin teleurgestel het?	◯ Maxesha wonke # Always # Altyd<br>◯ Maxesha amaninzi # Often # Dikwels<br>◯ Ngamanye amaxesha # Sometimes # Soms<br>◯ Manqapha nqapha # Rarely # Selde<br>(Ask the participant; and read the response options aloud)
Ubukhe wanzinyelwa ekukwenzeni imisebenzi ethile, efana nomsebenzi wesikolo, ukufunda, okanye ukubukela umabonakude?<br><br>Have you had difficulties concentrating in certain activities, like schoolwork, reading, or watching television?<br><br>Het jy probleme gehad om op sekere aktiwiteite te konsentreer, soos skoolwerk, lees of televisie kyk?	◯ Maxesha wonke # Always # Altyd<br>◯ Maxesha amaninzi # Often # Dikwels<br>◯ Ngamanye amaxesha # Sometimes # Soms<br>◯ Manqapha nqapha # Rarely # Selde<br>(Read to the participant; do not read the response options aloud)
Ubukhe wacotha ukuhamba okanye ukuthetha bade abanye abantu bakuqaphela oko?<br><br>Have you ever moved or talked so slowly that other people have taken notice?<br><br>Het jy al ooit so stadig beweeg of gepraat dat ander mense opgelet het?	◯ Zange/ soze # Never # Nooit nie<br>◯ Ngezinye iintsuku # Some days # Sommige dae<br>◯ Ngapha kwe hafu yeentsuku # More than half the days # Meer as die helfte van die dae<br>◯ Kangangemihla yonke # Almost every day # Byna elke dag<br>(Ask the participant; and read the response options aloud)

For peer review only

Okanye, ngokuchaseneyo, ubukhe awafumana ukuphumla kwade awakwazi ukuyeka ukuhamba ukodlula isiqhelo?<br><br>Or, by contrast, have you been so restless that you haven't been able to stop moving more than usual?<br><br>Of inteendeel, was jy so onrustig dat jy, meer as gewoonklik, nie kon ophou beweeg nie?	◯ Zange/ soze # Never # Nooit nie<br>◯ Ngezinye iintsuku # Some days # Sommige dae<br>◯ Ngapha kwe hafu yeentsuku # More than half the days # Meer as die helfte van die dae<br>◯ Kangangemihla yonke # Almost every day # Byna elke dag<br>(Ask the participant; and read the response options aloud)
Wakhe wacinga ukuba kungangcono uba ufe okanye wakhe wacinga ukuzilimaza ngendlela ethile?<br><br>Have you ever thought that it would be better to be dead or have you thought of hurting yourself in some way?<br><br>Het jy al ooit gedink dat dit beter sou wees om dood te wees, of het jy gedink om jouself op een of ander manier te beseer?	◯ Zange/ soze # Never # Nooit nie<br>◯ Ngezinye iintsuku # Some days # Sommige dae<br>◯ Ngapha kwe hafu yeentsuku # More than half the days # Meer as die helfte van die dae<br>◯ Kangangemihla yonke # Almost every day # Byna elke dag<br>(Ask the participant; and read the response options aloud)
**Alcohol Consumption**	
Kubalulekile ukuba sikubuze imibuzo ethile malunga nendlela osela ngayo utywala. Iimpendulo zakho zizakuba zemfihlakalweni, ngoko uyacelwa unyaniseke. Iimpendulo zakho kumele zigxile kwendlela yoziphatha kwakho kwezinyanga ziyi 12 zidlulileyo:<br><br>It is important that we ask you certain questions about your consumption of alcohol. Your responses will be confidential, so please be honest. Your answers should be in response to your behavior over the past 12 months:<br><br>Dit is belangrik dat ons sekere vrae vra oor jou alkoholverbruik. Jou antwoorde sal vertroulik wees, dus wees eerlik. Jou antwoorde moet reageer op jou gedrag die afgelope 12 maande:	◯ Next<br>(Read to the participant)
Usela kangaphi/ kangakanani iziselo zotywala?<br><br>How often do you drink alcoholic beverages?<br><br>Hoe gereeld drink jy alkoholiese drankies?	◯ Zange/ soze # Never # Nooit nie<br>◯ Kanye okanye ngaphantsi ngenya # Once or less per month # Een of minder keer per maand<br>◯ Ukusuka kumaxesha ayi 2 - 4 ngenyanga # From 2-4 times per month # Van 2-4 keer per maand<br>◯ Ukusuka kumaxesha ayi 2- kwayi 3 nge veki # From 2-3 times per week # Van 2-3 keer per week<br>◯ 4 okanye ngaphezulu nge veki # 4 or more times per week # 4 of meer keer per week<br>(Ask the participant; and read the response options aloud)
Zingaphi iziselo zotywala oziselayo ngokwesiqhelo nje kwimini ethile?<br><br>How many alcoholic drinks do you normally have on a typical day?<br><br>Hoeveel alkoholohiese drankies drink jy gewoonlik op 'n tipiese dag?	◯ 1 ukuya ku 2 # 1 to 2 # 1 tot 2<br>◯ 2 ukuya ku 4 # 2 to 4 # 2 tot 4<br>◯ 5 ukuya ku 6 # 5 to 6 # 5 tot 6<br>◯ 7 ukuya ku 9 # 7 to 9 # 7 tot 9<br>◯ 10 okanye ngaphezulu # 10 or more # 10 of meer<br>(Ask the participant; and read the response options aloud)

For peer review only

Kukangaphi apho usela iziselo zotywala eziyi 6 okanye nangaphezulu ngosuku olunye?<br><br>How often do you have 6 or more alcoholic beverages in only one day?<br><br>Hoe gereeld drink jy 6 of meer alkoholiese drankies in een dag?	◯ Zange/ soze # Never # Nooit nie<br>◯ Ngenyanga # Monthly # Maandeliks<br>◯ Ngeveki # Weekly # Weekliks<br>◯ Phantse mihla yonke # Almost daily # Byna daagliks<br>◯ Mihla yonke # Daily # Daagliks<br>(Ask the participant; and read the response options aloud)
Kukangaphi kunyaka ogqithileyo ubukhe awakwazi ukuyeka ukusela ekubeni uthe waqala?<br><br>How often in the past year have you been unable to stop drinking once you started?<br><br>Hoe gereeld het jy die afgelope jaar nie kon ophou drink sodra jy begin het nie?	◯ Zange/ soze # Never # Nooit nie<br>◯ Ngenyanga # Monthly # Maandeliks<br>◯ Ngeveki # Weekly # Weekliks<br>◯ Phantse mihla yonke # Almost daily # Byna daagliks<br>◯ Mihla yonke # Daily # Daagliks<br>(Ask the participant; and read the response options aloud)
Kukangaphi kunyaka odlulileyo apho uthe awakwazi ukwenza oko bekulindeleke ukwenze ngenxa yokuba wawusele.<br><br>How often in the past year have you not been able to do what was expected of you because you drank?<br><br>Hoe gereeld het jy die afgelope jaar nie kon doen wat van jou verwag word, omdat jy gedrink het nie?	◯ Zange/ soze # Never # Nooit nie<br>◯ Ngenyanga # Monthly # Maandeliks<br>◯ Ngeveki # Weekly # Weekliks<br>◯ Phantse mihla yonke # Almost daily # Byna daagliks<br>◯ Mihla yonke # Daily # Daagliks<br>(Ask the participant; and read the response options aloud)
Kukangaphi kunyaka ogqithileyo apho uthe wasela ungatyanga ukuze ukwazi uzinyanga ngokuba usele ngamandla kwisuku eligqithileyo.<br><br>How often in the past year have you had to drink on an empty stomach in order to recover after drinking too much the previous day?<br><br>Hoe gereeld moes jy die afgelope jaar op 'n leë maag drink om te herstel nadat jy die vorige dag te veel gedrink het?	◯ Zange/ soze # Never # Nooit nie<br>◯ Ngenyanga # Monthly # Maandeliks<br>◯ Ngeveki # Weekly # Weekliks<br>◯ Phantse mihla yonke # Almost daily # Byna daagliks<br>◯ Mihla yonke # Daily # Daagliks<br>(Ask the participant; and read the response options aloud)
Kukangaphi kunyaka ogqithileyo ubukhe wazisola okanye wanesazela ekubeni usele?<br><br>How often in the past year have you felt remorse or guilt after drinking?<br><br>Hoe gereeld het jy berou of skuldgevoelens ervaar in die afgelope jaar nadat jy gedrink het?	◯ Zange/ soze # Never # Nooit nie<br>◯ Ngenyanga # Monthly # Maandeliks<br>◯ Ngeveki # Weekly # Weekliks<br>◯ Phantse mihla yonke # Almost daily # Byna daagliks<br>◯ Mihla yonke # Daily # Daagliks<br>(Ask the participant; and read the response options aloud)
Kukangaphi kunyaka ogqithileyo apho uthe awakwazi ukukhumbula ukuba kwenzeke ntoni kwisuku elingaphambili ngenxa yokuba ubusela.<br><br>How often in the past year have you been unable to remember what happened the night before because you had been drinking?<br><br>Vir die afgelope jaar wanneer jy gedink het, hoe gereeld kon jy nie onthou wat die vorige aand gebeur het nie?	◯ Zange/ soze # Never # Nooit nie<br>◯ Ngenyanga # Monthly # Maandeliks<br>◯ Ngeveki # Weekly # Weekliks<br>◯ Phantse mihla yonke # Almost daily # Byna daagliks<br>◯ Mihla yonke # Daily # Daagliks<br>(Ask the participant; and read the response options aloud)

For peer review only

Wakhe wena okanye omnye umntu walimala ngenxa yokuba ubusela?<br><br>Have you or another person gotten injured because you had been drinking?<br><br>Was jy of iemand anders beseer omdat jy gedrink het?	◯ Hayi # No # Nee<br>◯ Ewe kodwa hayi kunyaka ogqithileyo # Yes but not in the past year # Ja maar nie in die afgelope jaar<br>◯ Ewe kulonyaka ugqithileyo # Yes in the past year # Ja in die afgelope jaar<br>(Ask the participant; and read the response options aloud)
Bekukhe kwakho umntu omaziyo (umhlobo, ugqirha or umntu oqesqeshiweyo) wakhe waveza inkxalabo ngokuselwa kwakho utywala okanye wakucebisa ukuba yeka ukusela?<br><br>Has someone you know (friend, doctor, or professional) been concerned about your consumption of alcohol or has suggested that you stop drinking?<br><br>Was iemand wat jy ken (vriend, dokter of professionele persoon) besorg oor jou alkoholverbruik of het iemand voorgestel dat jy ophou drink?	◯ Hayi # No # Nee<br>◯ Ewe kodwa hayi kunyaka ogqithileyo # Yes but not in the past year # Ja maar nie in die afgelope jaar<br>◯ Ewe kulonyaka ugqithileyo # Yes in the past year # Ja in die afgelope jaar<br>(Ask the participant; and read the response options aloud)
**Drug Use**	
Kubalulekile ukuba sikubuze imibuzo ethile malunga nokusebenza iziyobisi. Iimpendulo zakho zizobasemfihlweni, ngoko uyacelwa ukuba unyaniseke. Nakanye wakhe wathatha/wasebenzisa esinye seziziyobisi (ngaphandle kwemvume/mpepha zika gqirha)? UYACELA UBHALE WAKHE WATHATHA KWAYE KANGANGAPHI.<br><br>It is important that we ask you certain questions about drug use. Your responses will be confidential, so please be honest. At least once have you taken any of these drugs (without medical prescription)? PLEASE MARK ALL THAT YOU HAVE TAKEN AND HOW OFTEN.<br><br>Dit is belangrik dat ons vir jou sekere vrae vra oor dwelmgebruik. Jou antwoorde sal vertroulik wees, dus wees eerlik. Het jy een van hierdie middels minstens een keer geneem (sonder 'n mediese voorskrif)? MERK ASSEBLIEF ALLES WAT JY GENEEM HET EN HOE GEREELD.	◯ Next<br>(Read to the participant)
Icuba (isigarethi/umdiza, inqawe, umdiza wesiga, icuba elihlafunwayo, inqawe, etc)<br><br>Tobacco (cigarettes, pipes, cigars, chewing tobacco, pipe, etc.)<br><br>Tabak (sigarette, pype, sigare, tabak kou, pyp, ens.)	◯ Zange/ soze # Never # Nooit nie<br>◯ kwanye kabini # 1 -2 times # 1-2 keer<br>◯ Nyanga nanyanga # Every month # Elke maand<br>◯ Kwiveki nganye # Every week # Elke week<br>◯ Mihla okanye phantse mihla yonke # Daily or almost daily # Daagliks of amper daagliks<br>(Ask the participant; and read the response options aloud)
Intsango<br><br>Cannabis (marijuana, hashish, etc.)<br><br>Cannabis (dagga, hasj, ens.)	◯ Zange/ soze # Never # Nooit nie<br>◯ kwanye kabini # 1 -2 times # 1-2 keer<br>◯ Nyanga nanyanga # Every month # Elke maand<br>◯ Kwiveki nganye # Every week # Elke week<br>◯ Mihla okanye phantse mihla yonke # Daily or almost daily # Daagliks of amper daagliks<br>(Ask the participant; and read the response options aloud)

For peer review only

Iziyobisi ezingumgubo omhlophe ((Intashi))<br><br>Cocaine (coca, crack, etc.)<br><br>Kokaïen (koka, kraak, ens.)	◯ Zange/ soze # Never # Nooit nie<br>◯ kwanye kabini # 1 -2 times # 1-2 keer<br>◯ Nyanga nanyanga # Every month # Elke maand<br>◯ Kwiveki nganye # Every week # Elke week<br>◯ Mihla okanye phantse mihla yonke # Daily or almost daily # Daagliks of amper daagliks<br>(Read to the participant; do not read the response options aloud)
Iziyobisi okanye ezinye izinto ezidungudelisa ingqondo (I ectstay, I -ice, Ritalini, liplisi zonciphisa umzimba, i-methamphetamine, njalo njalo).<br><br>Amphetamines or other stimulants (speed, ecstasy, ice, Ritalin, Dexedrine, slimming pills, methamphetamine, etc.)<br><br>Amfetamiene of ander stimulante (speed, ecstacy, ice, Ritalin, Dexedrine, verslankingspille, metamfetamien, ens.)	◯ Zange/ soze # Never # Nooit nie<br>◯ kwanye kabini # 1 -2 times # 1-2 keer<br>◯ Nyanga nanyanga # Every month # Elke maand<br>◯ Kwiveki nganye # Every week # Elke week<br>◯ Mihla okanye phantse mihla yonke # Daily or almost daily # Daagliks of amper daagliks<br>(Read to the participant; do not read the response options aloud)
Iziyobisi ezifunxwayo (iglu nezinye iziyobisi ezifunxwayo)<br><br>Inhalants (glues, terocal, gasoline / naphtha, glue, solvents, Poppers etc.)<br><br>Inhaleermiddels (gom, terokaal, petrol / nafta, gom, oplosmiddels, Poppers, ens.)	◯ Zange/ soze # Never # Nooit nie<br>◯ kwanye kabini # 1 -2 times # 1-2 keer<br>◯ Nyanga nanyanga # Every month # Elke maand<br>◯ Kwiveki nganye # Every week # Elke week<br>◯ Mihla okanye phantse mihla yonke # Daily or almost daily # Daagliks of amper daagliks<br>(Read to the participant; do not read the response options aloud)
Iziyobisi ezizipilisi<br><br>Tranquilizers or sleeping pills (valium / diazepam, Trankimazin / Alprazolam / Xanax, Orfidal / Lorazepam, Rohipnol, Librium, Ativan, GHB, Nembutal, Seconal, Phenobarbital, etc.)<br><br>Kalmeermiddels of slaappille (valium / diazepam, Trankimazin / Alprazolam / Xanax, Orfidal / Lorazepam, Rohypnol, Librium, Ativan, GHB, Nembutal, Seconal, Phenobarbital, ens.)	◯ Zange/ soze # Never # Nooit nie<br>◯ kwanye kabini # 1 -2 times # 1-2 keer<br>◯ Nyanga nanyanga # Every month # Elke maand<br>◯ Kwiveki nganye # Every week # Elke week<br>◯ Mihla okanye phantse mihla yonke # Daily or almost daily # Daagliks of amper daagliks<br>(Read to the participant; do not read the response options aloud)
Iziyobisi eziginywayo ((umgwinyo))<br><br>Hallucinogens (LSD, acids, ketamine, mezcalin, PCP, angel powder, etc.)<br><br>Hallucinogene (LSD, acids, ketamien, mezcalien, PCP, angel powder, ens.)	◯ Zange/ soze # Never # Nooit nie<br>◯ kwanye kabini # 1 -2 times # 1-2 keer<br>◯ Nyanga nanyanga # Every month # Elke maand<br>◯ Kwiveki nganye # Every week # Elke week<br>◯ Mihla okanye phantse mihla yonke # Daily or almost daily # Daagliks of amper daagliks<br>(Read to the participant; do not read the response options aloud)
Iziyobisi ezihlatywa ngenaliti<br><br>Opioids (heroin, methadone, codeine, morphine, dolantine / pethidine, OxyContin, Darvon, Vicodin, Dilaudid, Demerol, Lomotil, Percodan, etc.)<br><br>Opioïede (heroïne, metadoon, kodeïne, morfien, dolantien / petidien, OxyContin, Darvon, Vicodin, Dilaudid, Demerol, Lomotil, Percodan, ens.)	◯ Zange/ soze # Never # Nooit nie<br>◯ kwanye kabini # 1 -2 times # 1-2 keer<br>◯ Nyanga nanyanga # Every month # Elke maand<br>◯ Kwiveki nganye # Every week # Elke week<br>◯ Mihla okanye phantse mihla yonke # Daily or almost daily # Daagliks of amper daagliks<br>(Read to the participant; do not read the response options aloud)

For peer review only

Iziyobisi ezihlatywa ngenaliti<br><br>Opioids (heroin, methadone, codeine, morphine, dolantine / pethidine, OxyContin, Darvon, Vicodin, Dilaudid, Demerol, Lomotil, Percodan, etc.) Specify<br><br>Opioïede (heroïne, metadoon, kodeïne, morfien, dolantien / petidien, OxyContin, Darvon, Vicodin, Dilaudid, Demerol, Lomotil, Percodan, ens.) Spesifiseer	____________________<br>(If Other, specify)
Iimpendulo zakho kumele zigxile kwindlela yoziphatha kwakho kwiinyanga eziyi 12 ezidluleyo.<br><br>Your answers should be in response to your behavior over the past 12 months.<br><br>Jou antwoorde moet reageer op jou gedrag die afgelope 12 maande.	◯ Next<br>(Read to the participant; do not read the response options aloud)
Wawukhe wafumanisa ukuba kukho isidingo okanye ucacele ukuthatha iziyobizi ezi uzikhankanyileyo ngentla apho wawungakwazi ukuzibamba/ukuzinqanda?<br><br>Have you felt the need or desire to take the drugs you mentioned that you were unable to resist?<br><br>Het jy die behoefte of begeerte gevoel om die dwelms te gebruik wat jy genoem het?	◯ Ewe # Yes # Ja<br>◯ Hayi # No # Nee<br>◯ Khetha ungaphenduli # Prefer not to answer # Verkies om nie te antwoord nie<br>(Read to the participant; do not read the response options aloud)
Wawukhe wazama ukungathathi okanye ubukhe wanzinyelwa kukuyeka bambikokuba unxile/uyobe?<br><br>Have you tried to not take, or have you had difficulties with stopping before becoming intoxicated?<br><br>Het jy probeer om nie te gebruik nie, of het jy probleme gehad om op te hou voordat jy bedwelm geword het?	◯ Ewe # Yes # Ja<br>◯ Hayi # No # Nee<br>◯ Khetha ungaphenduli # Prefer not to answer # Verkies om nie te antwoord nie<br>(Read to the participant; do not read the response options aloud)

For peer review only

Xa uthatha iziyobisi ezimbalwa okanye ungazithathi kwa, uyaziva impawo zokuba awukhange uzithathe? (iintlungu, ukungcangcazela, umkhuhlane, ukuziva ungenamandla, utyatyazo, isizaphuzaphu, ukubetha kwentliziyo okugqithisileyo, ukusokola ulala, ukuziva udubekile, udinekile, okanye ungakhululeki ngokwengcinga? okanye, ubukhe wathatha enye into ukuze ungaziva ezimpawo?<br><br>When you take less drugs or do not take them at all, do you feel symptoms from withdrawal? (pain, shaking, fever, weak feelings, diarrhea, nausea, sweating, elevated heartbeat, difficulty sleeping, feeling agitated, irritable, or depressed? Or, have you taken another substance to avoid feeling these withdrawal symptoms?<br><br>As jy minder dwelms gebruik of glad nie dwelms gebruik nie, voel jy dan simptome van onttrekking? (pyn, bewe, koors, swak gevoelens, diarree, naarheid, sweet, vinnige hartklop, sukkel om te slaap, onrustig, geïrriteerd of depressief? Of het jy 'n ander middel gebruik om hierdie onttrekkingsimptome te vermy?	◯ Ewe # Yes # Ja<br>◯ Hayi # No # Nee<br>◯ Khetha ungaphenduli # Prefer not to answer # Verkies om nie te antwoord nie<br>(Read to the participant; do not read the response options aloud)
Ubukhe waqaphela ukuba kunyanzelekile uthathe umlinganiselo ongaphezulu weziyobisi ukuzo ufumane iziphumo ezifanayo nanjengakuqala?<br><br>Have you noticed that you have to take a higher dose of drugs to get the same effects has before?<br><br>Het jy opgemerk dat jy 'n hoër dosis dwelms moet neem om dieselfde gevolge te hê?	◯ Ewe # Yes # Ja<br>◯ Hayi # No # Nee<br>◯ Khetha ungaphenduli # Prefer not to answer # Verkies om nie te antwoord nie<br>(Read to the participant; do not read the response options aloud)
Ubukhe wenza imisebenzi engezantsi (ngexesha lakho, xa uphumle, okanye imisebenzi yemihla yonke) ngenxa yokuba usebenzisa iziyobisi?<br><br>Have you done less activities (in your free time, at rest, or daily activities) because you were taking drugs?<br><br>Het jy minder aktiwiteite gedoen (in jou vrye tyd, tydens rus, of daaglikse aktiwiteite) omdat jy dwelms gebruik het?	◯ Ewe # Yes # Ja<br>◯ Hayi # No # Nee<br>◯ Khetha ungaphenduli # Prefer not to answer # Verkies om nie te antwoord nie<br>(Read to the participant; do not read the response options aloud)
Ubukhe waqhubeka ngokuthatha iziyobisi ezi uthe wazikhankanya noxa uyazi ukuba iziphumo zazo zikwenza ingxaki emzimbeni kunye nasengqondweni?<br><br>Have you continued taking the drugs you mentioned even though you know they result in physical and psychological problems?<br><br>Het jy voortgegaan om die dwelms wat jy genoem het te gebruik, al weet jy dat dit lei tot fisiese en sielkundige probleme?	◯ Ewe # Yes # Ja<br>◯ Hayi # No # Nee<br>◯ Khetha ungaphenduli # Prefer not to answer # Verkies om nie te antwoord nie<br>(Read to the participant; do not read the response options aloud)

For peer review only

Ekubeni ugqibele ukuthatha iziyobisi ezi ubuzikhankanyile, ubukhe wanengxaki zempilo umzekelo ukuthatha umlinganiso ongaphezulu ngamandla, ukhohlokhohlo olungapheliyo, uxinaniseleko, izifo, iingxaki nesibindi sakho, okanye ukulimala?<br><br>Since you last took the drugs you mentioned, have you had health problems like accidental overdose, chronic cough, convulsions, infections, problems with your liver, or an injury?<br><br>Sedert jy laas die dwelms gebruik het wat jy genoem het, het jy gesondheidsprobleme ondervind soos toevallige oordosis, chroniese hoes, stuiptrekkings, infeksies, probleme met jou lewer of 'n besering?	◯ Ewe # Yes # Ja<br>◯ Hayi # No # Nee<br>◯ Khetha ungaphenduli # Prefer not to answer # Verkies om nie te antwoord nie<br>(Read to the participant; do not read the response options aloud)
Oko waqalayo ukuthatha iziyobisi, ubukhe wanazo iingxaki ngokusengqondweni, ezinjengokungabinamdla kwinto yonke, ukuziva ukhathazekile, ukungathembi abanye abantu, ukubonangcinga zokuba uzoxoxiswa okanye ezinye nje iingcinga ezingaqhelekanga?<br><br>Since you started taking drugs, have you had psychological problems, like not being interested in anything, feeling sad, not trusting other people, having thoughts of being persecuted or other strange thoughts?<br><br>Sedert jy dwelms begin gebruik het, het jy sielkundige probleme gehad, soos dat jy nie in iets belangstel nie, hartseer gevoel het, nie ander mense vertrou nie, gedagtes van vervolg of ander vreemde gedagtes gehad het?	◯ Ewe # Yes # Ja<br>◯ Hayi # No # Nee<br>◯ Khetha ungaphenduli # Prefer not to answer # Verkies om nie te antwoord nie<br>(Read to the participant; do not read the response options aloud)
Ubukhe waneengxaki eskolweni, emsebenzini, okanye ekhaya ngenxa yokuthatha iziyobisi ezi ozikhankanyileyo?<br><br>Have you had problems at school, at work, or at home because of taking the drugs you mentioned?<br><br>Het jy probleme gehad op skool, by die werk of by die huis as gevolg van die gebruik van die dwelms wat jy genoem het?	◯ Ewe # Yes # Ja<br>◯ Hayi # No # Nee<br>◯ Khetha ungaphenduli # Prefer not to answer # Verkies om nie te antwoord nie<br>(Read to the participant; do not read the response options aloud)

Physical Changes in Adolescence

For peer review only

Xa sishiya ubuntwana singena ekufikiseni, imizimba yethu ibanotshintsho, abanye abantu bazi lento ngokuba "bayafikisa". Kungenzeka ukuba lonke olutshintsho luchaphazele isigulo sakho sesifo sephepha, kodwa iseyinto esingenalwazi lwayo. Kunjalo nje, sinqwenela ukubuza imibuzo embalwa ngotshintsho emzimbeni wakho ngelaxesha uthe wagula sisifo sephepha. Ukuba awunafuni kuphendula, noko kulungile; siyayiqonda lemibuzo inganako ukwenza ungahlali kamnandi.<br><br>When we leave childhood and enter adolescence, our body undergoes certain physical changes, some people know this as "puberty." It is possible that these changes have impacted your TB illness, but it is something we still do not know. Therefore, we would like to ask you a few questions about the changes in your body at the time you became sick with TB. If you do not wish to respond, that is okay; we understand these questions could make you a little uncomfortable.<br><br>As ons die kinderjare verlaat en tienerjare betree ondergaan ons liggaam sekere fisieke veranderinge, sommige beskryf dit as 'puberteit'. Dit is moontlik dat hierdie veranderinge jou TB-siekte beïnvloed het, maar dit is iets wat ons nog nie weet nie. Daarom wil ons jou 'n paar vrae vra oor die veranderinge in jou liggaam gedurende die tyd toe jy siek geword het van TB. As jy nie wil reageer nie, is dit okay; ons verstaan dat hierdie vrae jou 'n bietjie ongemaklik kan maak.	◯ Next<br>(Read to the participant; do not read the response options aloud)
Ubusewuqalile ukuba sexesheni okanye ukuya engceni?<br><br>Have you had your first period or menstrual cycle?<br><br>Het jy jou eerste maandstonde of menstruele siklus gehad?	◯ Ewe # Yes # Ja<br>◯ Hayi # No # Nee<br>(Ask the participant)
Wawuneminyaka emingaphi ukuqala kwakho ukuya engceni okanye ukuba sexesheni?<br><br>At what age was your first period or menstrual cycle?<br><br>Op watter ouderdom was jou eerste maandstonde of menstruele siklus?	____________________<br>(Ask the participant)
Sizokubonisa imifanekiso (Jonga Imifanekiso kweye Spanish): Ungasixelela inqanaba lokukhula kwamabele wakho akulo ngoku.<br><br>We will present you with some images (SEE IMAGES ON SPANISH VERSION): Could you tell us the stage of development of your breasts right now<br><br>Ons gaan 'n paar prente aan jou voorstel (KYK PRENTE OP SPAANSE WEERGAWE): Kan jy vir ons nou die ontwikkelingstadium van jou borste vertel	◯ Iqondo 1 # Stage 1<br>◯ Iqondo 2y # Stage 2y<br>◯ Iqondo 3 # Stage 3<br>◯ Iqondo 4 # Stage 4<br>◯ Iqondo 5 # Stage 5

For peer review only

Ukhe waqaphela ukutshintsha kwelizwi lakho?<br><br>Have you noticed your voice changing?<br><br>Het jy opgelet dat jou stem verander het?	◯ Yes<br>◯ No<br>(Ask the participant)
Wawunangaphi ukuqaphela kwakho okukuqala utshintso kwilizwi lakho?<br><br>At what age did you notice this change in your voice?<br><br>Op watter ouderdom het jy hierdie verandering in jou stem opgemerk?	____________________<br>(Ask the Participant)
Sizokubonisa imifanekiso ethile (BONA IMIFANEKISO KWEYE SPANISH): ungasixelelea ngenqanaba lokukhula kwamalungu wakho womzimba afihlelekileyo (amalungu omzimba angasese noboya)<br><br>We will present you with some images (SEE IMAGES ON SPANISH VERSION):<br>Could you tell us the stage of development of your private parts (genitals and pubic hair)?<br><br>Ons bied vir jou 'n paar beelde aan (SIEN BEELDE OOR SPAANSE VERSIE):<br>Kan jy ons die stadium van ontwikkeling van jou private dele (geslagsdele en skaamhare) vertel?	◯ Iqondo 1 # Stage 1<br>◯ Iqondo 2y # Stage 2y<br>◯ Iqondo 3 # Stage 3<br>◯ Iqondo 4 # Stage 4<br>◯ Iqondo 5 # Stage 5

Sex Life

Lemibuzo ilandelayo imalunga nemeko yakho yezesondo. Siyayazi ukuba ukwabelana ngesondo yinxalenye ebalulekileyo yokukhula. Ulwazi malnuga nokuthatha inkxaxheba kwakho kwezesondo kungasinceda ekuqondeni indlela oyihambileyo kwezempilo.<br><br>The next set of questions are about your sex life. We know that sex is an important part of growing up. Your sex life can help us understand more about your health journey.<br><br>Die volgende stel vrae handel oor jou sekslewe. Ons weet dat seks 'n belangrike deel van grootword is. Jou sekslewe kan ons help om meer oor jou gesondheidsreis te verstaan.	◯ Next<br>(Read to the participant)
Wakhe wobelana ngesondo?<br><br>Have you ever had sex?<br><br>Het jy al ooit seks gehad?	◯ Ewe # Yes # Ja<br>◯ Hayi # No # Nee<br>(Ask the participant)
Loluphi uhlobo lwesondo owakhe walenza? (Khetha KONKE okufanelekileyo)<br><br>What type of sex have you had? (Select ALL that apply)<br><br>Watter tipe seks het jy al gehad? (Kies ALLES wat van toepassing is)	☐ Emlonyeni # Oral # Orale<br>☐ Ekukwini # Vaginal # Vaginale<br>☐ Anal Empundu # Anal # Anale<br>(Ask the participant)

For peer review only

Wawuneminyaka emingaphi ukuqala kwakho ukwabelana ngesondo?<br><br>How old were you when you first had sex?<br><br>Hoe oud was jy toe jy die eerste keer seks gehad het?	______________________<br>(Ask the participant)
Nabantu abantu abangaphi owathi wobelane ngesondo?<br><br>With how many people have you had sex?<br><br>Met hoeveel mense het jy al seks gehad?	◯ Umntu oyi I # I person # I persoon<br>◯ Abantu abayi 2 # 2 people # 2 mense<br>◯ Abantu abayi 3 # 3 people # 3 mense<br>◯ Abantu abayi 4 # 4 People # 4 mense<br>◯ Abantu abayi 5 # 5 people # 5 mense<br>◯ nye ngaphezulu abantu # 6 or more people # 6 of meer mense<br>(Ask the participant; and read the response options aloud)
Ngelaxesha ubugqibele ukwabelana ngesondo, wayisebenzisa i condom?<br><br>During the last time you had sex, did you use a condom?<br><br>Het jy 'n kondoom gebruik gedurende die laaste keer wat jy seks gehad het?	◯ Ewe # Yes # Ja<br>◯ Hayi # No # Nee

Major Life Events

Abantwana abaninzi kunye nabantu abafikisayo baye babenamava akhathazayo anothi achaphazele impilo yabo nendlela yophila. Nganye kwezi ziqingatha zilandelayo uyacelwa uphendule ewe ukuba iyakufanela wena okanye ayifaneli wena.<br><br>Many children and adolescents have stressful experiences that may impact their health and well-being. For each of the following statements please answer yes if it applies to you or no if it does not.<br><br>Baie kinders en adolessente het stresvolle ervarings wat hul gesondheid en welstand kan beïnvloed. Antwoord asseblief ja vir elk van die volgende stellings as dit op u van toepassing is of nee as dit nie van toepassing is nie.	◯ Next<br>(Read to participant)
Abazali bakho okanye abagcini bakho baqhawule umtshato okanye bohlukene.<br><br>Your parents or guardians divorced or separated.<br><br>Jou ouers of voogde het geskei of is uitmekaar uit.	◯ Ewe # Yes # Ja<br>◯ Hayi # No # Nee<br>(Read to the participant; do not read the response options aloud)
Ukhe waphila nomntu obeke wabanjwa okanye waya ejele.<br><br>You lived with someone that was in jail or prison.<br><br>Jy het by iemand gewoon wat in die tronk of in die gevangenis was.	◯ Ewe # Yes # Ja<br>◯ Hayi # No # Nee<br>(Read to the participant; do not read the response options aloud)

For peer review only

Wakhe waphila nomntu onoxinizelelo ngokweengcinga, ukuphazamiseka entloko, okanye wazama ukuzibulala.<br><br>You lived with someone that suffered from depression, had a mental illness, or attempted suicide.<br><br>Jy het saam met iemand gewoon wat aan depressie gely het, 'n geestesongesteldheid/ geestesteuring gehad het of selfmoord gepoog het.	◯ Ewe # Yes # Ja<br>◯ Hayi # No # Nee<br>(Read to the participant; do not read the response options aloud)
Ukhe wabona okanye weva abantu obathembile bazilimaze okanye bazivise kabuhlungu.<br><br>You saw or heard people you trust hurt themselves or threaten to hurt themselves.<br><br>Jy het gesien of gehoor dat mense wat jy vertrou, hulself seermaak of dreig om hulself te beseer.	◯ Ewe # Yes # Ja<br>◯ Hayi # No # Nee<br>(Read to the participant; do not read the response options aloud)
Umntu omthembileyo owaye wakrwada kuwe, wakuthuka, owakuhlazayo, okanye owagxekayo ngendlela eyakoyikisayo okanye owakoyikisayo ukuba ngendlela esesiqwini.<br><br>A person that you trust has been rude to you, insulted you, humiliated you, or criticized you in a way that scared you or made you afraid that you would be harmed in a physical manner.<br><br>'n Persoon wie jy vertrou was onbeskof teenoor jou, het jou beledig, verneder of jou gekritiseer op 'n manier wat jou bang gemaak het, of jou bang gemaak het dat jy op 'n fisieke wyse skade aangedoen sou word.	◯ Ewe # Yes # Ja<br>◯ Hayi # No # Nee<br>(Read to the participant; do not read the response options aloud)
Umntu owakubamba kumalungu omzimba angasese okanye wakubuza ukuba bamba awakhe amalungu omzimba angasese ngendlela edibenisele nesondo apho wawungafuni, ngaphandle kwemvume yayo, okanye eyakwenza ungakhululeki.<br><br>Someone touched you in your private parts or asked you to touch their private parts in a sexual manner that you did not want, against your will, or that made you feel uncomfortable.<br><br>Iemand het aan jou privaat dele geraak of jou gevra om hul privaatonderdele op 'n seksuele manier aan te raak wat teen jou wil was, of wat jou ongemaklik laat voel het.	◯ Ewe # Yes # Ja<br>◯ Hayi # No # Nee<br>(Read to the participant; do not read the response options aloud)
Ukhe wanqatyelwa kukutya, iimpahla, indawo yokuhlala okanye umntu wokukhusela.<br><br>You have lacked food, clothing, a place to live or someone to protect you.<br><br>Jy het 'n tekort aan kos, klere, 'n plek om te woon of iemand om jou te beskerm.	◯ Ewe # Yes # Ja<br>◯ Hayi # No # Nee<br>(Read to the participant; do not read the response options aloud)

For peer review only

Umntu wakutyhala, wakumba, wakuqhwaba, wajola into okanye akubethe ngamandla kwade kwashiyeka uphawo okanye ukudumba.<br><br>Someone pushed, grabbed, slapped, threw something or hit you with enough force that it left a mark or bruise.<br><br>Iemand stoot, gryp, klap, gooi iets of slaan jou met genoeg krag dat dit 'n merk of kneusplek agterlaat het.	◯ Ewe # Yes # Ja<br>◯ Hayi # No # Nee<br>(Read to the participant; do not read the response options aloud)
Ukhe wahlala nomntu obenengxaki yotywala okanye ingxaki yeziyobisi.<br><br>You lived with someone that had an alcoholic or drug problem.<br><br>Jy het saam met iemand gewoon wat 'n alkohol- of dwelmprobleem gehad het.	◯ Ewe # Yes # Ja<br>◯ Hayi # No # Nee<br>(Read to the participant; do not read the response options aloud)
Wakhe wava ukuba akhomntu ukuxhasayo, ukuthandayo, okanye ukukhuselwayo.<br><br>You have felt that no one is there to support, love, or protect you.<br><br>Jy het gevoel dat niemand daar is om jou te ondersteun, lief te hê of te beskerm nie.	◯ Ewe # Yes # Ja<br>◯ Hayi # No # Nee<br>(Read to the participant; do not read the response options aloud)

Experiences of stigma and discrimination for People living with TB and HIV

Singathanda ngoku ukukubuza imibuzo embalwa malunga namava wakho ngokuphile nesifo se TB. Uyacelwa ukhumbule ukuba oluluhlu lwemibuzo kunye neempendulo zakho zisemfihlakalweni nakwintembeko. Uyacelwa ucacise ukuba uvumelana kangakanani okanye awuvumelani nezintetha zilandelayo.<br><br>We would now like to ask you a few questions about your experiences living with TB. If you have indicated that you are living with HIV there will be a few questions regarding your experiences with HIV as well. Please remember that this survey and your responses are private and confidential. Please indicate how strongly you agree or disagree with the following statements.<br><br>Ons wil nou graag vir jou 'n paar vrae oor jou ervaring by TB. As jy aangedui het dat jy met MIV leef, sal daar ook 'n paar vrae wees rakende jou ervarings met MIV. Onthou dat hierdie opname en jou antwoorde privaat en vertroulik is. Dui asseblief aan hoe sterk jy saamstem of verskil met die volgende stellings.	◯ Next<br>(Read to the participant)

For peer review only

Abantu abaphila ne TB abakunyango lwe TB baphathwa kakuhle ngabanye kunabantu abaphila ne TB abangathathi amayeza.<br><br>People living with TB who are taking TB treatment are treated better by others than people living with TB who are not taking treatment.<br><br>Mense wat met TB leef en wat TB-behandeling gebruik, word beter behandel deur ander mense, as mense wat met TB leef wat nie behandeling gebruik nie.	◯ Uyavumelana ngamandla # Strongly agree # Stem heeltemal saam<br>◯ Uyavumelana # Agree # Stem saam<br>◯ Phakathi # Neutral # Neutraal<br>◯ Awuvumelani # Disagree # Stem nie saam nie<br>◯ Awuvumelani ngamandla # Strongly disagree # Stem geheel en al nie saam nie<br>(Read to the participant; do not read the response options aloud)
Abantu abaphila nesifo sika Gawulayo abathatha unyango baphatheka ngcono kunabantu abaphila nesifo sika Gawulayo abangathathi mayeza.<br><br>People living with HIV who are taking treatment are treated better by others than people living with HIV who are not taking treatment.<br><br>Mense wat met MIV leef en wat behandeling gebruik, word beter behandel deur ander mense, as mense wat met MIV leef wat nie behandeling gebruik nie.	◯ Uyavumelana ngamandla # Strongly agree # Stem heeltemal saam<br>◯ Uyavumelana # Agree # Stem saam<br>◯ Phakathi # Neutral # Neutraal<br>◯ Awuvumelani # Disagree # Stem nie saam nie<br>◯ Awuvumelani ngamandla # Strongly disagree # Stem geheel en al nie saam nie<br>(Read to the participant; do not read the response options aloud)
Ndiye ndayeka uhlonitshwa okanye ukuma kwingingqi ngenxa yokuba ndine TB.<br><br>I have lost respect or standing in the community because of having TB.<br><br>Ek het respek of posisie verloor in die gemeenskap omdat ek TB het.	◯ Uyavumelana ngamandla # Strongly agree # Stem heeltemal saam<br>◯ Uyavumelana # Agree # Stem saam<br>◯ Phakathi # Neutral # Neutraal<br>◯ Awuvumelani # Disagree # Stem nie saam nie<br>◯ Awuvumelani ngamandla # Strongly disagree # Stem geheel en al nie saam nie<br>(Read to the participant; do not read the response options aloud)
Ndiyazeya ngesiqo sam ngenxa ukuba ndine TB.<br><br>I think less of myself because of having TB.<br><br>Ek dink minder van myself as gevolg van TB.	◯ Uyavumelana ngamandla # Strongly agree # Stem heeltemal saam<br>◯ Uyavumelana # Agree # Stem saam<br>◯ Phakathi # Neutral # Neutraal<br>◯ Awuvumelani # Disagree # Stem nie saam nie<br>◯ Awuvumelani ngamandla # Strongly disagree # Stem geheel en al nie saam nie<br>(Read to the participant; do not read the response options aloud)
Ndiye ndaziva ndinentloni kuba ndine TB.<br><br>I have felt ashamed because of having TB.<br><br>Ek het skaam gevoel omdat ek TB het.	◯ Uyavumelana ngamandla # Strongly agree # Stem heeltemal saam<br>◯ Uyavumelana # Agree # Stem saam<br>◯ Phakathi # Neutral # Neutraal<br>◯ Awuvumelani # Disagree # Stem nie saam nie<br>◯ Awuvumelani ngamandla # Strongly disagree # Stem geheel en al nie saam nie<br>(Read to the participant; do not read the response options aloud)

For peer review only

Abantu bathetha ngam kakubi ngenxa ndine TB.<br><br>People have talked badly about me because of having TB.<br><br>Mense het sleg gepraat oor my as gevolg van TB.	◯ Zange/soze # Never # Nooit nie<br>◯ Kanye # Once # Een keer<br>◯ Amaxesha aliqela # A few times # 'n paar keer<br>◯ Maxesha amaninzi # Often # Gereeld<br>◯ Hayi kwinyanga eziyi 12 ezidlulileyo kodwa seyisuka ukwenziwa # Not in the last 12 months but have experienced before # Nie die afgelope twaalf maande nie maar het dit al vantevore beleef<br>◯ Ayingeni ndawo kum ngenxa akhomntu wazi isimo sam # Not applicable because no-one knows my status # Nie van toepassing nie want niemand weet wat my status is nie<br>(Ask the participant; and read the response options aloud)
Ndikhe ndakhuthukwa, ndahlukunyezwa kwaye/ okanye ndagrogriswa ngenxa ndine TB.<br><br>I have been verbally insulted, harassed and/or threatened because of having TB.<br><br>Ek is mondelings beledig, getreiter en/of gedreig omdat ek TB het.	◯ Zange/soze # Never # Nooit nie<br>◯ Kanye # Once # Een keer<br>◯ Amaxesha aliqela # A few times # 'n paar keer<br>◯ Maxesha amaninzi # Often # Gereeld<br>◯ Hayi kwinyanga eziyi 12 ezidlulileyo, kodwa seyisuka ukwenziwa # Not in the last 12 months but have experienced before # Nie die afgelope twaalf maande nie maar het dit al vantevore beleef<br>◯ Ayingeni ndawo kum ngenxa akhomntu wazi isimo sam # Not applicable because no-one knows my status # Nie van toepassing nie want niemand weet wat my status is nie<br>(Ask the participant; and read the response options aloud)
Ndikhe ndabethwa ngenxa yokuba ndine TB.<br><br>I have been physically assaulted because of having TB.<br><br>Ek is fisies aangerand omdat ek TB het.	◯ Zange/soze # Never # Nooit nie<br>◯ Kanye # Once # Een keer<br>◯ Amaxesha aliqela # A few times # 'n paar keer<br>◯ Maxesha amaninzi # Often # Gereeld<br>◯ Hayi kwinyanga eziyi 12 ezidlulileyo, kodwa seyisuka ukwenziwa # Not in the last 12 months but have experienced before # Nie die afgelope twaalf maande nie maar het dit al vantevore beleef<br>◯ Ayingeni ndawo kum ngenxa akhomntu wazi isimo sam # Not applicable because no-one knows my status # Nie van toepassing nie want niemand weet wat my status is nie<br>(Ask the participant; and read the response options aloud)
Ndiva ngokungathi abantu abantu abasafuni ukuchopha ecaleni kwam, umzekelo kwisithuthi sikawonke wonke, ecaweni okanye kwigumbi lokulindela ngenxa ndine TB.<br><br>I have felt that people have not wanted to sit next to me, for example on public transport, at church or in a waiting room because I have TB.<br><br>Ek het gevoel dat mense nie langs my wou sit nie, byvoorbeeld in openbare vervoer, in die kerk of in 'n wagkamer omdat ek TB het.	◯ Zange/soze # Never # Nooit nie<br>◯ Kanye # Once # Een keer<br>◯ Amaxesha aliqela # A few times # 'n paar keer<br>◯ Maxesha amaninzi # Often # Gereeld<br>◯ Hayi kwinyanga eziyi 12 ezidlulileyo, kodwa seyisuka ukwenziwa # Not in the last 12 months but have experienced before # Nie die afgelope twaalf maande nie maar het dit al vantevore beleef<br>◯ Ayingeni ndawo kum ngenxa akhomntu wazi isimo sam # Not applicable because no-one knows my status # Nie van toepassing nie want niemand weet wat my status is nie<br>(Ask the participant; and read the response options aloud)

For peer review only

Ndikhe andavunyelwa ukufumana uncedo lwezempilo ngenxa yokuba ndine TB.<br><br>I have been denied health services because of having TB.<br><br>Ek is van gesondheidsdienste geweier omdat ek TB het.	◯ Zange/soze # Never # Nooit nie<br>◯ Kanye # Once # Een keer<br>◯ Amaxesha aliqela # A few times # 'n paar keer<br>◯ Maxesha amaninzi # Often # Gereeld<br>◯ Hayi kwinyanga eziyi 12 ezidlulileyo, kodwa seyisuka ukwenziwa # Not in the last 12 months but have experienced before # Nie die afgelope twaalf maande nie maar het dit al vantevore beleef<br>◯ Ayingeni ndawo kum ngenxa akhomntu wazi isimo sam # Not applicable because no-one knows my status # Nie van toepassing nie want niemand weet wat my status is nie<br>(Ask the participant; and read the response options aloud)
Omnye umntu uchaze isimo sam ukuba ndine TB ngaphandle kwemvume yam.<br><br>Someone else disclosed me having TB without my permission.<br><br>Iemand anders het bekend gemaak dat ek TB het sonder my toestemming.	◯ Zange/soze # Never # Nooit nie<br>◯ Kanye # Once # Een keer<br>◯ Amaxesha aliqela # A few times # 'n paar keer<br>◯ Maxesha amaninzi # Often # Gereeld<br>◯ Hayi kwinyanga eziyi 12 ezidlulileyo, kodwa seyisuka ukwenziwa # Not in the last 12 months but have experienced before # Nie die afgelope twaalf maande nie maar het dit al vantevore beleef<br>◯ Ayingeni ndawo kum ngenxa akhomntu wazi isimo sam # Not applicable because no-one knows my status # Nie van toepassing nie want niemand weet wat my status is nie<br>(Ask the participant; and read the response options aloud)
Ndiziva ndineentloni kuba ndine TB.<br><br>I have felt ashamed because I have TB.<br><br>Ek het skaam gevoel omdat ek MIV het.	◯ Zange/soze # Never # Nooit nie<br>◯ Kanye # Once # Een keer<br>◯ Amaxesha aliqela # A few times # 'n paar keer<br>◯ Maxesha amaninzi # Often # Gereeld<br>◯ Hayi kwinyanga eziyi 12 ezidlulileyo, kodwa seyisuka ukwenziwa # Not in the last 12 months but have experienced before # Nie die afgelope twaalf maande nie maar het dit al vantevore beleef<br>◯ Ayingeni ndawo kum ngenxa akhomntu wazi isimo sam # Not applicable because no-one knows my status # Nie van toepassing nie want niemand weet wat my status is nie<br>(Ask the participant; and read the response options aloud)
Ndiphulukene nokuhlonitshwa okanye isidima ngenxa yobume bam be HIV.<br><br>I have lost respect or standing because of my HIV status.<br><br>Ek het respek of posisie verloor as gevolg van my MIV status.	◯ Zange/soze # Never # Nooit nie<br>◯ Kanye # Once # Een keer<br>◯ Amaxesha aliqela # A few times # 'n paar keer<br>◯ Maxesha amaninzi # Often # Gereeld<br>◯ Hayi kwinyanga eziyi 12 ezidlulileyo, kodwa seyisuka ukwenziwa # Not in the last 12 months but have experienced before # Nie die afgelope twaalf maande nie maar het dit al vantevore beleef<br>◯ Ayingeni ndawo kum ngenxa akhomntu wazi isimo sam # Not applicable because no-one knows my status # Nie van toepassing nie want niemand weet wat my status is nie<br>(Ask the participant; and read the response options aloud)

For peer review only

Ndiyazeya isiqu sam ngenxa yemeko yam ye HIV.<br><br>I think less of myself because of my HIV status.<br><br>Ek dink minder van myself as gevolg van my MIV-status.	◯ Zange/soze # Never # Nooit nie<br>◯ Kanye # Once # Een keer<br>◯ Amaxesha aliqela # A few times # 'n paar keer<br>◯ Maxesha amaninzi # Often # Gereeld<br>◯ Hayi kwinyanga eziyi 12 ezidlulileyo, kodwa seyisuka ukwenziwa # Not in the last 12 months but have experienced before # Nie die afgelope twaalf maande nie maar het dit al vantevore beleef<br>◯ Ayingeni ndawo kum ngenxa akhomntu wazi isimo sam # Not applicable because no-one knows my status # Nie van toepassing nie want niemand weet wat my status is nie<br>(Ask the participant; and read the response options aloud)
Ndi okanye ndikhe ndanxaxha ukuya kufumana iipilisi ze ARV kule ngingqi ndisebenza kuyo.<br><br>I am or have been reluctant to access ARV drugs in the community in which I work.<br><br>Ek is of was huiwerig om toegang tot ARV-medisyne te verkry in die gemeenskap waarin ek werk.	◯ Zange/soze # Never # Nooit nie<br>◯ Kanye # Once # Een keer<br>◯ Amaxesha aliqela # A few times # 'n paar keer<br>◯ Maxesha amaninzi # Often # Gereeld<br>◯ Hayi kwinyanga eziyi 12 ezidlulileyo, kodwa seyisuka ukwenziwa # Not in the last 12 months but have experienced before # Nie die afgelope twaalf maande nie maar het dit al vantevore beleef<br>◯ Ayingeni ndawo kum ngenxa akhomntu wazi isimo sam # Not applicable because no-one knows my status # Nie van toepassing nie want niemand weet wat my status is nie<br>(Ask the participant; and read the response options aloud)
Ndibanexhala ngokungamkelwa ngabantu endisebenza nabo ngenxa yemeko yam ye HIV.<br><br>I worry about not being accepted by my colleagues because of my HIV status.<br><br>Ek is bekommerd dat ek nie deur my kollegas aanvaar word as gevolg van my MIV-status nie.	◯ Zange/soze # Never # Nooit nie<br>◯ Kanye # Once # Een keer<br>◯ Amaxesha aliqela # A few times # 'n paar keer<br>◯ Maxesha amaninzi # Often # Gereeld<br>◯ Hayi kwinyanga eziyi 12 ezidlulileyo, kodwa seyisuka ukwenziwa # Not in the last 12 months but have experienced before # Nie die afgelope twaalf maande nie maar het dit al vantevore beleef<br>◯ Ayingeni ndawo kum ngenxa akhomntu wazi isimo sam # Not applicable because no-one knows my status # Nie van toepassing nie want niemand weet wat my status is nie<br>(Ask the participant; and read the response options aloud)
Ndibanexhala ngokungamkelwa ngabanye ngenxa yesimo sam se HIV.<br><br>I worry about not being accepted by others because of my HIV status.<br><br>Ek is bekommerd daaroor dat ek nie deur ander aanvaar sal word nie weens my MIV-status.	◯ Zange/soze # Never # Nooit nie<br>◯ Kanye # Once # Een keer<br>◯ Amaxesha aliqela # A few times # 'n paar keer<br>◯ Maxesha amaninzi # Often # Gereeld<br>◯ Hayi kwinyanga eziyi 12 ezidlulileyo, kodwa seyisuka ukwenziwa # Not in the last 12 months but have experienced before # Nie die afgelope twaalf maande nie maar het dit al vantevore beleef<br>◯ Ayingeni ndawo kum ngenxa akhomntu wazi isimo sam # Not applicable because no-one knows my status # Nie van toepassing nie want niemand weet wat my status is nie<br>(Ask the participant; and read the response options aloud)

For peer review only

Sisicelo ukuba usixelele kukangaphi kwezizinto zilandelayo ezakhe zakwehlela, okanye uku ucinga zakhe zenzeka kuwe, ngenxa (yeziphumo) zemo yakho ye ntsholongwane ka Gawulayo kwiinyanga eziyi 12 ezidlulileyo.<br><br>Please tell us how often the following things have happened to you, or whether you think they have happened to you, because of (as a result of) your HIV status in the last 12 months?<br><br>Vertel ons hoe gereeld die volgende dinge met jou gebeur het, of, omdat jy dink dat dit met jou gebeur het as gevolg van jou MIV-status die afgelope 12 maande?	◯ Next<br>(Read to the participant)
Abantu bathetha kakubi ngam ngenxa yesimo sam se HIV.<br><br>People have talked badly about me because of my HIV status.<br><br>Mense het sleg gepraat oor my as gevolg van my MIV-status.	◯ Zange/soze # Never # Nooit nie<br>◯ Kanye # Once # Een keer<br>◯ Amaxesha aliqela # A few times # 'n paar keer<br>◯ Maxesha amaninzi # Often # Gereeld<br>◯ Hayi kwinyanga eziyi 12 ezidlulileyo, kodwa seyisuka ukwenziwa # Not in the last 12 months but have experienced before # Nie die afgelope twaalf maande nie maar het dit al vantevore beleef<br>◯ Ayingeni ndawo kum ngenxa akhomntu wazi isimo sam # Not applicable because no-one knows my status # Nie van toepassing nie want niemand weet wat my status is nie<br>(Ask the participant; and read the response options aloud)
Xa ndisiya kufuna unyango kwiziko lwezempilo, abasebenzi bezempilo bathetha kakubi ngam ngenxa yesimo sam se HIV.<br><br>When I have sought care at a health facility, healthcare workers talked badly about me because of my HIV status.<br><br>As ek by 'n gesondheidsinstelling sorg versoek het, het gesondheidsorgwerkers sleg gepraat oor my as gevolg van my MIV-status.	◯ Zange/soze # Never # Nooit nie<br>◯ Kanye # Once # Een keer<br>◯ Amaxesha aliqela # A few times # 'n paar keer<br>◯ Maxesha amaninzi # Often # Gereeld<br>◯ Hayi kwinyanga eziyi 12 ezidlulileyo, kodwa seyisuka ukwenziwa # Not in the last 12 months but have experienced before # Nie die afgelope twaalf maande nie maar het dit al vantevore beleef<br>◯ Ayingeni ndawo kum ngenxa akhomntu wazi isimo sam # Not applicable because no-one knows my status # Nie van toepassing nie want niemand weet wat my status is nie<br>(Read to the participant; do not read the response options aloud)
Ndikhe ndathukwa, ndahlukunyezwa kwaye/okanye ndagrogriswa ngenxa yesimo sam se HIV.<br><br>I have been verbally insulted, harassed and/or threatened because of my HIV status.<br><br>Ek is mondelings beledig, getreiter en/of gedreig weens my MIV-status.	◯ Zange/soze # Never # Nooit nie<br>◯ Kanye # Once # Een keer<br>◯ Amaxesha aliqela # A few times # 'n paar keer<br>◯ Maxesha amaninzi # Often # Gereeld<br>◯ Hayi kwinyanga eziyi 12 ezidlulileyo, kodwa seyisuka ukwenziwa # Not in the last 12 months but have experienced before # Nie die afgelope twaalf maande nie maar het dit al vantevore beleef<br>◯ Ayingeni ndawo kum ngenxa akhomntu wazi isimo sam # Not applicable because no-one knows my status # Nie van toepassing nie want niemand weet wat my status is nie<br>(Read to the participant; do not read the response options aloud)

For peer review only

Ndakhe ndabethwa ngenxa yesimo sam se HIV.<br><br>I have been physically assaulted because of my HIV status.<br><br>Ek is fisies aangerand weens my MIV-status.	◯ Zange/soze # Never # Nooit nie<br>◯ Kanye # Once # Een keer<br>◯ Amaxesha aliqela # A few times # 'n paar keer<br>◯ Maxesha amaninzi # Often # Gereeld<br>◯ Hayi kwinyanga eziyi 12 ezidlulileyo, kodwa seyisuka ukwenziwa # Not in the last 12 months but have experienced before # Nie die afgelope twaalf maande nie maar het dit al vantevore beleef<br>◯ Ayingeni ndawo kum ngenxa akhomntu wazi isimo sam # Not applicable because no-one knows my status # Nie van toepassing nie want niemand weet wat my status is nie<br>(Read to the participant; do not read the response options aloud)
Ndikhe ndava ukuba abantu bakhe abafuna ukuchopha ecaleni kwam, umzekelo kwisithuthi sikawonke wonke, ecaweni okanye kwigumbi lokulindela ngenxa yesimo sam se HIV.<br><br>I have felt that people have not wanted to sit next to me, for example on public transport, at church or in a waiting room because of my HIV status.<br><br>Ek het gevoel dat mense nie langs my wou sit nie weens my MIV-status, byvoorbeeld in openbare vervoer, in die kerk of in 'n wagkamer.	◯ Zange/soze # Never # Nooit nie<br>◯ Kanye # Once # Een keer<br>◯ Amaxesha aliqela # A few times # 'n paar keer<br>◯ Maxesha amaninzi # Often # Gereeld<br>◯ Hayi kwinyanga eziyi 12 ezidlulileyo, kodwa seyisuka ukwenziwa # Not in the last 12 months but have experienced before # Nie die afgelope twaalf maande nie maar het dit al vantevore beleef<br>◯ Ayingeni ndawo kum ngenxa akhomntu wazi isimo sam # Not applicable because no-one knows my status # Nie van toepassing nie want niemand weet wat my status is nie<br>(Read to the participant; do not read the response options aloud)
Omnye umntu wachaza isimo same se HIV ngaphandle kwemvume yam.<br><br>Someone else disclosed my HIV status without my permission.<br><br>Iemand anders het my MIV-status bekend gemaak sonder my toestemming.	◯ Zange/soze # Never # Nooit nie<br>◯ Kanye # Once # Een keer<br>◯ Amaxesha aliqela # A few times # 'n paar keer<br>◯ Maxesha amaninzi # Often # Gereeld<br>◯ Hayi kwinyanga eziyi 12 ezidlulileyo, kodwa seyisuka ukwenziwa # Not in the last 12 months but have experienced before # Nie die afgelope twaalf maande nie maar het dit al vantevore beleef<br>◯ Ayingeni ndawo kum ngenxa akhomntu wazi isimo sam # Not applicable because no-one knows my status # Nie van toepassing nie want niemand weet wat my status is nie<br>(Read to the participant; do not read the response options aloud)
Xa ndithe ndayofuna uncedo kwiziko lwezempilo, umsebenzi wezempilo wachaza isimo sam se HIV ngaphandle kwemvume.<br><br>When I have sought care at a health facility, a health worker disclosed my HIV status without my permission.<br><br>Nadat ek by 'n gesondheidsinstelling sorg versoek het, het 'n gesondheidsorgwerker my MIV-status bekend gemaak sonder my toestemming.	◯ Zange/soze # Never # Nooit nie<br>◯ Kanye # Once # Een keer<br>◯ Amaxesha aliqela # A few times # 'n paar keer<br>◯ Maxesha amaninzi # Often # Gereeld<br>◯ Hayi kwinyanga eziyi 12 ezidlulileyo, kodwa seyisuka ukwenziwa # Not in the last 12 months but have experienced before # Nie die afgelope twaalf maande nie maar het dit al vantevore beleef<br>◯ Ayingeni ndawo kum ngenxa akhomntu wazi isimo sam # Not applicable because no-one knows my status # Nie van toepassing nie want niemand weet wat my status is nie<br>(Read to the participant; do not read the response options aloud)

For peer review only

Ndiye andavunyelwa ukufumana iinkonzo zezempilo ngenxa yemo yam ye HIV.<br><br>I have been denied health services because of my HIV status.<br><br>Ek is gesondheidsdienste geweier weens my MIV-status.	◯ Zange/soze # Never # Nooit nie<br>◯ Kanye # Once # Een keer<br>◯ Amaxesha aliqela # A few times # 'n paar keer<br>◯ Maxesha amaninzi # Often # Gereeld<br>◯ Hayi kwinyanga eziyi 12 ezidlulileyo, kodwa seyisuka ukwenziwa # Not in the last 12 months but have experienced before # Nie die afgelope twaalf maande nie maar het dit al vantevore beleef<br>◯ Ayingeni ndawo kum ngenxa akhomntu wazi isimo sam # Not applicable because no-one knows my status # Nie van toepassing nie want niemand weet wat my status is nie<br>(Read to the participant; do not read the response options aloud)
Abantu endisebenza nabo bathethe kakubi ngam ngenxa yemo yam ye HIV.<br><br>My co-workers have talked badly about me because of my HIV status.<br><br>My medewerkers het sleg gepraat oor my as gevolg van my MIV-status.	◯ Zange/soze # Never # Nooit nie<br>◯ Kanye # Once # Een keer<br>◯ Amaxesha aliqela # A few times # 'n paar keer<br>◯ Maxesha amaninzi # Often # Gereeld<br>◯ Hayi kwinyanga eziyi 12 ezidlulileyo, kodwa seyisuka ukwenziwa # Not in the last 12 months but have experienced before # Nie die afgelope twaalf maande nie maar het dit al vantevore beleef<br>◯ Ayingeni ndawo kum ngenxa akhomntu wazi isimo sam # Not applicable because no-one knows my status # Nie van toepassing nie want niemand weet wat my status is nie<br>(Read to the participant; do not read the response options aloud)
Abantu abavumi ukufumana iinkonzo zezempilo kum ngenxa yemo yam ye HIV.<br><br>People have refused to receive health care services from me because of my HIV status.<br><br>Mense het geweier om gesondheidsorgdienste van my te ontvang weens my MIV-status.	◯ Zange/soze # Never # Nooit nie<br>◯ Kanye # Once # Een keer<br>◯ Amaxesha aliqela # A few times # 'n paar keer<br>◯ Maxesha amaninzi # Often # Gereeld<br>◯ Hayi kwinyanga eziyi 12 ezidlulileyo, kodwa seyisuka ukwenziwa # Not in the last 12 months but have experienced before # Nie die afgelope twaalf maande nie maar het dit al vantevore beleef<br>◯ Ayingeni ndawo kum ngenxa akhomntu wazi isimo sam # Not applicable because no-one knows my status # Nie van toepassing nie want niemand weet wat my status is nie<br>(Read to the participant; do not read the response options aloud)
Ndiye ndangqala, ndabuza, okanye umntu ebenyelisa kwaye/okanye ebandlulula ngakum.<br><br>I confronted, challenged, or educated someone who was stigmatising and/or discriminating against me.<br><br>Ek het iemand gekonfronteer, uitgedaag of opgevoed wat my stigmatiseer en/of diskrimineer.	◯ Zange/soze # Never # Nooit nie<br>◯ Kanye # Once # Een keer<br>◯ Amaxesha aliqela # A few times # 'n paar keer<br>◯ Maxesha amaninzi # Often # Gereeld<br>◯ Hayi kwinyanga eziyi 12 ezidlulileyo, kodwa seyisuka ukwenziwa # Not in the last 12 months but have experienced before # Nie die afgelope twaalf maande nie maar het dit al vantevore beleef<br>◯ Ayingeni ndawo kum ngenxa akhomntu wazi isimo sam # Not applicable because no-one knows my status # Nie van toepassing nie want niemand weet wat my status is nie<br>(Read to the participant; do not read the response options aloud)

For peer review only

Ndiluphelile okanye ndilizulisele ukulikhangela unyango ngenxa yezimilo zabasebenzi bezempilo malunga nabantu abaphila ne HIV.<br><br>I avoided or delayed seeking treatment because of the attitudes of health workers towards people living with HIV.<br><br>Ek het die versoek van behandeling vermy of vertraag weens gesondheidswerkers se houdings teenoor mense wat met MIV lee.	◯ Zange/soze # Never # Nooit nie<br>◯ Kanye # Once # Een keer<br>◯ Amaxesha aliqela # A few times # 'n paar keer<br>◯ Maxesha amaninzi # Often # Gereeld<br>◯ Hayi kwinyanga eziyi 12 ezidlulileyo, kodwa seyisuka ukwenziwa # Not in the last 12 months but have experienced before # Nie die afgelope twaalf maande nie maar het dit al vantevore beleef<br>◯ Ayingeni ndawo kum ngenxa akhomntu wazi isimo sam # Not applicable because no-one knows my status # Nie van toepassing nie want niemand weet wat my status is nie<br>(Read to the participant; do not read the response options aloud)
Umsebenzi kwezempilo uye wasebenzisa i gloves xa esenza uvavanyo olungadibenisenanga nanto zomzimba.<br><br>A health care worker used latex gloves for a non-invasive examination.<br><br>'n Gesondheidsorgwerker het latexhandskoene gebruik vir 'n nie-indringende ondersoek.	◯ Zange/soze # Never # Nooit nie<br>◯ Kanye # Once # Een keer<br>◯ Amaxesha aliqela # A few times # 'n paar keer<br>◯ Maxesha amaninzi # Often # Gereeld<br>◯ Hayi kwinyanga eziyi 12 ezidlulileyo, kodwa seyisuka ukwenziwa # Not in the last 12 months but have experienced before # Nie die afgelope twaalf maande nie maar het dit al vantevore beleef<br>◯ Ayingeni ndawo kum ngenxa akhomntu wazi isimo sam # Not applicable because no-one knows my status # Nie van toepassing nie want niemand weet wat my status is nie<br>(Read to the participant; do not read the response options aloud)
Ndicetyisiwe ukuba ndingabinaye (okanye ndingabi ngu tata) kumntu ngenxa yesimo sam se HIV.<br><br>I have been advised not to have (or to father) a child because of my HIV status.<br><br>Ek is aangeraai om nie 'n kind te hê (of te verwek) weens my MIV-status.	◯ Zange/soze # Never # Nooit nie<br>◯ Kanye # Once # Een keer<br>◯ Amaxesha aliqela # A few times # 'n paar keer<br>◯ Maxesha amaninzi # Often # Gereeld<br>◯ Hayi kwinyanga eziyi 12 ezidlulileyo, kodwa seyisuka ukwenziwa # Not in the last 12 months but have experienced before # Nie die afgelope twaalf maande nie maar het dit al vantevore beleef<br>◯ Ayingeni ndawo kum ngenxa akhomntu wazi isimo sam # Not applicable because no-one knows my status # Nie van toepassing nie want niemand weet wat my status is nie<br>(Read to the participant; do not read the response options aloud)

For peer review only

For peer review only

Asinothanda ukubuza uluhlu lwemibuzo olunxibelelana kubantu bakho abayinkxaso. Uyacelwa ukhethe impendulo eyiyeyona ifana namava wakho, ngokuphendula "ndiyavumelana ngamandla', 'ndiyavumelana', 'phakathi', 'andivumelani' okanye 'andivumelani ngamandla'<br><br>We would not like to ask you a series of questions related to your social support systems. Please choose the answer closest resembling your experience, by answering 'strongly agree', 'agree', 'neutral', 'disagree' or 'strongly disagree'.<br><br>Ons wil nie graag hê jy moet vra 'n reeks vrae wat verband hou met jou sosiale steunstelsels. Kies die antwoord wat die beste by jou ervaring lyk, deur '"stem heeltemal saam", "stem saam", "neutraal", "stem nie saamstem nie" of "stem heeltemal nie saam nie" te beantwoord.	◯ Next<br>(Read to the participant)
Ndiyifumanisa kulula ukuthetha nabantu malunga nesimo sam se TB.<br><br>I find it easy to speak to people about my TB.<br><br>Ek vind dit maklik om met mense oor my TB te praat.	◯ Uyavumelana ngamandla # Strongly agree # Stem heeltemal saam<br>◯ Uyavumelana # Agree # Stem saam<br>◯ Phakathi # Neutral # Neutraal<br>◯ Awuvumelani # Disagree # Stem nie saam nie<br>◯ Awuvumelani ngamandla # Strongly disagree # Stem geheel en al nie saam nie<br>(Ask the participant; do not read the response options aloud)
Ndiyifumanisa kulula ukuthetha nabantu malunga nonyango lwam okanye ngezempilo.<br><br>I find it easy to speak to people about my treatment or healthcare.<br><br>Ek vind dit maklik om met mense te praat oor my behandeling of gesondheidsorg.	◯ Uyavumelana ngamandla # Strongly agree # Stem heeltemal saam<br>◯ Uyavumelana # Agree # Stem saam<br>◯ Phakathi # Neutral # Neutraal<br>◯ Awuvumelani # Disagree # Stem nie saam nie<br>◯ Awuvumelani ngamandla # Strongly disagree # Stem geheel en al nie saam nie<br>(Ask the participant; do not read the response options aloud)
Ndiziva ngokungathi ndingaxelela abantu xa ndiziva ndinomsindo okanye ndikhathazekile.<br><br>I feel like I can tell people when I feel angry or sad.<br><br>Ek voel dat ek mense kan vertel as ek kwaad of hartseer voel.	◯ Uyavumelana ngamandla # Strongly agree # Stem heeltemal saam<br>◯ Uyavumelana # Agree # Stem saam<br>◯ Phakathi # Neutral # Neutraal<br>◯ Awuvumelani # Disagree # Stem nie saam nie<br>◯ Awuvumelani ngamandla # Strongly disagree # Stem geheel en al nie saam nie<br>(Ask the participant; do not read the response options aloud)
Ndiziva ingathi ndicela abantu bandixhase ngokongezekileyo okanye inkathalo.<br><br>I feel like I can ask people for more support or care.<br><br>Ek voel dat ek mense vir meer ondersteuning of versorging kan vra.	◯ Uyavumelana ngamandla # Strongly agree # Stem heeltemal saam<br>◯ Uyavumelana # Agree # Stem saam<br>◯ Phakathi # Neutral # Neutraal<br>◯ Awuvumelani # Disagree # Stem nie saam nie<br>◯ Awuvumelani ngamandla # Strongly disagree # Stem geheel en al nie saam nie<br>(Ask the participant; do not read the response options aloud)

For peer review only

Ndiva ngokungathi ukuphila kwam/ impilo yam ibalekile kubanto abasebomini bam.<br><br>I feel like my health is important to people in my life.<br><br>Ek voel dat my gesondheid belangrik is vir mense in my lewe.	◯ Uyavumelana ngamandla # Strongly agree # Stem heeltemal saam<br>◯ Uyavumelana # Agree # Stem saam<br>◯ Phakathi # Neutral # Neutraal<br>◯ Awuvumelani # Disagree # Stem nie saam nie<br>◯ Awuvumelani ngamandla # Strongly disagree # Stem geheel en al nie saam nie<br>(Ask the participant; do not read the response options aloud)
Ndiziva ndiqondwa xandixelela abantu nge TB yam.<br><br>I feel understood when I tell people about my TB.<br><br>Ek voel soos ek verstaan word wanneer ek mense vertel van my TB.	◯ Uyavumelana ngamandla # Strongly agree # Stem heeltemal saam<br>◯ Uyavumelana # Agree # Stem saam<br>◯ Phakathi # Neutral # Neutraal<br>◯ Awuvumelani # Disagree # Stem nie saam nie<br>◯ Awuvumelani ngamandla # Strongly disagree # Stem geheel en al nie saam nie<br>(Ask the participant; do not read the response options aloud)
Ndiziva ndiqondwa xandixelela abantu ngonyango lwam okanye ezempilo.<br><br>I feel understood when I tell people about my treatment or healthcare.<br><br>Ek voel soos ek verstaan word wanneer ek mense vertel van my behandeling of gesondheidsorg.	◯ Uyavumelana ngamandla # Strongly agree # Stem heeltemal saam<br>◯ Uyavumelana # Agree # Stem saam<br>◯ Phakathi # Neutral # Neutraal<br>◯ Awuvumelani # Disagree # Stem nie saam nie<br>◯ Awuvumelani ngamandla # Strongly disagree # Stem geheel en al nie saam nie<br>(Ask the participant; do not read the response options aloud)
Abantu bayamamela xandikhalaza nge TB yam.<br><br>People listen when I complain about my TB.<br><br>Mense luister as ek kla oor my TB.	◯ Uyavumelana ngamandla # Strongly agree # Stem heeltemal saam<br>◯ Uyavumelana # Agree # Stem saam<br>◯ Phakathi # Neutral # Neutraal<br>◯ Awuvumelani # Disagree # Stem nie saam nie<br>◯ Awuvumelani ngamandla # Strongly disagree # Stem geheel en al nie saam nie<br>(Ask the participant; do not read the response options aloud)
Ndiziva ndixhaswa/ndongiwe ngabantu abasempilweni yam xandithetha nabo nge TB yam.<br><br>I feel comforted/supported by people in my life when I speak to them about my TB.<br><br>Ek voel vertroos/ondersteun deur mense in my lewe as ek met hulle praat oor my TB.	◯ Uyavumelana ngamandla # Strongly agree # Stem heeltemal saam<br>◯ Uyavumelana # Agree # Stem saam<br>◯ Phakathi # Neutral # Neutraal<br>◯ Awuvumelani # Disagree # Stem nie saam nie<br>◯ Awuvumelani ngamandla # Strongly disagree # Stem geheel en al nie saam nie<br>(Ask the participant; do not read the response options aloud)
Ndiziva ndiwongiwe/ ndixhaswa ngabantu empilweni xandithetha nabo ngonyango lwam okanye ezempilo.<br><br>I feel comforted/supported by people in my life when I speak to them about my treatment or healthcare.<br><br>Ek voel vertroos/ondersteun deur mense in my lewe as ek met hulle praat oor my behandeling of gesondheidsorg.	◯ Uyavumelana ngamandla # Strongly agree # Stem heeltemal saam<br>◯ Uyavumelana # Agree # Stem saam<br>◯ Phakathi # Neutral # Neutraal<br>◯ Awuvumelani # Disagree # Stem nie saam nie<br>◯ Awuvumelani ngamandla # Strongly disagree # Stem geheel en al nie saam nie<br>(Ask the participant; do not read the response options aloud)

For peer review only

Ndiyathetha nabantu nangeziphi iimbandezelo okanye iintshutshiso endibanazo malunga ne TB yam.<br><br>I speak to people about any struggles or challenges I have when it comes to my TB.<br><br>Ek praat met mense oor enige struwelinge of uitdagings wat my TB veroorsaak.	◯ Uyavumelana ngamandla # Strongly agree # Stem heeltemal saam<br>◯ Uyavumelana # Agree # Stem saam<br>◯ Phakathi # Neutral # Neutraal<br>◯ Awuvumelani # Disagree # Stem nie saam nie<br>◯ Awuvumelani ngamandla # Strongly disagree # Stem geheel en al nie saam nie<br>(Ask the participant; do not read the response options aloud)
Ndiyathetha nabantu nge TB yam.<br><br>I speak to people about my TB.<br><br>Ek praat met mense oor my TB.	◯ Zange/ soze # Never # Nooit nie<br>◯ Ngenyanga # Monthly # Maandeliks<br>◯ Ngeveki # Weekly # Weekliks<br>◯ Phantse mihla yonke # Almost daily # Byna daagliks<br>◯ Mihla yonke # Daily # Daagliks<br>(Ask the participant; and read the response options aloud)
Ndiyathetha nabantu malunga nonyango lwam okanye ezempilo.<br><br>I speak to people about my treatment or healthcare.<br><br>Ek praat met mense oor my behandeling of gesondheidsorg.	◯ Zange/ soze # Never # Nooit nie<br>◯ Ngenyanga # Monthly # Maandeliks<br>◯ Ngeveki # Weekly # Weekliks<br>◯ Phantse mihla yonke # Almost daily # Byna daagliks<br>◯ Mihla yonke # Daily # Daagliks<br>(Ask the participant; and read the response options aloud)
Ndiyathetha nabantu ngembandezelo okanye iintshutshiso.<br><br>I speak to people about any struggles or challenges.<br><br>Ek praat met mense oor enige struwelinge of uitdagings.	◯ Zange/ soze # Never # Nooit nie<br>◯ Ngenyanga # Monthly # Maandeliks<br>◯ Ngeveki # Weekly # Weekliks<br>◯ Phantse mihla yonke # Almost daily # Byna daagliks<br>◯ Mihla yonke # Daily # Daagliks<br>(Ask the participant; and read the response options aloud)
Ndiyathetha nabantu ngezimvo zam.<br><br>I speak to people about my emotions/feelings.<br><br>Ek praat met mense oor my emosies/gevoelens.	◯ Zange/ soze # Never # Nooit nie<br>◯ Ngenyanga # Monthly # Maandeliks<br>◯ Ngeveki # Weekly # Weekliks<br>◯ Phantse mihla yonke # Almost daily # Byna daagliks<br>◯ Mihla yonke # Daily # Daagliks<br>(Ask the participant; and read the response options aloud)

For peer review only

For peer review only

Njengokuba ubukhe wacacisa uphila ne HIV. Singathanda ukubuza uluhlu lwemibuzo esondelelene nenkxaso yakho esuka ebantwini. Uyacelwa ukhethe impendulo ekufutshane efana namava wakho kwakhona "ndiyavumelana ngamandla", "ndiyavumelana", "phakathi", "andivumelani" okanye "andivumelani ngamandla".<br><br>As you have indicated you are living with HIV. We would now like to ask you a series of questions related to your social support systems. Please choose the answer closest resembling your experience again "strongly agree", "agree", "neutral", "disagree" or "strongly disagree".<br><br>Soos jy aangedui het, leef jy met MIV. Ons wil nou graag vir jou vra om 'n reeks vrae wat verband hou met jou sosiale steunstelsels. Kies weer die antwoord wat die beste by jou ervaring lyk, "stem heeltemal saam", "stem saam", "neutraal", "stem nie saamstem nie" of "stem heeltemal nie saam nie".	◯ Next<br>(Read to participant)
Ndifumanisa kulula ukuthetha nabantu ngemeko yam ye HIV.<br><br>I find it easy to speak to people about my HIV status.<br><br>Ek vind dit maklik om met mense oor my MIV-status te praat.	◯ Uyavumelana ngamandla # Strongly agree # Stem heeltemal saam<br>◯ Uyavumelana # Agree # Stem saam<br>◯ Phakathi # Neutral # Neutraal<br>◯ Awuvumelani # Disagree # Stem nie saam nie<br>◯ Awuvumelani ngamandla # Strongly disagree # Stem geheel en al nie saam nie<br>(Ask the participant; do not read the response options aloud)
Ndiziva ndiqondwa xa ndixelela abantu ngemo yam ye HIV.<br><br>I feel understood when I tell people about my HIV.<br><br>Ek voel verstaan as ek mense van my MIV vertel.	◯ Uyavumelana ngamandla # Strongly agree # Stem heeltemal saam<br>◯ Uyavumelana # Agree # Stem saam<br>◯ Phakathi # Neutral # Neutraal<br>◯ Awuvumelani # Disagree # Stem nie saam nie<br>◯ Awuvumelani ngamandla # Strongly disagree # Stem geheel en al nie saam nie<br>(Ask the participant; do not read the response options aloud)
Abantu bayamamela xa ndikhalaza nge HIV yam.<br><br>People listen when I complain about my HIV.<br><br>Mense luister as ek oor my MIV kla.	◯ Uyavumelana ngamandla # Strongly agree # Stem heeltemal saam<br>◯ Uyavumelana # Agree # Stem saam<br>◯ Phakathi # Neutral # Neutraal<br>◯ Awuvumelani # Disagree # Stem nie saam nie<br>◯ Awuvumelani ngamandla # Strongly disagree # Stem geheel en al nie saam nie<br>(Ask the participant; do not read the response options aloud)
Ndiziva ndixhaswa ngabantu ebomini bam xandithetha nabo malunga nemeko yam ye HIV.<br><br>I feel comforted/supported by people in my life when I speak to them about my HIV status.<br><br>Ek voel getroos / ondersteun deur mense in my lewe as ek met hulle praat oor my MIV-status.	◯ Uyavumelana ngamandla # Strongly agree # Stem heeltemal saam<br>◯ Uyavumelana # Agree # Stem saam<br>◯ Phakathi # Neutral # Neutraal<br>◯ Awuvumelani # Disagree # Stem nie saam nie<br>◯ Awuvumelani ngamandla # Strongly disagree # Stem geheel en al nie saam nie<br>(Ask the participant; do not read the response options aloud)

For peer review only

Ndithetha nabantu malunga neentsokolo zam okanye imiceli mngeni endibanayo ekubeni ndine HIV.<br><br>I speak to people about any struggles or challenges I have when it comes to my HIV.<br><br>Ek praat met mense oor enige probleme of uitdagings wat ek het as dit by my MIV kom.	◯ Uyavumelana ngamandla # Strongly agree # Stem heeltemal saam<br>◯ Uyavumelana # Agree # Stem saam<br>◯ Phakathi # Neutral # Neutraal<br>◯ Awuvumelani # Disagree # Stem nie saam nie<br>◯ Awuvumelani ngamandla # Strongly disagree # Stem geheel en al nie saam nie<br>(Ask the participant; do not read the response options aloud)
Ndithetha nabantu nge HIV yam.<br><br>I speak to people about my HIV.<br><br>Ek praat met mense oor my MIV.	◯ Uyavumelana ngamandla # Strongly agree # Stem heeltemal saam<br>◯ Uyavumelana # Agree # Stem saam<br>◯ Phakathi # Neutral # Neutraal<br>◯ Awuvumelani # Disagree # Stem nie saam nie<br>◯ Awuvumelani ngamandla # Strongly disagree # Stem geheel en al nie saam nie<br>(Ask the participant; do not read the response options aloud)

For peer review only

For peer review only

Ngoku singathanda ukubuza uluhlu lwemibuzo esondelelene nenkxaso yakho esuka ebantwini. Uyacelwa ukhethw impendulo ekufutshane namava wakho.<br><br>We would now like to ask you a series of questions related to your social support systems. Please choose the answer closest resembling your experience.<br><br>Ons wil nou vir jou 'n reeks vrae stel wat verband hou met jou sosiale ondersteuningstelsels. Kies die antwoord wat die beste by jou ervaring lyk.	◯ Next<br>(Read to the participant)
Ndisebenzisa u Facebook.<br><br>I use Facebook.<br><br>Ek gebruik Facebook.	◯ Zange/ soze # Never # Nooit nie<br>◯ Ngenyanga # Monthly # Maandeliks<br>◯ Ngeveki # Weekly # Weekliks<br>◯ Phantse mihla yonke # Almost daily # Byna daagliks<br>◯ Mihla yonke # Daily # Daagliks<br>(Ask the participant; and read the response options aloud)
Ndisebenzisa u WhatsApp.<br><br>I use WhatsApp.<br><br>Ek gebruik WhatsApp.	◯ Zange/ soze # Never # Nooit nie<br>◯ Ngenyanga # Monthly # Maandeliks<br>◯ Ngeveki # Weekly # Weekliks<br>◯ Phantse mihla yonke # Almost daily # Byna daagliks<br>◯ Mihla yonke # Daily # Daagliks<br>(Read to the participant; do not read the response options aloud)
Ndisebenzisa u Twitter.<br><br>I use Twitter.<br><br>Ek gebruik Twitter.	◯ Zange/ soze # Never # Nooit nie<br>◯ Ngenyanga # Monthly # Maandeliks<br>◯ Ngeveki # Weekly # Weekliks<br>◯ Phantse mihla yonke # Almost daily # Byna daagliks<br>◯ Mihla yonke # Daily # Daagliks<br>(Ask the participant; do not read the response options aloud)

For peer review only

Ndisebenzisa u Instagram.<br><br>I use Instagram.<br><br>Ek gebruik Instagram.	◯ Zange/ soze # Never # Nooit nie<br>◯ Ngenyanga # Monthly # Maandeliks<br>◯ Ngeveki # Weekly # Weekliks<br>◯ Phantse mihla yonke # Almost daily # Byna daagliks<br>◯ Mihla yonke # Daily # Daagliks<br>(Ask the participant; do not read the response options aloud)
Uyacelwa ukuba ubhale amanye amakhasi onxibelelwano owasebenzisayo.<br><br>Please list the other social media platforms that you use.<br><br>Lys asseblief die ander sosiale media-platforms wat jy gebruik.	______________________<br>(Ask the participant)
Ndibona ulwazi oluninzi nge TB ku Facebook.<br><br>I see a lot of information about TB on Facebook.<br><br>Ek sien baie inligting oor TB op Facebook.	◯ Uyavumelana ngamandla # Strongly agree # Stem heeltemal saam<br>◯ Uyavumelana # Agree # Stem saam<br>◯ Phakathi # Neutral # Neutraal<br>◯ Awuvumelani # Disagree # Stem nie saam nie<br>◯ Awuvumelani ngamandla # Strongly disagree # Stem geheel en al nie saam nie<br>(Ask the participant; and read the response options aloud)
Ndibona ulwazi oluninzi nge TB ku WhatsApp.<br><br>I see a lot of information about TB on WhatsApp.<br><br>Ek sien baie inligting oor TB op WhatsApp.	◯ Uyavumelana ngamandla # Strongly agree # Stem heeltemal saam<br>◯ Uyavumelana # Agree # Stem saam<br>◯ Phakathi # Neutral # Neutraal<br>◯ Awuvumelani # Disagree # Stem nie saam nie<br>◯ Awuvumelani ngamandla # Strongly disagree # Stem geheel en al nie saam nie<br>(Ask the participant; do not read the response options aloud)
Ndibona ulwazi oluninzi nge TB ku Twitter.<br><br>I see a lot of information about TB on Twitter.<br><br>Ek sien baie inligting oor TB op Twitter.	◯ Uyavumelana ngamandla # Strongly agree # Stem heeltemal saam<br>◯ Uyavumelana # Agree # Stem saam<br>◯ Phakathi # Neutral # Neutraal<br>◯ Awuvumelani # Disagree # Stem nie saam nie<br>◯ Awuvumelani ngamandla # Strongly disagree # Stem geheel en al nie saam nie<br>(Ask the participant; do not read the response options aloud)
Ndibona ulwazi oluninzi nge TB ku Instagram.<br><br>I see a lot of information about TB on Instagram.<br><br>Ek sien baie inligting oor TB op Instagram.	◯ Uyavumelana ngamandla # Strongly agree # Stem heeltemal saam<br>◯ Uyavumelana # Agree # Stem saam<br>◯ Phakathi # Neutral # Neutraal<br>◯ Awuvumelani # Disagree # Stem nie saam nie<br>◯ Awuvumelani ngamandla # Strongly disagree # Stem geheel en al nie saam nie<br>(Ask the participant; do not read the response options aloud)

For peer review only

Ndiyaluthemba ulwazi malunga ne TB endilibona ku Facebook.<br><br>I trust information about TB that I see on Facebook.<br><br>Ek vertrou die inligting oot TB wat ek op Facebook sien.	◯ Uyavumelana ngamandla # Strongly agree # Stem heeltemal saam<br>◯ Uyavumelana # Agree # Stem saam<br>◯ Phakathi # Neutral # Neutraal<br>◯ Awuvumelani # Disagree # Stem nie saam nie<br>◯ Awuvumelani ngamandla # Strongly disagree # Stem geheel en al nie saam nie<br>(Ask the participant; do not read the response options aloud)
Ndiyalithemba ulwazi malunga ne TB endilibona ku WhatsApp.<br><br>I trust information about TB that I see on WhatsApp.<br><br>Ek vertrou die inligting oor TB wat ek op WhatsApp sien.	◯ Uyavumelana ngamandla # Strongly agree # Stem heeltemal saam<br>◯ Uyavumelana # Agree # Stem saam<br>◯ Phakathi # Neutral # Neutraal<br>◯ Awuvumelani # Disagree # Stem nie saam nie<br>◯ Awuvumelani ngamandla # Strongly disagree # Stem geheel en al nie saam nie<br>(Ask the participant; do not read the response options aloud)
Ndiyalithemba ulwazi malunga ne TB endilibona ku Twitter.<br><br>I trust information about TB that I see on Twitter.<br><br>Ek vertrou die inligting oor TB wat ek op Twitter sien.	◯ Uyavumelana ngamandla # Strongly agree # Stem heeltemal saam<br>◯ Uyavumelana # Agree # Stem saam<br>◯ Phakathi # Neutral # Neutraal<br>◯ Awuvumelani # Disagree # Stem nie saam nie<br>◯ Awuvumelani ngamandla # Strongly disagree # Stem geheel en al nie saam nie<br>(Ask the participant; do not read the response options aloud)
Ndiyalithemba ulwazi malunga ne TB ondilibona ku Instagram.<br><br>I trust information about TB that I see on Instagram.<br><br>Ek vertrou die inligting oor TB wat ek op Instagram sien.	◯ Uyavumelana ngamandla # Strongly agree # Stem heeltemal saam<br>◯ Uyavumelana # Agree # Stem saam<br>◯ Phakathi # Neutral # Neutraal<br>◯ Awuvumelani # Disagree # Stem nie saam nie<br>◯ Awuvumelani ngamandla # Strongly disagree # Stem geheel en al nie saam nie<br>(Ask the participant; do not read the response options aloud)
Ndiyaloba/ ndiyalupapasha ulwazi nge TB ku Facebook.<br><br>I share information about TB on Facebook.<br><br>Ek deel inligting oor TB op Facebook.	◯ Uyavumelana ngamandla # Strongly agree # Stem heeltemal saam<br>◯ Uyavumelana # Agree # Stem saam<br>◯ Phakathi # Neutral # Neutraal<br>◯ Awuvumelani # Disagree # Stem nie saam nie<br>◯ Awuvumelani ngamandla # Strongly disagree # Stem geheel en al nie saam nie<br>(Ask the participant; do not read the response options aloud)
Ndiyalisasaza ulwazi nge TB ku WhatsApp.<br><br>I share information about TB on WhatsApp.<br><br>Ek deel inligting oor TB op WhatsApp.	◯ Uyavumelana ngamandla # Strongly agree # Stem heeltemal saam<br>◯ Uyavumelana # Agree # Stem saam<br>◯ Phakathi # Neutral # Neutraal<br>◯ Awuvumelani # Disagree # Stem nie saam nie<br>◯ Awuvumelani ngamandla # Strongly disagree # Stem geheel en al nie saam nie<br>(Ask the participant; do not read the response options aloud)

For peer review only

Ndiyalisasaza ulwazi nge TB ku Twitter.<br><br>I share information about TB on Twitter.<br><br>Ek deel inligting oor TB op Twitter.	◯ Uyavumelana ngamandla # Strongly agree # Stem heeltemal saam<br>◯ Uyavumelana # Agree # Stem saam<br>◯ Phakathi # Neutral # Neutraal<br>◯ Awuvumelani # Disagree # Stem nie saam nie<br>◯ Awuvumelani ngamandla # Strongly disagree # Stem geheel en al nie saam nie<br>(Ask the participant; do not read the response options aloud)
Ndiyalisasaza ulwazi nge TB ku Instagram.<br><br>I share information about TB on Instagram.<br><br>Ek deel inligting oor TB op Instagram.	◯ Uyavumelana ngamandla # Strongly agree # Stem heeltemal saam<br>◯ Uyavumelana # Agree # Stem saam<br>◯ Phakathi # Neutral # Neutraal<br>◯ Awuvumelani # Disagree # Stem nie saam nie<br>◯ Awuvumelani ngamandla # Strongly disagree # Stem geheel en al nie saam nie<br>(Ask the participant; do not read the response options aloud)
Kukhe kwenzeke ukuba ndithethe ngamava wam nge TB ku Facebook okanye WhatsApp okanye u Twitter okanye Instragram ngelixa ndigula<br><br>I am likely to talk about my experience of TB on Facebook or WhatsApp or Twitter or Instagram while I am ill.<br><br>Ek sal waarskynlik oor my ervaring van TB op Facebook of WhatsApp of Twitter of Instagram praat terwyl ek siek is.	◯ Uyavumelana ngamandla # Strongly agree # Stem heeltemal saam<br>◯ Uyavumelana # Agree # Stem saam<br>◯ Phakathi # Neutral # Neutraal<br>◯ Awuvumelani # Disagree # Stem nie saam nie<br>◯ Awuvumelani ngamandla # Strongly disagree # Stem geheel en al nie saam nie<br>(Ask the participant; do not read the response options aloud)
Kukhe kwenzeke ndithethe ngamava wam nge TB ku Facebook okanye WhatsApp okanye Instagram emva kokuba ndiphile kwakhona<br><br>I am likely to talk about my experience of TB on Facebook or WhatsApp or Twitter or Instagram after I am well again.<br><br>Ek sal waarskynlik oor my ervaring van TB op Facebook of WhatsApp of Twitter of Instagram praat nadat ek weer gesond is.	◯ Uyavumelana ngamandla # Strongly agree # Stem heeltemal saam<br>◯ Uyavumelana # Agree # Stem saam<br>◯ Phakathi # Neutral # Neutraal<br>◯ Awuvumelani # Disagree # Stem nie saam nie<br>◯ Awuvumelani ngamandla # Strongly disagree # Stem geheel en al nie saam nie<br>(Ask the participant; do not read the response options aloud)

For peer review only

For peer review only

Ngoku sizokuthanda ukubuza uluhlu lwemibuzo engqamene nobunjani bempilo yakho. Uyacelwa eyona mpendulo ekufutshane kumava wakho ngokuphendula ngenye yezi zilandelayo. "Maxesha onke", "mhila yonke", "Kanye okanye kabini evekini", "manqapha nqapha" okanye zange/soze".<br><br>We would now like to ask you a series of questions related to your quality of life. Please choose the answer closest resembling your experience by responding with one of the following: "All the time", "daily", "once or twice a week", "seldom" or" never".<br><br>Ons wil u nou 'n reeks vrae stel wat verband hou met u lewensgehalte. Kies die antwoord wat die beste by u ervaring lyk, deur op een van die volgende te reageer: 'Die hele tyd', 'daagliks', 'een of twee keer per week', 'selde' of 'nooit'.	◯ Next<br>(Read to the participant)
Uyacelwa usixelele ukuba kukangaphi apho uzive okanye ucinge kwinyanga egqithileyo (kwintsuku eziyi 30)?<br><br>Please tell us how often you've felt or thought the following in the last month (30 days)?<br><br>Vertel ons asseblief hoe gereeld jy die afgelope maand (30 dae) gevoel of gedink het?	◯ Next<br>(Read to the participant)
Ndiye ndafuna ulala okanye ulala ukodlula isiqhelo.<br><br>I wanted to sleep or slept more than usual.<br><br>Ek wou meer slaap as gewoonlik.	◯ Maxesha onke # All the time # Die hele tyd<br>◯ Mihla yonke # Daily # Daagliks<br>◯ Kanye okanye kabini ngeveki # Once or Twice a Week # Een of twee keer per week<br>◯ Inqabile # Seldom # Selde<br>◯ Zange/soze # Never # Nooit nie<br>(Ask the participant; and read the response options aloud)
Nditye kancinci okanye bendingafuni ukutya.<br><br>I ate less or didn't want to eat.<br><br>Ek het minder geëet of wou nie eet nie.	◯ Maxesha onke # All the time # Die hele tyd<br>◯ Mihla yonke # Daily # Daagliks<br>◯ Kanye okanye kabini ngeveki # Once or Twice a Week # Een of twee keer per week<br>◯ Inqabile # Seldom # Selde<br>◯ Zange/soze # Never # Nooit nie<br>(Ask the participant; do not read the response options aloud)
Ndiye ndaziva ndigula.<br><br>I felt sickly.<br><br>Ek het siek gevoel.	◯ Maxesha onke # All the time # Die hele tyd<br>◯ Mihla yonke # Daily # Daagliks<br>◯ Kanye okanye kabini ngeveki # Once or Twice a Week # Een of twee keer per week<br>◯ Inqabile # Seldom # Selde<br>◯ Zange/soze # Never # Nooit nie<br>(Ask the participant; do not read the response options aloud)

For peer review only

Ndizive ndingonwabanga.<br><br>I felt unhappy.<br><br>Ek het ongelukkig gevoel.	◯ Maxesha onke # All the time # Die hele tyd<br>◯ Mihla yonke # Daily # Daagliks<br>◯ Kanye okanye kabini ngeveki # Once or Twice a Week # Een of twee keer per week<br>◯ Inqabile # Seldom # Selde<br>◯ Zange/soze # Never # Nooit nie<br>(Ask the participant; do not read the response options aloud)
Ndiye ndaziva ndinesiyalo, ndidiniwe okanye ndingacacelanga ukwenza not.<br><br>I felt listless, bored or not like doing anything.<br><br>Ek het lusteloos gevoel, verveeld of nie lus om enigiets te doen nie.	◯ Maxesha onke # All the time # Die hele tyd<br>◯ Mihla yonke # Daily # Daagliks<br>◯ Kanye okanye kabini ngeveki # Once or Twice a Week # Een of twee keer per week<br>◯ Inqabile # Seldom # Selde<br>◯ Zange/soze # Never # Nooit nie<br>(Ask the participant; do not read the response options aloud)
Bendingafuni ukubona mntu.<br><br>I didn't want to see anybody.<br><br>Ek wou niemand sien nie.	◯ Maxesha onke # All the time # Die hele tyd<br>◯ Mihla yonke # Daily # Daagliks<br>◯ Kanye okanye kabini ngeveki # Once or Twice a Week # Een of twee keer per week<br>◯ Inqabile # Seldom # Selde<br>◯ Zange/soze # Never # Nooit nie<br>(Ask the participant; do not read the response options aloud)
Ndiye ndaziva ndingakhululekanga ukuba ndibonwe esidlangalaleni ngenxa yesimo sam se TB.<br><br>I felt uncomfortable being seen in public because of my TB.<br><br>Ek het ongemaklik gevoel om in die openbaar gesien te word as gevolg van my TB.	◯ Maxesha onke # All the time # Die hele tyd<br>◯ Mihla yonke # Daily # Daagliks<br>◯ Kanye okanye kabini ngeveki # Once or Twice a Week # Een of twee keer per week<br>◯ Inqabile # Seldom # Selde<br>◯ Zange/soze # Never # Nooit nie<br>(Ask the participant; do not read the response options aloud)
Khange ndithethe nabahlobo bam okanye amalungu osapho lwam.<br><br>I didn't speak to my friends or family members.<br><br>Ek het nie saam met my vriende of familielede gepraat nie.	◯ Maxesha onke # All the time # Die hele tyd<br>◯ Mihla yonke # Daily # Daagliks<br>◯ Kanye okanye kabini ngeveki # Once or Twice a Week # Een of twee keer per week<br>◯ Inqabile # Seldom # Selde<br>◯ Zange/soze # Never # Nooit nie<br>(Ask the participant; do not read the response options aloud)
Abazali bam bandiphathe ngokohlukileyo (njengomntana) ngenxa ye TB yam.<br><br>My parents treated me differently (like a child) because of my TB.<br><br>My ouers behandel my anders (soos 'n kind) as gevolg van my TB.	◯ Maxesha onke # All the time # Die hele tyd<br>◯ Mihla yonke # Daily # Daagliks<br>◯ Kanye okanye kabini ngeveki # Once or Twice a Week # Een of twee keer per week<br>◯ Inqabile # Seldom # Selde<br>◯ Zange/soze # Never # Nooit nie<br>(Ask the participant; do not read the response options aloud)

For peer review only

Abazali bam bandinqandile ekwenzeni izinto endifuna uzenza ngenxa ye TB yam.<br><br>My parents stopped me from doing things I wanted to do because of my TB.<br><br>My ouers het my verbied om dinge te doen wat ek wou doen vanweë my TB.	◯ Maxesha onke # All the time # Die hele tyd<br>◯ Mihla yonke # Daily # Daagliks<br>◯ Kanye okanye kabini ngeveki # Once or Twice a Week # Een of twee keer per week<br>◯ Inqabile # Seldom # Selde<br>◯ Zange/soze # Never # Nooit nie<br>(Ask the participant; do not read the response options aloud)
Ndiye ndafuna ukufihla isigulo sam okanye ndizifihle mna ngenxa ye TB yam.<br><br>I wanted to hide my illness or myself because of my TB.<br><br>Ek wou my siekte of myself wegsteek weens my TB.	◯ Maxesha onke # All the time # Die hele tyd<br>◯ Mihla yonke # Daily # Daagliks<br>◯ Kanye okanye kabini ngeveki # Once or Twice a Week # Een of twee keer per week<br>◯ Inqabile # Seldom # Selde<br>◯ Zange/soze # Never # Nooit nie<br>(Ask the participant; do not read the response options aloud)
Ndiye ndaziva ndishiyeka ngenxa ye TB yam.<br><br>I felt left out because of my TB.<br><br>Ek het uitgelaat gevoel weens my TB.	◯ Maxesha onke # All the time # Die hele tyd<br>◯ Mihla yonke # Daily # Daagliks<br>◯ Kanye okanye kabini ngeveki # Once or Twice a Week # Een of twee keer per week<br>◯ Inqabile # Seldom # Selde<br>◯ Zange/soze # Never # Nooit nie<br>(Ask the participant; do not read the response options aloud)
Ndiye ndakhalaza nge TB yam.<br><br>I complained about my TB.<br><br>Ek het gekla oor my TB.	◯ Maxesha onke # All the time # Die hele tyd<br>◯ Mihla yonke # Daily # Daagliks<br>◯ Kanye okanye kabini ngeveki # Once or Twice a Week # Een of twee keer per week<br>◯ Inqabile # Seldom # Selde<br>◯ Zange/soze # Never # Nooit nie<br>(Ask the participant; do not read the response options aloud)
Bekumele ndenze uhlelo ngobomi bam (okanye izigqibo) malunga ne TB yam.<br><br>I had to plan my life (or choices) around my TB.<br><br>Ek moes my lewe (of keuses) beplan rondom my TB.	◯ Maxesha onke # All the time # Die hele tyd<br>◯ Mihla yonke # Daily # Daagliks<br>◯ Kanye okanye kabini ngeveki # Once or Twice a Week # Een of twee keer per week<br>◯ Inqabile # Seldom # Selde<br>◯ Zange/soze # Never # Nooit nie<br>(Ask the participant; do not read the response options aloud)
Bendinengcwangu ngenxa ye TB yam.<br><br>I was in a bad mood because of my TB.<br><br>Ek was in 'n slegte bui weens my TB.	◯ Maxesha onke # All the time # Die hele tyd<br>◯ Mihla yonke # Daily # Daagliks<br>◯ Kanye okanye kabini ngeveki # Once or Twice a Week # Een of twee keer per week<br>◯ Inqabile # Seldom # Selde<br>◯ Zange/soze # Never # Nooit nie<br>(Ask the participant; do not read the response options aloud)

For peer review only

Ndizive ndisoyika okanye ndingaqinisekanga ngesiqu sam.<br><br>I felt scared or unsure of myself.<br><br>Ek het bang of onseker oor myself gevoel.	◯ Maxesha onke # All the time # Die hele tyd<br>◯ Mihla yonke # Daily # Daagliks<br>◯ Kanye okanye kabini ngeveki # Once or Twice a Week # Een of twee keer per week<br>◯ Inqabile # Seldom # Selde<br>◯ Zange/soze # Never # Nooit nie<br>(Ask the participant; do not read the response options aloud)
Ndizive ngokuba ndingakwazi uwafezekisa amaphupha endinawo ngesiqu sam.<br><br>I felt that I was able to accomplish goals I set for myself.<br><br>Ek het gevoel dat ek die doelwitte kon bereik wat ek vir myself gestel het.	◯ Maxesha onke # All the time # Die hele tyd<br>◯ Mihla yonke # Daily # Daagliks<br>◯ Kanye okanye kabini ngeveki # Once or Twice a Week # Een of twee keer per week<br>◯ Inqabile # Seldom # Selde<br>◯ Zange/soze # Never # Nooit nie<br>(Ask the participant; do not read the response options aloud)
Ndizive ndonwabile ngesiqu sam.<br><br>I felt pleased with myself.<br><br>Ek het tevrede met myself gevoel.	◯ Maxesha onke # All the time # Die hele tyd<br>◯ Mihla yonke # Daily # Daagliks<br>◯ Kanye okanye kabini ngeveki # Once or Twice a Week # Een of twee keer per week<br>◯ Inqabile # Seldom # Selde<br>◯ Zange/soze # Never # Nooit nie<br>(Ask the participant; do not read the response options aloud)
Ndandisoyika i appointment yam elandelayo yokuthatha unyango lwam.<br><br>I was afraid of my next appointment of taking my treatment.<br><br>Ek was bang vir my volgende afspraak om my behandeling te neem.	◯ Maxesha onke # All the time # Die hele tyd<br>◯ Mihla yonke # Daily # Daagliks<br>◯ Kanye okanye kabini ngeveki # Once or Twice a Week # Een of twee keer per week<br>◯ Inqabile # Seldom # Selde<br>◯ Zange/soze # Never # Nooit nie<br>(Ask the participant; do not read the response options aloud)
Ndikhe ndacinga ngobomi bam ukuba buzobanjani kwingomso ngenxa ye TB yam.<br><br>I thought about what my life is going to be like in future because of my TB.<br><br>Ek het gedink oor hoe my lewe in die toekoms gaan lyk as gevolg van my TB.	◯ Maxesha onke # All the time # Die hele tyd<br>◯ Mihla yonke # Daily # Daagliks<br>◯ Kanye okanye kabini ngeveki # Once or Twice a Week # Een of twee keer per week<br>◯ Inqabile # Seldom # Selde<br>◯ Zange/soze # Never # Nooit nie<br>(Ask the participant; do not read the response options aloud)
Khange ndikwazi ukwenza izinto ebendiqhele uzenza ngenxa ye TB yam.<br><br>I couldn't do things I used to be able to do because of my TB.<br><br>Ek kon, weens my TB, nie dinge doen wat ek vroeër kon doen nie.	◯ Maxesha onke # All the time # Die hele tyd<br>◯ Mihla yonke # Daily # Daagliks<br>◯ Kanye okanye kabini ngeveki # Once or Twice a Week # Een of twee keer per week<br>◯ Inqabile # Seldom # Selde<br>◯ Zange/soze # Never # Nooit nie<br>(Ask the participant; do not read the response options aloud)

For peer review only

Ndiphose ukuya eskolweni okanye khange ndikwazi ukwenza umsebenzi wam weskolo ngenxa ye TB yam.<br><br>I missed school or wasn't able to complete schoolwork because of my TB.<br><br>Ek het skool gemis of kon my skoolwerk nie voltooi nie as gevolg van my TB.	◯ Maxesha onke # All the time # Die hele tyd<br>◯ Mihla yonke # Daily # Daagliks<br>◯ Kanye okanye kabini ngeveki # Once or Twice a Week # Een of twee keer per week<br>◯ Inqabile # Seldom # Selde<br>◯ Zange/soze # Never # Nooit nie<br>(Ask the participant; do not read the response options aloud)
Bendixhala lokufumana i marks ezimbi eskolweni.<br><br>I am worried about getting bad marks at school.<br><br>Ek is bekommerd dat ek slegte punte sal behaal op skool.	◯ Maxesha onke # All the time # Die hele tyd<br>◯ Mihla yonke # Daily # Daagliks<br>◯ Kanye okanye kabini ngeveki # Once or Twice a Week # Een of twee keer per week<br>◯ Inqabile # Seldom # Selde<br>◯ Zange/soze # Never # Nooit nie<br>(Ask the participant; do not read the response options aloud)
Ukuba unganqwenela ukuthetha banzi nabaphathi boluphando malunga nayiphi enye yeengxaki, kunye/okanye ungathanda banxibelelane nawe ukuze bakuncede ufumane uncedo okanye inkxaso, bhala "ewe".<br><br>If you would like to talk more with the authorities of the study about any of these problems, and/or you would like that they contact you to help you find help or support, mark "Yes".<br><br>As jy wil graag meer praat met die owerhede van die studie oor enige van hierdie probleme, en / of jy wil graag dat hulle kontak om te help jy hulp of ondersteuning vind, merk "Ja".	◯ Ewe # Yes # Ja<br>◯ Hayi # No # Nee<br>(Ask the participant; do not read the response options aloud)

For peer review only
